# CTLs, a new class of RING-H2 ubiquitin ligases uncovered by YEELL, a motif close to the RING domain that is present across eukaryotes

**DOI:** 10.1371/journal.pone.0190969

**Published:** 2018-01-11

**Authors:** Domingo Jiménez-López, Laura Aguilar-Henonin, Juan Manuel González-Prieto, Victor Aguilar-Hernández, Plinio Guzmán

**Affiliations:** 1 Departamento de Ingeniería Genética, Centro de Investigación y de Estudios Avanzados del IPN, Unidad Irapuato, Irapuato, Guanajuato, México; 2 Biotecnología Vegetal, Centro de Biotecnología Genómica, Instituto Politécnico Nacional, Reynosa, Tamaulipas, México; 3 CONACYT, Unidad de Bioquímica y Biología Molecular de Plantas, Centro de Investigación Científica de Yucatán, Mérida, Yucatán, México; George Washington University, UNITED STATES

## Abstract

RING ubiquitin E3 ligases enclose a RING domain for ubiquitin ligase activity and associated domains and/or conserved motifs outside the RING domain that collectively facilitate their classification and usually reveal some of key information related to mechanism of action. Here we describe a new family of E3 ligases that encodes a RING-H2 domain related in sequence to the ATL and BTL RING-H2 domains. This family, named CTL, encodes a motif designed as YEELL that expands 21 amino acids next to the RING-H2 domain that is present across most eukaryotic lineages. E3 ubiquitin ligase BIG BROTHER is a plant CTL that regulates organ size, and SUMO-targeted ubiquitin E3 ligase RNF111/ARKADIA is a vertebrate CTL. Basal animal and vertebrate, as well as fungi species, encode a single CTL gene that constraints the number of paralogs observed in vertebrates. Conversely, as previously described in ATL and BTL families in plants, CTL genes range from a single copy in green algae and 3 to 5 copies in basal species to 9 to 35 copies in angiosperms. Our analysis describes key structural features of a novel family of E3 ubiquitin ligases as an integral component of the set of core eukaryotic genes.

## Introduction

Plant proteins frequently undergo modifications that modulate and modify their function. One such modifications is the covalent tagging with polypeptides having ubiquitin fold, characterized by a β-grasp fold with a flexible C-terminal tail. Ubiquitin (Ub), a 76-residues protein, is one example of a tag modifier almost invariant in all eukaryotes and was first described over 30 years ago [1]. Ub is a component of the ubiquitin proteasome system (UPS) which is a key path for the control of protein levels in the cell. In general, a cascade involving three types of enzymes (E1, ubiquitin activating; E2, ubiquitin conjugating and E3, ubiquitin ligase) directs the covalent attachment of the Ub to thousands of possible target proteins in the cell. Hence, a large family of E3 ligases facilitates Ub transfer by recruiting an E2 conjugase charged with an activated Ub and suitable-selected target proteins [2]

E3 ligase genes in plants greatly outnumber other eukaryotes. Nearly 1400 E3 ligase genes are annotated in the plant *Arabidopsis thaliana* genome and about 600 in the human genome [3, 4]. One prevalent domain in E3 ligases is the Zinc Finger known as REALLY INTERESTING NEW GENE (RING) that is required for catalysis. The RING domain consists of eight cysteine and histidine residues that bind to two zinc ions in a cross-brace structure and are found in a myriad of proteins with diverse domain architecture [5]. A class of E3 RING ligases that are highly represented in eukaryotic proteomes works as a selective scaffold-like fashion that facilitates the transfer of Ub from the E2-Ub bound at the RING domain to the target protein recognized outside the RING domain [4]. There are two major variants of the RING domain, the canonical RING or RING-HC, and the RING-H2 which contains a substitution of the fifth cysteine with a histidine residue [6]. There are 551 potential RING E3 ligases in *A*. *thaliana*, of which 45% correspond to RING-H2 variants. Similar proportion of RING-H2 variants has been predicted in other viridiplantae [3]. For instance, the proportion of RING-H2 variants in *Brassica rapa*, *Oryza sativa*, *Malus domestica* Borkh and in the green algae *Ostreococus tauri* are 52%, 57%, 55% and 53%, respectively [7–10].

Based on domain architecture, we previously described two families of E3 ligases that encode a highly similar RING-H2 domain and RING-outside conserved motifs. These two families are named Arabidopsis Tóxicos en Levadura (ATLs) and BCA2 Zinc-Finger (BZF) ATLs (BTLs) [11]. Almost all ATL members encode up to three transmembrane helices at the amino-terminus, 86% of which comprise a single helix. Indeed, the hydrophobic helix for some ATL proteins has been shown to be associated with the cell membranes [12]. An overview of the ATL family from grapevine and tomato have recently been reported [13, 14]. *BTL* genes encode a different domain at the amino-terminus, that includes four cysteine residues resembling a C2/C2 Zinc Finger, known as BZF. The co-occurrence of transmembrane helices or BZF motif with distinct RING-H2 variants suggests that selective pressures occurred throughout evolution to establish these families of E3 ligases.

During our initial analysis of RING-H2 proteins, we became aware of a protein group present in plants and human that encodes neither a transmembrane helix nor a BZF-like motif [15]. A multiple sequence alignment that includes those sequences and protein sequence from various mammalian species, displayed a unscattering conserved motif upstream from the RING-H2 domain that we named YEELL. To continue our study of the evolution and functional analysis of RING E3 ligases, we retrieved RING-H2 proteins that included the YEELL conserved motif from genome databases of plants, animals, fungi, and protozoa and collectively called as CTL E3 Ub ligase family. Interestingly, the YEELL conserved motif was found in the *A*. *thaliana* E3 ligase BIG BROTHER (BB), that regulates organ size; the human RNF111/ARKADIA, a SUMO-targeted ubiquitin E3 ligase (STUbL), a component of TGF-signaling and DNA damage response; RNF165/ARK2C that enhances Bone Morphogenetic Protein (BMP)-Smad signaling in motor axon extension; and RNF38 a regulator of p53 [16–19]. We identified 751 *CTL* genes from 110 genomes comprising plants, animals, protists and fungi species. Based on phylogenetic distribution and motif organization, plant CTL proteins were classified into eight groups. Our study describes the evolutionary history and conserved motif architecture of a new family of RING E3 ligases preserved across eukaryotes genomes.

## Results

### YEELL, a conserved motif as signature for CTL RING-H2 E3 ubiquitin ligases

**Fig 1:**
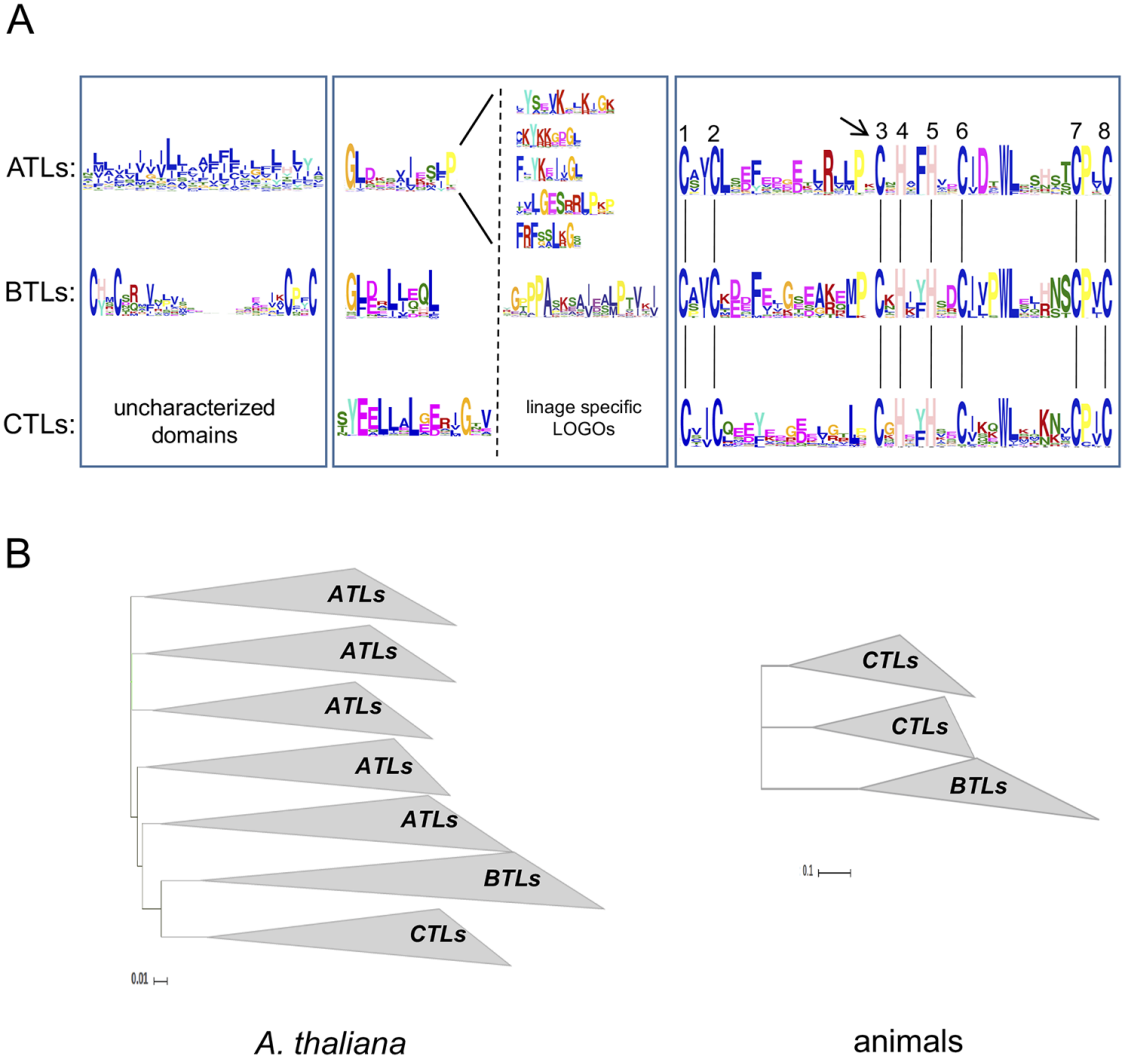
Overall domain structure features of ATL, BTL and CTL E3 ubiquitin ligases. A. Schematic representation of canonical ATL, BTL and CTL proteins indicating the position of the three relevant domains in panels and sequence LOGOs on each class: hydrophobic helices, GLD motif and RING-H2 in ATL proteins; BZF, GDL-like motif and RING-H2 in BTL proteins and uncharacterized domains, YEELL motif and RING-H2 in CTL proteins. Additional LOGOs are included within the middle panel: five adjacent to GLD (LOGOs 20, 26, 47 53 and 63 [12]), one adjacent to GDL-like (LOGO 2 [31]) and linage specific LOGOs adjacent to YEELL as displayed in Fig 6. Alignment of sequence LOGOs of the three RING-H2 domains is shown to the right; the residues involved in zinc ligation are numbered 1 to 8. The location of the highly conserved proline residue adjacent to the third cysteine in the RING-H2 domain is colored in yellow and the location of the amino acid residue between this proline and the third cysteine that is present in ATL proteins, but absent in BTL and CTLproteins is pointed out by the arrow. The GLD-like motif in BTL proteins corresponds to the previously reported sequence LOGO 6 in BTL proteins [11, 31]. B. Phylogenetic trees of *A*. *thaliana* ATL-, BTL- and CTL-RING-H2 domains (left) and animal BTL- and CTL-RING-H2 domains (right). The trees were generated by the Neighbor-Joining method with a bootstrap value of 1000 replicates. The tree from the left is based on the sequence of the RING domain from 75 ATLs, 17 BTLs and 19 CTL sequences; sequences were collapsed into seven branches. The tree from the right is based on the sequence of the RING domain from 21 BTLs and 38 CTL proteins from an assortment of animal species; sequences were collapsed into three branches (top, RNF165 and RNF111 orthologs; middle, RNF44 and RNF38 orthologs and bottom BTL orthologs.

We previously described two families of RING-H2 E3 ligases named ATL and BTL that showed co-occurrence of amino-terminus transmembrane helices or C2/C2 Zinc Finger with a RING-H2 domain for which the number of exclusive residues within the conserved eight metal ligands allow to distinguish between ATL and BTL domains. (Fig 1A). Three residues were restricted to ATL proteins including a Leu (L) downstream to the second metal ligand, an Arg (R) four residues upstream to the third metal ligand and an Asp (D) a residue downstream to the sixth metal ligand, while a Pro (P) residue to BTL located two residues downstream to the sixth metal ligand. Additionally, in parallel of an eight metal ligands-based RING-H2 protein curation data, we noticed in a multiple sequence alignment a new group of *A*. *thaliana* and human proteins that encode an scattering conserved motif upstream from the RING-H2 domain that could be a distinctive feature of new RING-H2 E3 ligases. This motif with nearly 21 amino acids displayed a signature of five consecutive residues denoted by Tyr (Y), 2x Glu (E), and 2x Leu (L) in at least 75% of the sequences analyzed; hence, this motif was named YEELL.” Following our previous terminology on two RING-H2 E3 ligases families, ATL and BTL, we named CTL as the gene family that encoded a YEELL motif upstream to an ATL-BTL-related RING-H2 domain. Although the presence of conserved motifs upstream from the RING-H2 domain was also observed in most ATL and BTL proteins, the composition were quite different to the identified in CTL proteins (see Fig 1A, LOGOs within the middle panel). Indeed, one motif called GLD, was retrieved from most ATL proteins and a similar motif in sequence (GLD-Like) was retrieved from most BTL proteins [11]. Phylogenetic analysis based on the RING-H2 amino acid sequences of *A*. *thaliana* ATL, BTL and CTL, protein sequences resolved five sister clades containing groups of ATL sequences as well as clades grouping BTL and CTL sequences (Fig 1B, left tree). Similar distribution was observed in trees based on the RING-H2 domains of BTL and CTL proteins from assorted animal species. Distinct clades were resolved, one including RNF44 and RNF38 orthologs, another RNF165 and RNF111 orthologs and one with the BTL orthologs (Fig 1B, right tree). In line of that RING-H2 domain has enough sequence information to sustain this clustering, no scrambled proteins were found within ATL, BTL or CTL phylogenetic distribution.

**Fig 2:**
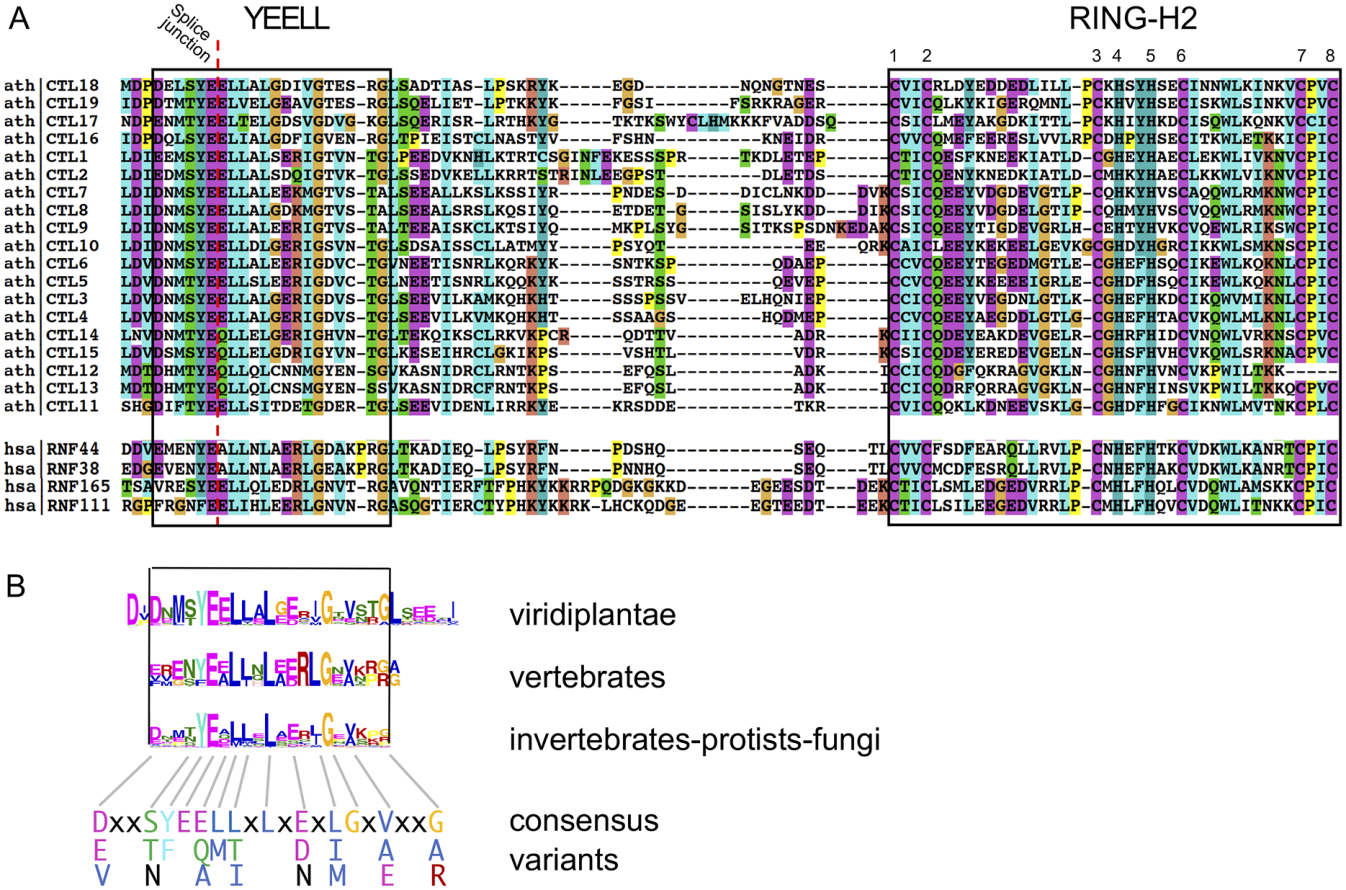
YEELL motifs and RING-H2 domains of *A*. *thaliana* and human CTL proteins. A. Alignment of the region encompassing YEELL motifs and RING-H2 domains is shown. The YEELL motif is enclosed in a rectangle and the residues involved in zinc ligation in the RING-H2 domain are numbered 1 to 8. The pointed red line across the YEELL sequence denotes a splice junction that is present in all the sequences. *A*. *thaliana* splice junction were obtained from Phytozome 10 (https://phytozome.jgi.doe.gov/pz/portal.html) and human splice junctions from Ensembl (http://www.ensembl.org/index.html). B. An alignment of three sequence LOGOs that include the YEELL motif obtained from 577 viridiplantae, 113 vertebrates and 61 invertebrates-protist-fungi CTL protein sequences is displayed and aligned with the YEELL motif from panel A. A twenty-one residues consensus sequence based on these three LOGOs is shown at the bottom; lower-case letter x denotes any amino acid.

Initially, CTL sequences were retrieved from *A*. *thaliana* and human proteomes. We readily identified 19 in *A*. *thaliana* and 4 in humans. We numbered the *A*. *thaliana* CTL from CTL1 to CTL19; CTL19 corresponded to BB, the previously described member of this family (See Fig 2A, and highlighted in yellow in S1 Table). The human CTL members were RNF44, RNF38, RNF165, and RNF111 (Fig 2A). Noteworthy, the spacing between the eight metal ligand residues is identical, except in two *A*. *thaliana* CTL sequences (see CTL10 and CTL16, Fig 2A). To identify and compare the YEELL motif in different taxa, sequence LOGOs were generated from the retrieved sequences (see analysis below). A comparison of sequence LOGOs generated from phylogenetically related groups displayed a similar sequence LOGO that includes the YEELL motif and the shared sequence extended the signature of YEELL to a conserved motif of 21 residues (D/E/V)XX(S/T/N)(Y/F)E(E/Q/A)(L/M)(L/T/I)XLX(E/D/N)X(L/I/M)GX(V/A/E)XX(G/A/R). (Fig 2B). Four out residues encompassing the YEELL conserved motif were conserved at taxonomic class level. For instance, a D residue at the begin and G at the end of the motif in viridiplantae, the R and L residues arranged consecutively in vertebrates and a G five residues before the end of the YEELL motif in either invertebrates, protists or fungi.

The similarities in sequence LOGOs provides support on the structure of the CTL family across eukaryotes defined in part by the YEELL motif located adjacent to a highly conserved RING-H2 domain. Indeed, the motif could adopt an α-helix as reveal by a secondary structure prediction performed on a variety of CTL proteins [20] (S1 Fig). Of note, when we compared gene architecture across several *CTL* genes (see analysis below), we noticed that a splice site within the YEELL motif was highly conserved among distant related species (see pointed red line in Fig 2A). This evolutionary conserved gene architectural feature supports the suggestion that the YEELL sequence is an evolutionary conserved motif.

### Identification of CTL proteins across eukaryotes

To gain insights into the evolutionary history of the CTL family from eukariotes, we surveyed proteomes across plants, animals, fungi, and protists. We performed BLAST searches using concatenated YEELL conserved motif with CTL RING-H2 domain from of *A*. *thaliana* and human orthologs (see Materials and methods). We retrieved sequences from all major phylogenetic groups.

**Fig 3:**
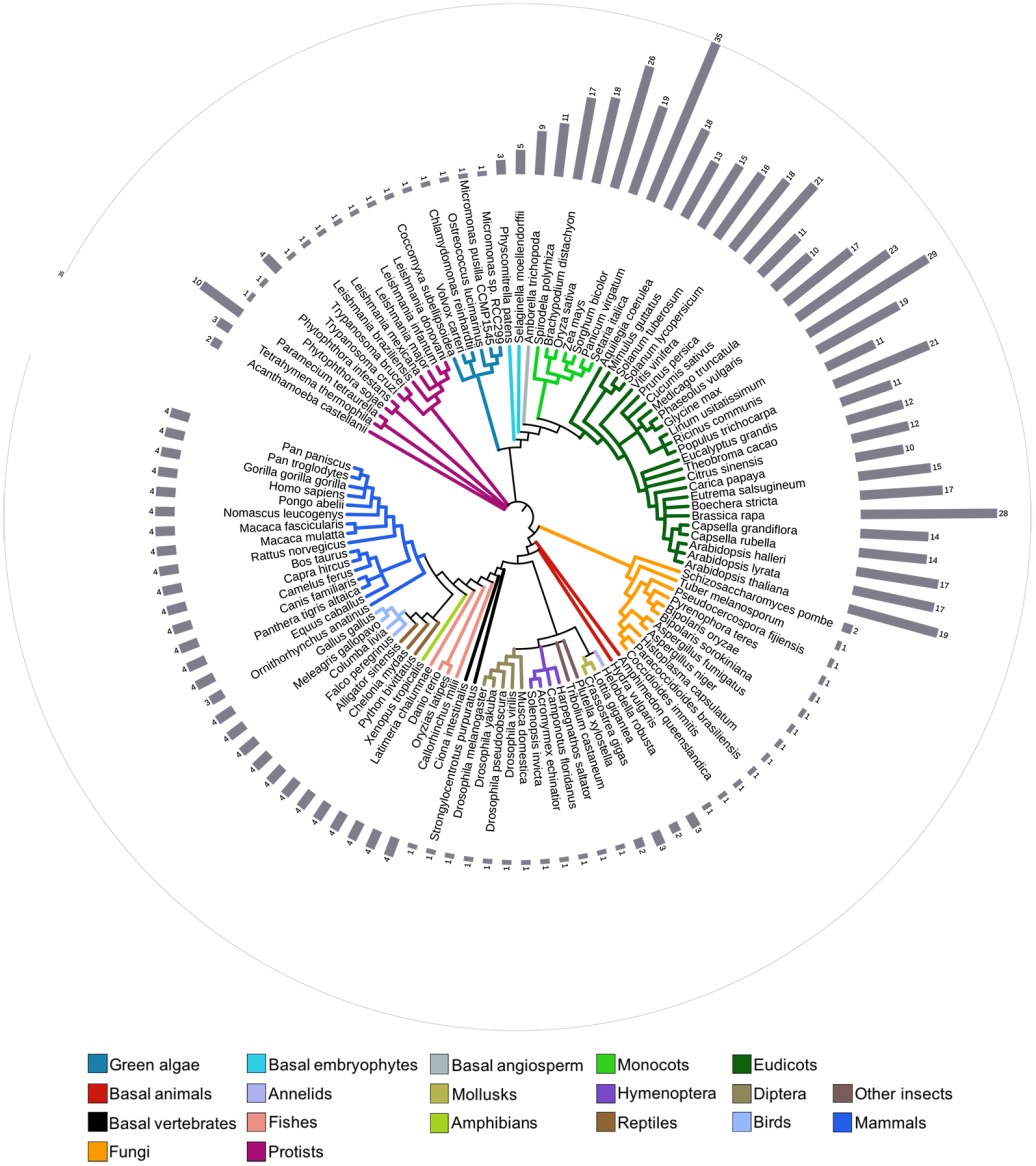
Number of retrieved CTL genes in eukaryotes. The phylogenetic relationship between forty-one viridiplantae (six green algae and thirty-five embryophyta), forty-six animal (twenty-eight vertebrates, two basal animals, two basal vertebrates, fourteen invertebrates), eighteen fungal, and fifteen protist genomes is displayed in a circle. Relationships were adapted from the National Center of Biotechnology Information (NCBI) taxonomy server (http://www.ncbi.nlm.nih.gov/Taxonomy). The color code for a selected group of organisms is displayed at the bottom. Lists and number of retrieved genes is shown in S1 Table.

The number of retrieved CTL sequences from animals and protists was much lower than the number of genes retrieved from plants, indicating that this family expanded in the plant lineage (Fig 3 and S1 Table). The animal genomes included two basal animals (the fresh-water polyp *Hydra vulgaris* and sponge *Amphimedon queenslandica*), two basal vertebrates (the sea squirt *Ciona intestinalis* and the purple sea urchin *Strongylocentrotus purpuratus*), twenty-eight vertebrates (sixteen mammals, four birds, three reptiles, one amphibian and four fishes), and fourteen invertebrates (seven diptera, four hymenoptera, two mollusks and one annelid). The genomes of vertebrates encoded four *CTL* genes, except for *Ornithorhynchus anatinus* that encoded three (Fig 3); while the basal vertebrates echinoderm (*Strongylocentrotus purpuratus*) and ascidians (*Ciona intestinalis*) encoded one. This data indicates that animal *CTL* genes may have evolved under strong evolutionary constraint on the CTL size family. Major groups of protist genomes were also surveyed. *CTL* genes were readily identified in Kinetoplasts (*Trypanosoma cruzi*, *Trypanosoma brucei*, *Leishmania major*), Oomycetes (*Phytophthora infestans*, *P*. *sojae*), Ciliates (*Paramecium tetraurelia*, *Tetrahymena thermophila*), and Acanthamoeba (*Acanthamoeba castellanii*). Nonetheless, *CTL* genes were not identified in Apicomplexans, which is a major group with several sequenced genomes (17 in the KEGG database). A single *CTL* gene was detected in most of the protist species (nine out of twelve). *Acanthamoeba castellanii* and *Trypanosoma cruzi*, had two and four *CTL* genes, respectively. Two ciliated genomes surveyed (*Tetrahymena thermophila* and *Paramecium tetraurelia*) had three and ten, respectively).

In a survey of fungi species, *CTL* genes were identified in Ascomycota but not in Basidiomycota. Of note, within the three Ascomycota subphyla, *CTLs* were identified in Pezizomycotina and Taphrinomycotina (Schizosaccharomycetes: *Schizosaccharomyces pombe*), but not in Saccharomycotina. CTL s were readily identified in two major classes of Pezizomycotina (Dothideomycetes and Eurotiomycetes) and Pezizomycetes (*Tuber melanosporum*), but were found neither in Sordariomycetes nor Leotiomycetes. Regarding the number of paralogs, a single *CTL* gene was identified in ten out of eleven fungi species; two *CTL* genes were present in *Schizosaccharomyces pombe*. Of note, in our survey of Basidiomycota, *CTL-like* genes were identified in several species. Despite, the expected sequence location for YEELL motif displayed certain degree of similitude between the phyla, but the YEELL motif is absent. It is noted that Basidiomycota sequences were not included in our analysis (an alignment of the sequences is displayed in S2 Fig).

The plant genomes surveyed consisted of six green algae (*Ostreococcus*. *lucimarinus*, *Micromonas pusilla* CCMP1545, *Micromonas* sp. RCC299, *Coccomyxa subellipsoidea*, *Chlamydomonas reinhardtii*, and *Volvox carteri*), two basal embryophytes (the moss *Physcomitrella patens*, the lycopod *Selaginella moellendorffii*), one basal angiosperm (the primitive magnoliophyta *Amborella trichopoda*), seven monocots, and twenty-five eudicot plants (see species in S1 Table). A single *CTL* was retrieved from each green algae, three in the moss and five in the lycopod. The number of *CTL* genes present in angiosperms ranged from nine in the primitive *A*. *trichopoda* to thirty-five in *Panicum virgatum*, thirty-one in *Glycine max* and twenty-eight in *Brassica rapa*, which underwent additional whole genome duplications events. Similar numbers of *CTL* genes occurred in species of close lineages. For instance, the *CTL* number in Brassicales ranged from 14 to 19 (except in *B*. *rapa* that contains 28) (see Fig 3). The high number of *CTL* members in the angiosperms tested indicates that there was greater expansion in the angiosperm lineage compared to other eukaryotic lineages.

### Deviation on the canonical CTL RING-H2-type domain sequence in distinct lineages

The comparison of the RING-H2 domain from ATL, BTL and CTL proteins in embryophytes showed conservation of the spacing between the eight metal ligands as well as the occurrence of a Pro (P) adjacent to the third metal ligand that suggests functional constraint (Fig 1). In plants, as exemplified in *A*. *thaliana* CTL proteins, 6 out of the 19 CTLs included the conserved proline (ath|CTL8, ath|CTL9, ath|CTL16, ath|CTL17, ath|CTL18 and ath|CTL19; see left panel of Fig 2A), suggesting that the position and occurrence of this proline is not constrained in plant CTL proteins. Conversely, four human paralogs contained the conserved proline adjacent to the third cysteine, suggesting that this proline exhibits structural constraint in vertebrates.

**Fig 4:**
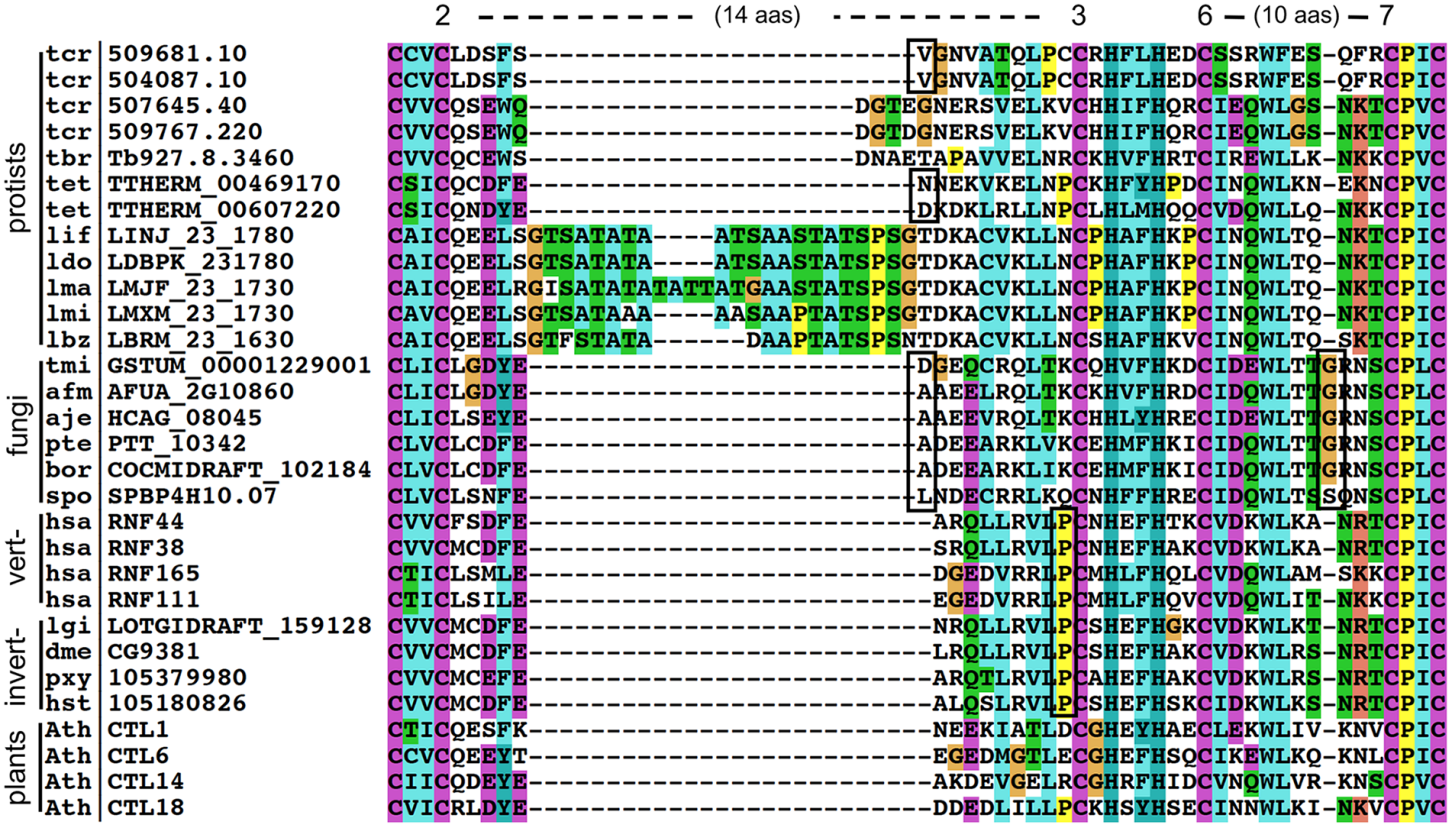
RING-H2 domain variations in CTL E3 ligase lineages. An alignment of representative proteins from protists, fungi, vertebrates, invertebrates and plants displaying the RING-H2 domain is displayed. ClustalX was used for sequence alignment and a default color code was applied. Numbers 2, 3, 6 and 7 indicate residues involved in zinc ligation and the distance between these residues is displayed in amino acid residues (aas). Single amino acid inserts are enclosed in a rectangle. The species abbreviations are listed in S1 Table.

In our analysis of the RING-H2 domain of retrieved CTL proteins across eukaryotes, we detected differences in the distance between the second and third metal ligand. This spacing in most of the RING-H2 domains in animals and plants was fourteen, as first inferred in plants. In some protists and fungi, this spacing showed variations that were lineage-specific. In Kinetoplasts, a group of protists from the phylum Euglenozoa including *T*. *brucei*, *T*. *cruzi* and five *Leishmania* species as well as two copies of the ciliate *T*. *thermophile* contained one to twenty-six residues more than the canonical distance (Fig 4). All fungal sequences retrieved displayed one additional residue between the second and third metal ligand as well as an additional residue between the six and seventh metal ligand.

### Phylogenetic distribution of CTLs across eukaryotes

**Fig 5:**
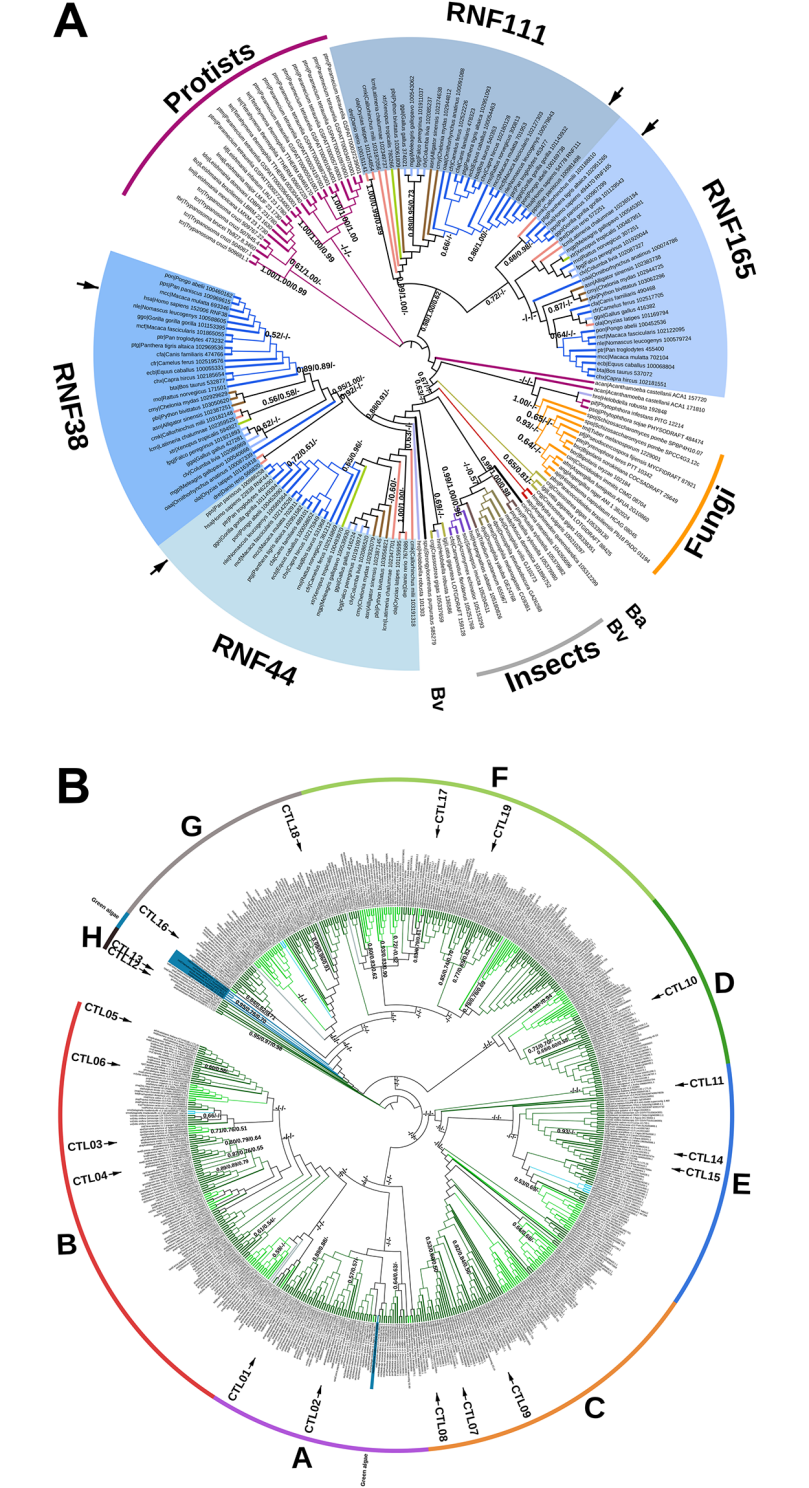
Phylogeny of CTL proteins. A. The tree was generated from complete protein sequences from animals, fungi and protists, and the topology of the tree obtained by the ML method. Statistical significance above 50% for NJ, and MP, and posterior probability above 0.5 for ML methods is indicated on the nodes (ML/NJ/MP). The generated from the RING-H2 domain sequences is presented in S2A Fig. The color code on the branches for organism groups is the same as in Fig 3. Human RNF38 and RNF44, RNF111/ARKADIA and RNF165/ARKL2 are indicated with arrows, and putative orthologs from each class are highlighted in shades of blue tones. B. Phylogeny of CTL proteins from plants. The tree was generated from RING-H2 domain sequences. The tree obtained by the ML method from complete protein sequences is displayed in S2B Fig. The color code on the branches is the same as found in Fig 3.

For the phylogenetic analysis of CTL proteins across eukaryotes, we designed two separated trees. One that contained 174 animal, protist and fungal sequences and the other contained 577 plant sequences. When all species were analyzed in the tree, most plants sequences grouped together but the support for branch classification was not strong (data not shown). We evaluated phylogenies generated with the Neighbor-Joining (NJ), the Maximum-Parsimony (MP) and the Maximum-Likelihood (ML) methods. The tree generated with complete animal, protist and fungal sequences is shown in Fig 5A, and the tree generated with the RING-H2 of viridiplantae is shown in Fig 5B. These two trees are representative phylogenies that were supported by high bootstrap values and showed suitable resolution of the species. The phylogenies using the RING-H2 domain of animal, protist and fungal sequences and the tree based on complete viridiplantae full-length protein sequences are displayed in S3 Fig.

The animal, protist and fungal trees were separated into two major branches that displayed taxonomic congruency. Vertebrates remained grouped in two clades, each one containing two classes of mammalian species. One containing putative orthologs of RNF38 and RNF44, and the other containing putative orthologs of RNF111/ARKADIA and RNF165/ARKL2 (highlighted in shades of blue tones, in Fig 5A). In the twenty-eight vertebrate species, one member was found for each one of the four groups; except for the putative RNF44 ortholog of *Ornithorhynchus anatinus* that was not found in data bases. Most of *CTL* genes, from basal animal and invertebrate as well as fungi species encode a single *CTL* member, suggesting that there are two independent sets of duplicated paralogs in vertebrates.

One of the ortholog from basal species for vertebrates *Strongylocentrotus purpuratus* 585279 was basal for the RNF38 and RNF44 and the other *Ciona intestinalis* 104265696 was basal for insect species, RNF38 and RNF44 as well (see Bv, Fig 5A). The two basal animals sequences, *Hydra vulgaris* 100210297 and *Amphimedon queenslandica* 105312299, were basal to vertebrates and invertebrates, including all insects and most mollusks (see Ba, Fig 5A). Among fungi, the eleven species grouped in a clade. Protists, Ciliates *(Tetrahymena thermophile* and *Paramecium tetraurelia*) and Kinetoplasts (two Trypanosoma species and four *Leishmania* species) grouped in two sister clades (Fig 5A). In the plant trees, green algae were positioned in external basal clades (*Ostreococcus lucimarinus* v2.0 38024, *Chlamydomonas reinhardtii* v5.5 Cre10.g442750.t1.2, *Volvox carteri* v2.1 Vocar20008863m, *Coccomyxa subellipsoidea* C-169 v2.0 60820 and *Micromonas* sp. RCC299 v3.0 61535). One inconsistency was the *Micromonas pusilla* CCMP1545 v3.0 50363 ortholog that was separated from the green algae sequences. In trees based on complete sequences, this afore mentioned ortholog did not group with green algae either (see S2B Fig). To ascertain the diversity of *CTL* genes in plants, groups of CTLs were built on collapsed branches. Seven CTL groups (A to G) were formed to include both monocot and eudicot orthologs, indicating that they diverged before the split of the two major classes of flowering plants. Few sequences that lacked phylogenetic congruence but found on external branches were organized into group H, including two *A*. *thaliana* paralogs, *ath|CTL12* and *ath|CTL13*. This grouping was assembled from the tree generated with the ML method based on the RING-H2 domain and each group contained at least one *A*. *thaliana* paralog. The distribution of CTL proteins from thirty-three angiosperm species found in the eight groups is listed in S2 Table. This tree showed coherence with trees generated by other methods or with complete sequences (shown in S3 Fig). The basal CTL proteins were distributed in two ortholog groups, placed in the group G (*P*. *patens* v3.3 Pp3c5_17300V3.1 and *S*. *moellendorffii* v1.0 SELMODRAFT_19194) and E (*P*. *patens* v3.3 Pp3c19_21510V3.1 and *P*. *patens* v3.3 Pp3c22_1990V3.1; and *S*. *moellendorffii* v1.0 SELMODRAFT 448412 and *S*. *moellendorffii* v1.0 SELMODRAFT 447294). Additional, a couple of CTLs from *S*. *moellendorffii* placed in the clade B (*S*. *moellendorffii* v1.0 SELMODRAFT 104716 and *S*. *moellendorffii* v1.0 SELMODRAFT 89607) (see blue colored branches in see Fig 5B).

### Overall domain architecture of CTL proteins

**Fig 6:**
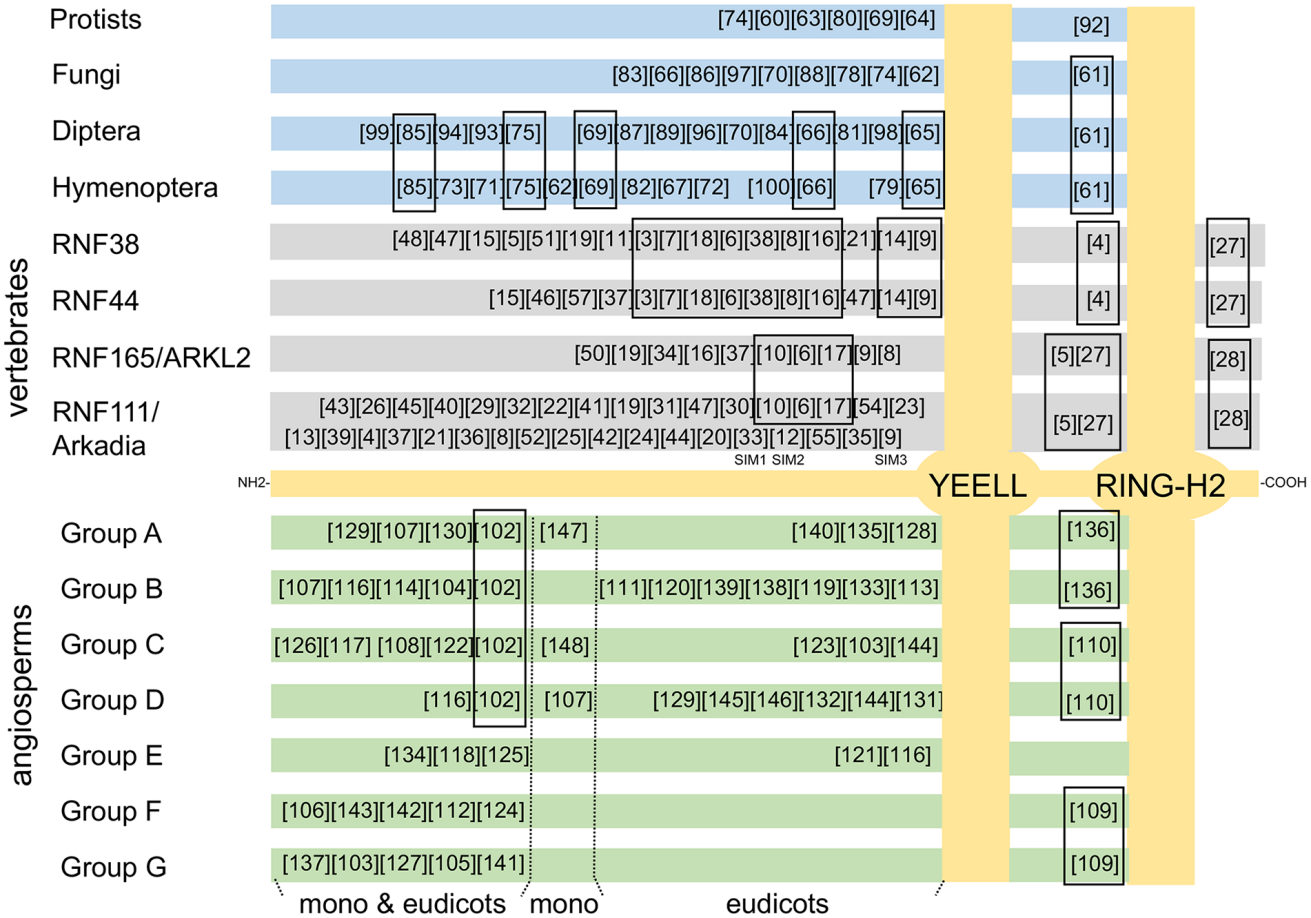
Distribution sequence LOGOs mapped to CTL proteins. A canonical CTL protein is represented in a pale gold color rectangle across the middle of the figure. The location of the YEELL motif and the RING-H2 domain are conventionally positioned in bubbles, crossing all over the CTL groups. Animal, fungi and protists protein groups are displayed above the canonical CTL (four vertebrate groups in gray rectangles; insects, diptera and hymenoptera; fungi and protists in blue rectangles), and seven angiosperm protein groups are below (green rectangles). Sequence LOGOs are represented by numbers in brackets and are sequentially arranged. Common LOGOs in adjacent groups are enclosed in rectangles. In angiosperm CTL proteins, the location of sequence LOGOs is divided in three: LOGOs present in both monocots and eudicots, LOGOs in monocots only and LOGOs in eudicots only. The catalog of sequence LOGOs is displayed in the S3 Table. LOGOs harboring SUMO-interaction motifs in RNF111/ARKADIA (SIM1, SIM2, SFIM3) are indicated.

We conducted MEME searches to assemble a domain architecture of the CTL proteins and to unravel conserved motifs that assisted in the characterization of this family. An initial search performed with the CTL-identified sequences to uncover which motifs were present in most CTL proteins rendered only common LOGOs encompassing the RING-H2 and the YEELL conserved motif (data not shown). We decided to perform individual searches with 111 vertebrate CTLs, 63 invertebrates, fungi and protists CTLs, and 577 plant CTLs. For each class, almost all of the LOGOs were mapped from the amino-terminus to the YEELL motif, one or two LOGOs between YEELL and the RING-H2 domain and two LOGOs after the RING-H2 (a summary of the mapped LOGOs is shown in Fig 6, a catalog of sequence LOGOs is displayed in S3 Table, and alignments of members of each group exposing the location of the sequence LOGOs are displayed on S4 and S5 Figs).

Analysis of invertebrates, fungi, and protists found that a LOGO mapped between YEELL and the RING-H2 domain is common to insects and fungi (see LOGO 61 in Fig 6 and S4 Fig), even though, a distinct domain architecture organization can be predicted for each class. Domain architecture organization is also distinct among insect orders (Diptera and Hymenoptera); however, they share about 21% of the LOGOs, including LOGO 61, 65, 66, 69, 75 and 85. Likewise, in vertebrates, discovered-LOGOs present in the CTL subfamilies RNF38, RNF44, RNF165/ARKL2 and RNF111/ARKADIA were mapped. For each of those subfamilies, a distinct architecture organization was obtained, and two distinct subgroups were evident based on sequence LOGOs organization; one comprising RNF44 and RNF38 orthologs (see LOGOs 3, 7, 18, 6, 38, 8, 16, 14, 9, 4 and 27, Fig 6 and S4 Fig) and the other RNF165/ARKL2 and RNF111/ARKADIA orthologs (see LOGOs 10, 6, 17, 5, 27 and 28, Fig 6 and S4 Fig). Near 75% of the shared-LOGOs between those subgroups were common to RNF44 and RNF38 orthologs whereas few were common to RNF165/ARKL2 and RNF111/ARKADIA; these two later RNF genes have been suggested to be partial paralogs [18]. It is noted that LOGOs with known motifs in vertebrates CTL RNF111/ARKADIA were detected, LOGOs 33, 12, 9 and 29 encompass motifs SIM1, SIM2 SIM3, and a histidine-rich region, respectively (Fig 6 and S4 Fig) [21].

Domain architecture further supports CTL classification from plants in eight groups based on the phylogenetic trees. Each group displayed a distinct domain architecture (Fig 6). The alignment of members from each group indicating the location of sequence LOGOs is given in S5 Fig. Except for group E, one sequence LOGO was mapped between YEELL and the RING-H2 domain, each of which was common to two groups and was present in both eudicot and monocot plants (see LOGOs 136, 110 and 109 in Fig 6). Likewise, about a half of the identified-LOGOs was mapped to both eudicot and monocot plants, suggesting that they are key components of ancestral motifs of the plant lineage. Moreover, unique LOGOs for the eudicot and monocot lineages were also uncovered (Fig 6).

### The incidence of spliceosomal introns in *CTL* genes

Architecture assessment on angiosperms *ATL* and *BTL genes* showed that spliceosomal introns within the coding DNA sequence (CDS) were absent in at least 90% of the genes [11]. Moreover, we previously described that introns at the 5′ untranslated region (UTR) of *BTL* genes were present in a half of the *A*. *thaliana* and *O*. *sativa* genes [22]. The 5’ UTR spliceosomal introns often include regulatory elements that contribute to gene expression or mRNA stability [23–25]. To recognize trends that may provide structural information on plant *CTL* genes, we looked for spliceosomal introns within the CDS and the 5’ UTR (see Table 1). In contrast with plant *ATL* and *BTL* genes, introns within the coding sequence were observed in plant *CTL* genes. An exception was the genes from two green algae species (*Micromonas pusilla* and *Ostreococcus lucimarinus*) and one of the *A*. *thaliana* genes (*ath|CTL16*). Indeed, the number of predicted introns in the green algae were fully assorted including one, seven and sixteen introns. Conversely, plant *CTL* genes included between three and six introns, with the exception of *ath|CTL17* which had eight. The fact that the number of introns is similar suggests that plant *CTL* genes maintained a similar gene structure. Retroposition is a mode of gene duplication that may explain the origin of intron-less *ATL* and *BTL* genes in the embryophyta lineage. In the plant *CTL* genes, whole genome duplication and other small scale duplication mechanisms are expected to account for CTL gene duplication.

**Table 1:** Incidence of spliceosomal introns in *CTL* genes.

Group	*CTL* gene	5’UTR intron(s) size (nt)	CDS intron (#)
Green algae	*cre|Cre10*.*g442750*.*t1*.*2*	no	7
	*csl|60820*	no	16
	*mpp|50363*	no	0
	*mis|61535*	no	1
	*olu|38024*	no	0
Basal	*ppp|Pp3c19_21510V3*.*1*	824 and 550	4
	*ppp|Pp3c22_1990V3*.*1*	1274 and 621	4
	*ppp|Pp3c5_17300V3*.*1*	545	6
A	*ath|CTL01*	235 and 472	4
	*ath|CTL02*	213 and 594	4
	*osa|Os05g48970 A*	4069	4
	*osa|Os01g47740 A*	4450	4
B	*ath|CTL03*	753 and 91	4
	*ath|CTL04*	593 and 105	4
	*ath|CTL05*	565	4
	*ath|CTL06*	1103	4
	*osa|Os04g55510*	1088	5
	*osa|Os04g10680*	816, 1116 and 619	4
C	*ath|CTL07*	566	4
	*ath|CTL08*	776	4
	*ath|CTL09*	721	4
	*osa|Os06g48040*	758	4
	*osa|Os02g05692*	1040	4
	*osa|Os04g51400*	1456	3
	*osa|Os08g14320*	no	3
D	*ath|CTL10*	2313	5
	*osa|Os05g07070*	4333	4
	*osa|Os01g06590*	2180	5
E	*ath|CTL11*	no	4
	*ath|CTL14*	no	4
	*ath|CTL15*	no	4
	*osa|Os01g49770*	594	4
	*osa|Os05g47670*	no	4
F	*ath|CTL17*	no	8
	*ath|CTL19*	82 and 337	4
	*osa|Os03g07790*	954	6
	*osa|Os09g35690*	749	4
	*osa|Os04g48260*	366	5
	*osa|Os06g03580*	327	6
G	*ath|CTL16*	no	no
	*ath|CTL18*	no	6
	*osa|Os08g43480*	102	4
	*osa|Os09g36460*	no	6
H	*ath|CTL12*	no	4
	*ath|CTL13*	no	3

In contrast to *ATL* genes and similarly to plant *BTL* genes, spliceosomal introns within the 5’ UTR were present in several plant *CTL* genes. These introns were detected in monocot and eudicot plants as well as in a basal species. 5’ UTR introns were not present in green algae indicating that they were acquired in embryophytes (Table 1). The 5’ UTR spliceosomal introns are often longer than introns within CDS. Indeed, introns of several kilobases are predicted in some *O*. *sativa CTL* genes (*osa|Os01g47740*, *osa|Os05g07070*, *osa|Os01g06590*, *osa|Os05g48970*) and *ath|CTL10* (see Table 1). The eight CTL groups supported by phylogenetic analysis contained members of both eudicots and monocots. Five of them included genes that contain at least one intron at the 5’ UTR, suggesting introns at the 5’ UTR in these groups were conserved by lineage.

### Potential protein-protein interactions mediated by a fragment containing the YEELL motif

**Fig 7:**
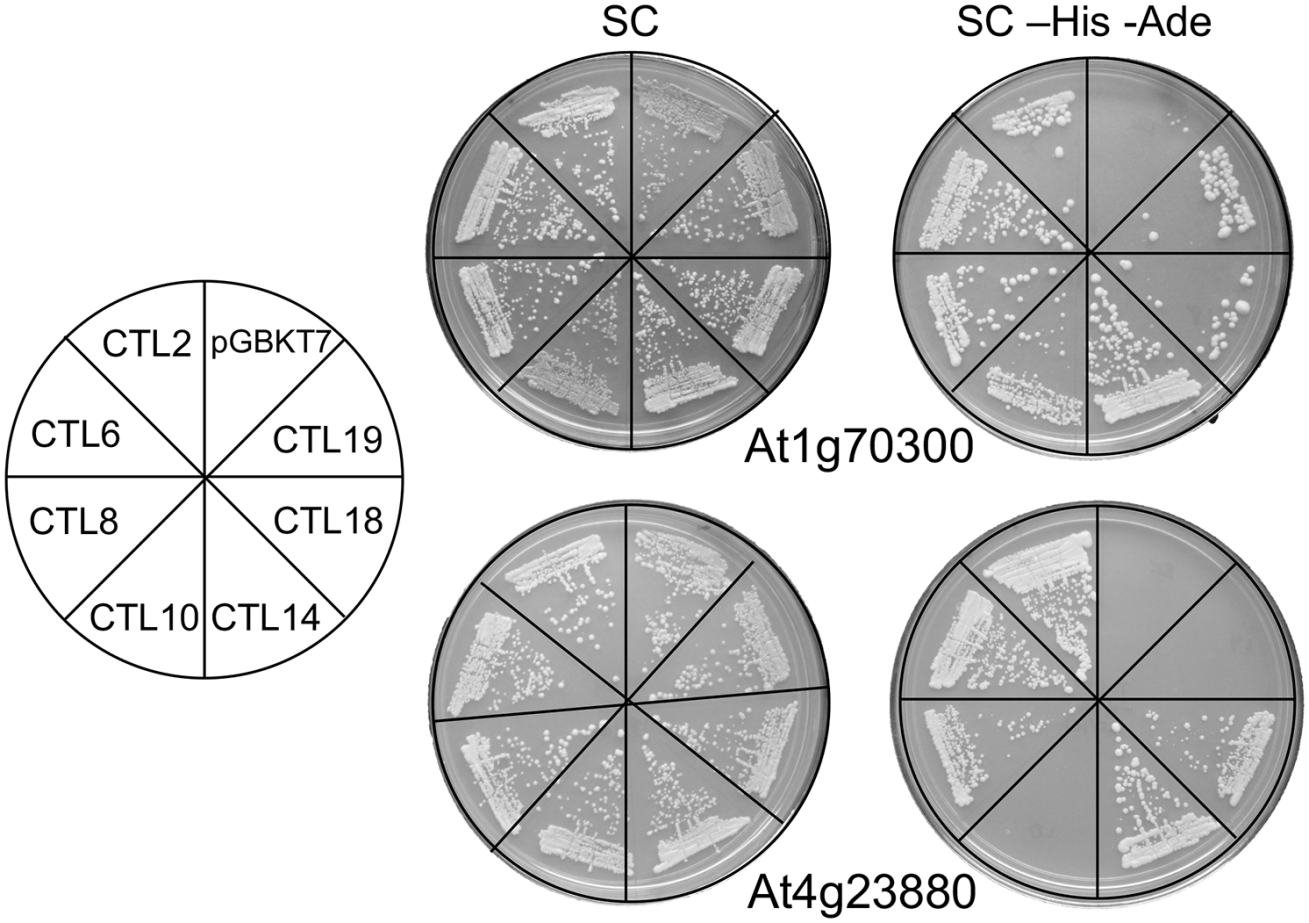
Interaction between At1g70300 and At4g23880 and YEELL-containing clones from seven *A*. *thaliana* CTL genes. Representative plates showing yeast two-hybrid interactions; the left panel shows the template of the plates. The yeast strain AH109 was cotransformed with pGBKT7 and pGADT7 derivatives, transformants were selected on SC lacking Trp and Leu and latter streaked onto SC medium lacking Trp and Leu (SC) and onto SC medium lacking Trp, Leu, His and Ade (high-stringency selection) (SC-His-Ade). The plates were placed in an incubator at 30°C for 96 hours; growth is regarded as thick streaks over background.

CTL proteins are likely be modular RING-H2 E3 ligases that contain domains for recognition of target proteins and a RING-H2 domain for the binding of the E2 ubiquitin-conjugating enzyme. We reasoned that the conserved YEELL motif may uncover a novel element that may support the function of *CTL* genes in general. Since defining regions that mediate protein–protein interactions are essential for analyzing E3 ligases, we look whether specific proteins might interact with the YEELL motif. We conducted yeast two-hybrid screens using as a bait a fragment encompassing the YEELL motif from ath|CTL2. We selected ath|CTL2, member of group A, as a representative of the family. From a set of positive clones obtained from the screen, two clones were recovered several times (see Materials and methods). Since we were inquiring whether YEELL motifs in plant CTLs were recognized by common proteins, we tested the interaction of these two clones with clones containing the YEELL motif of *CTL* genes belonging to seven different groups. One of the clones showed interaction with clones from all seven groups (see At1G70300 in Fig 7), and the other with clones from five groups (see At4g23880, in Fig 7). These results suggest that the YEELL motif can mediate protein-protein interactions and that in plants interactions may be common to all or several members of the family. The functional relevance of these interactions will be analyzed in future work.

## Discussion

In this study, we initiate the description of a multiple gene family of E3 ligases containing elements that are present in diverse eukaryotic species. We named this gene family CTL, in concurrence with the ATL and BTL families, our previously described RING-H2 E3 ligase families [11]. The basic features of the CTL family members are a highly related RING-H2 domain together with a novel motif, designed as YEELL, located close to the RING-H2 domain. The fact that the YEELL motif and the RING-H2 domain are found across eukaryotes indicates that they are probably evolutionarily related by homology and together may result in a specialized function. The occurrence of a unique sequence LOGO harboring the YEELL motif generated from all the retrieved sequences of varying organismal groups, supports the notion that *CTL* genes may have a common evolutionary origin. Moreover, the maintenance of a splice site within the YEELL motif strongly support this suggestion. More broadly, correlations between the co-occurrence of distinct motifs and specific RING-H2-type domains can be foreseen as follows: 1) transmembrane helices-GLD motif-ATL RING-H2-type domain, 2) BZF-GLD-like motif-BTL RING-H2-type domain, and 3) assorted domians-YEELL motif-CTL RING-H2-type domain.

RING fingers are Zinc Finger domains consisting of eight stringently spaced cysteine and histidine residues that coordinate two zinc ions in a unique cross-brace arrangement [26]. The overall arrangement of the globular conformation of the RING domain comprises a central α-helix along with loops of a varied length separated by beta-strands. Earlier comparison of two RING finger domain structures solved by NMR revealed substantial variations, indicating that the RING domain may be structurally diverse [27]. The structure of the RING finger domains from RNF111/ARKADIA, a CTL member and of EL5, an ATL member has been solved by NMR [28, 29]. These two RING domains have common features to several RING structures. For instance, the length of the α-helix is highly similar almost among them, and the carboxy-terminal region does not assume any secondary structure. In a structural comparison to seven RING finger domains, the RNF111/ARKADIA RING finger showed the highest resemblances to the ATL member EL5. This is expected since CTL and ATL RING finger domains are highly related RING-H2 domains.

Whole genome duplication and gene duplication on a small scale play important roles in the expansion and evolution of gene families [30]. The number of *CTL* genes identified in eukaryotic lineages was smaller than the number retrieved from angiosperms indicating that the CTL family underwent major gene expansion in angiosperms (33 plant genomes, see Fig 3). Looking at of the evolutionary history of the three E3 ligase families that encode a highly related RING-H2 domain (ATL, BTL, and CTL) suggests that *BTL* and *CTL* genes are present across eukaryotes while ATL genes are found only in plants [11]. Consistently, the extent of expansion of these ligases in the genome varied with the average number of *ATL* genes being higher than the average number of *BTL* or *CTL* genes (91 *ATL* genes compared to 14 *BTL* and 17 *CTL* genes). *CTL* like *BTL* genes are present across eukaryotes probably exerting essential functions, and thus substantial variations in gene dosage may be detrimental. It is conceivable that duplicated *CTL* genes, as assumed for *BTL* genes [31], are not easily retained in the genome after duplication.

Evolutionary expansion of *CTL* genes is also detected in vertebrates (twenty-eight genomes, including mammals, birds, reptiles, amphibians and fish), since basal animal and vertebrate, as well as most invertebrates, protists and fungal species, contain a single *CTL* gene. Yet strong evolutionary constraint to maintain four putative *CTL* paralogs in vertebrates is foreseeable. Two groups, each one with two classes of *CTL* paralogs, were found in vertebrates, suggesting that each group is the result of gene duplication events during animal evolution (see Figs 5A and 6). Indeed, one group consists of RNF111/ARKADIA and RNF165/ARKL2 paralogs that have been proposed to be paralogs [32]. Besides, few species encode more than one *CTL* paralog. Mollusks and annelids contain up to three paralogs and some protists up to ten paralogs. Increased number of *CTL* genes in some protists is expected, since several protists genomes groups encountered a number of rounds of whole genome duplications [33, 34].

Sequence LOGOs that mapped to the RING-H2 domain and to the YEELL motif were the only LOGOs generated across all eukaryotic organisms, suggesting a significant extent of innovation the *CTL* genes were subjected to during evolution. Such as acquiring new motifs by the RNF111/ARKADIA. RNF111/ARKADIA is a SUMO-targeted ubiquitin E3 ligase that specifically ubiquitinates previously sumoylated proteins [17]. RNF111/ARKADIA may be the result of assembling two adjacent genes in the vertebrate genome [32]. One gene, ARKADIA-Like 1 (ARKL1) features the carboxy-terminal RING-H2 domain, and the other gene, RNF165/ARKL2 features three SUMO-interaction motifs (SIM1, SIM2, SIM3; see Fig 6 and S4 Fig). Common sequence LOGOs are present in both RNF165/ARKL2, and RNF111/ARKADIA proteins (LOGOs 10, 6, 17, 9, 5, 27 28; see Fig 6), while a set of at least twenty-five sequence LOGOs make up a segment that is found in RNF111/ARKADIA (Fig 6). Accordingly, the large expansion of CTL in angiosperms correlates with an array of novel LOGOs. Although a unique domain architecture is predicted for each of the eight CTL groups, some LOGOs are common to more than one group. The location of these common LOGOs is mainly restricted to the region between the YEELL motif and RING-H2 domain (LOGO 136 in groups A and B, LOGO 110 in C and D, LOGO 109 in F and G) or adjacent to the YEELL motif (LOGO 102 in groups A, B, C and D). The region covering YEELL and the RING finger being evolutionarily conserved supports the idea that the YEELL-RING may be a central functional module and that the amino-terminus of CTL proteins is responsible for the divergence of this family.

Although, ATL, BTL, and CTL proteins encode very similar RING finger domains, their evolutionary history and gene architecture differs. When the gene architecture is analyzed, spliceosomal introns within the coding DNA are absent in nearly 90% of ATL genes, as well as in land plants BTL genes [22]. On the other hand, introns are present in most CTL genes across all eukaryotic lineages (data not shown), including land plants (see Table 1). The fact that *ATL* and *BTL* genes are intron-less may have enabled them to preserve their structure as basic modules. These modules include the GLD-RING-H2 component and a distinct amino-terminus element: hydrophobic helices in ATL proteins or BZF motif in BTL proteins (Fig 1A) [12, 31]. Significantly, a common amino-terminal domain for most CTL proteins was not detected. It is conceivable that the presence of spliceosomal introns at the amino-terminus region impact domain divergence and innovation of CTLs. Origin of new domains through gene duplication and exon shuffling events are important mechanisms that generate multidomain proteins during evolution [35–37].

Spliceosomal introns located at the 5’ UTRs were found in plant *CTL* genes too. This gene architectural feature may have been acquired early in CTL evolution as it is present in lineages comprising the basal *P*. *patens* as well as both monocot and eudicot species (Table 1). Spliceosomal introns located in the 5′ UTR of plant genes often encode regulatory elements that increase gene expression [23–25]. In *BTL* genes, 5’ UTR introns are common and exert effects on gene expression. It is likely that in plant *CTL* genes, 5’ UTR introns may be central to gene expression since they have been maintained during the evolution of this family.

Functional analysis of CTLs is in its initial stages. CTL proteins may consist of a basic module comprising the YEELL motif and the RING-H2 domain that have experienced expansion and diversification during evolution [12]. An intriguing issue is the function of the rather conserved YEELL motif as well as the GLD and GLD-like motifs. An earlier observation may point the direction for future work. We observed that the interaction between the RING-H2 domain from Ath|ATL10 and the E2 UBC11 was abolished in a yeast two-hybrid assay when the RING-H2 bait comprised the GLD motif. We speculated that GLD might affect the RING-H2 bait in a manner that hampers the assay, possibly by modifying the structure of the RING domain. Based on the fact that the YEELL motif also adjoins the RING domain, it is possible to assume that YEELL might exert an effect on the structure and/or function of the RING domain that might influence the crucial interaction between E2 and E3 enzymes. Considering the YEELL motif as part of an α-helix, it may work from the backside of the main α-helix of the RING domain. Supporting this assumption is the fact that the structure of two protein complexes (UbE2D2:RNF165:Ub and RNF38-UbcH5B-Ub) displayed one turn of an α-helix behind the RING domain [38, 39]. In the other hand, our results suggest that YEELL motif mediates protein-protein interactions (see Fig 7). Analysis of the two YEELL-interacting clones as well as the structural analysis of the RING-H2 domain associate with the YEELL motif will be valuable to unravel the function and the particular mechanism of action of this family of RING E3 ligases.

## Materials and methods

### Identification and sequence retrieval of putative *CTL* genes across eukaryotes

The sequences from animal, fungus, and protist genomes were retrieved from the Kyoto Encyclopedia of Genes and Genomes (KEGG) at http://www.genome.jp/kegg/. Other eukaryotic genomes used included two basal animals, two basal vertebrates, twenty-eight vertebrates, twelve protists, and eleven fungi. *CTL* genes were not identified in seventeen Apicomplexans from the genomes deposited in the KEGG database. The viridiplantae sequences used in this study were retrieved from six green algae, two basal embryophytes, seven monocots, and twenty-six eudicot genomes deposited in the Phytozome 10 database (https://phytozome.jgi.doe.gov/pz/portal.html#) (species are listed in S1 Table). Reiterated BLAST quests were accomplished using the polypeptide sequences containing the YEELL motif and the RING-H2 domain from the *A*. *thaliana* ath|CTL1 and ath|CTL19/BB orthologs, and from human ortholog RNF111. The *A*. *thaliana* genes were numbered *CTL1* to *CTL19*.

### Sequence alignments and phylogenetic analysis

Peptide sequence alignments used in the phylogenetic analysis were performed by MUSCLE version 3.8.31. ClustalX software version 2.0.12 20 was used to align and to decorate the alignments of sequences from organism groups [40]. To stablish the relationship among CTLs tree different method of phylogenetic reconstruction, Neighbor-joining (NJ), Maximum Parsimony (MP) trees and Maximum-likelihood (ML) were used and implemented in MEGA 7 [41]. To select reliable phylogenies with CTLs trees generated from complete polypeptide sequences were compared with trees based on the RING-H2 domain. As we have inferred before, the RING-H2 domain covers enough of these phylogenetically informative positions to resolve topologies with similar values rather than using complete protein sequences [12, 31]. Tree parameters were essentially performed as previously described [12, 31]. In general, trees exhibited resolution of the species without major branch classification variations. The phylogenies were valued to compare their consistency with conventional taxonomic classifications in order, family and genera obtained from the National Center of Biotechnology Information (NCBI) taxonomy server http://www.ncbi.nlm.nih.gov/Taxonomy. The tree phylogenies were displayed and edited by the Interactive Tree Of Life (iTOL) v3 at http://itol.embl.de/ [42]. We chose a color code as described in Fig 3.

### Generation of sequence LOGOs

To search for conserved motifs in CTL proteins, we used Multiple EM for Motif Elicitation (MEME) implemented in MEME software (University of Queensland, St. Lucia, Australia) version 4.11.3 (http://meme-suite.org/). The following parameters were used: values of 0 or 1 per sequence, and 6 and 75 amino acids as minimum and maximum sizes of motifs, respectively; the E-value cutoff was < e^-10^. Sixty non-redundant sequence LOGOs from vertebrates, 40 from invertebrates, fungi and protists and 50 from plants that ranged from 6 to 50 amino acids were collected (see Fig 6 and S3 Table). All three searches contained the sequence LOGOs encompassing the RING-H2 domain and a single sequence LOGO with the YEELL motif. In our study, we considered the RING-H2 to include the sequence with cysteines that flank the domain and the YEELL motif to include highly related LOGOs generated in the three independent searches (see LOGOs in Fig 2B).

### Yeast two-hybrid screenings and assays

A fragment encoding 114 amino acid residues of ath|CTL2 encompassing the YEELL motif without the RING-H2 domain was amplified by PCR from *A*. *thaliana* genomic DNA and cloned into the pGBKT7 plasmid (Clontech, Mountain View, CA).

An *A*. *thaliana* Matchmaker cDNA library in plasmid pGAD10 (Clontech, Mountain View, CA) was screened in the assay. Transformants were obtained in the *Saccharomyces cerevisiae* strain AH109, selected on SC lacking Trp and Leu, and then screened under high-stringency selective conditions (medium lacking Trp, Leu, His, and Ade). To examine whether YEELL motifs from other *A*. *thaliana CTL* genes may function in a similar way, fragments encompassing the YEELL region from CTL groups B to G (ath|CTL6, ath|CTL8, ath|CTL10, ath|CTL14, ath|CTL18 and ath|CTL19) were amplified by PCR from *A*. *thaliana* genomic DNA and then cloned into the pGBKT7 plasmid (see S4 Table). Then, they were cotransformed into AH109 with two clones that were recovered several times from the screen: At4g23880, hypothetical protein (recovered 21 times) and At1g70300, K^+^ UPTAKE PERMEASE 6, KUP6, potassium transporter (recovered 6 times). Cotransformants combinations were tested under high-stringency selective conditions.

## Supporting information

S1 Fig Prediction of secondary structures of CTL proteins by the Chou-Fasman method. Prediction of α-helix in thirty CTL proteins from twelve evolutionary diverse species was performed by the Chou-Fasman method in the Protean module (protein structure analysis & prediction) of Lasergene (https://www.dnastar.com). α-helix prediction is shown by boxes; green boxes include YEELL sequences and red boxes RING-H2 sequences.
(TIFF)

S2 Fig Protein sequence alignment of fungal CTL and CTL-like proteins. The sequence alignments were performed using ClustalX 2.0.12; default colors were used. A sequence LOGO encompassing the YEELL motif is shown at the top. The region encompassing the YEELL sequence in Basidiomycetes is enclosed in a rectangle. Abbreviations are as follows: Dothide, Dothideomycetes; Pe, Pezizomycotina; Eurotio, Eurotiomycetes; Sch, Schizosaccharomycetes; Basidio, Basidiomycetes. The species abbreviations are listed in S1 Table.
(TIF)

S3 Fig A. Phylogenetic trees of animal, fungi and protist CTL and of plant CTL sequences based on RING-H2 by the ML. B. Plant phylogeny by the ML method based on complete protein sequences. The topology was generated by the ML method; statistical significance above 50% for NJ, and MP, and posterior probability above 0.5 for ML methods is indicated on the nodes (ML/NJ/MP).
(PDF)

S4 Fig Protein alignments of protists, fungi, invertebrates, and vertebrates (RNF38, RNF44, RNF165 and RNF111) CTL sequences. The sequence alignments were performed using ClustalX 2.0.12; default colors were used. Protein sequences with large insertion or deletions were not included in the alignment. Regions encompassing the YEELL motif and the RING-H2 are enclosed in red rectangles; sequence LOGOs are enclosed in black rectangles. In the RNF111 alignment, LOGOs harboring SUMO-interaction motifs in RNF111/ARKADIA (SIM1, SIM2, SIM3) are indicated as well as previously described regions (polyhistidine, ARKMN, ARKMC) [21].
(PDF)

S5 Fig Protein alignment of plant CTL proteins. The sequence alignments were performed as described in S4 Fig.
(PDF)

S1 Table Lists and number of retrieved genes from animals, fungi, protists, and plants. (PDF)

S2 Table Distribution of CTL proteins retrieved from 33 angiosperm species in 8 groups. (PDF)

S3 Table Catalog of sequence LOGOs generated from vertebrates, invertebrates, fungi, protists, and plants. (PDF)

S4 Table List of oligonucleotide used to clone ath|CTLs into pGBK-T7. (PDF)
