# Supplementary figures and images for: CTLs, a new class of RING-H2 ubiquitin ligases uncovered by YEELL, a motif close to the RING domain that is present across eukaryotes
Source: PLoS One. 2018 Jan 11;13(1):e0190969. doi: 10.1371/journal.pone.0190969 (PMC5764321; doi:10.1371/journal.pone.0190969)

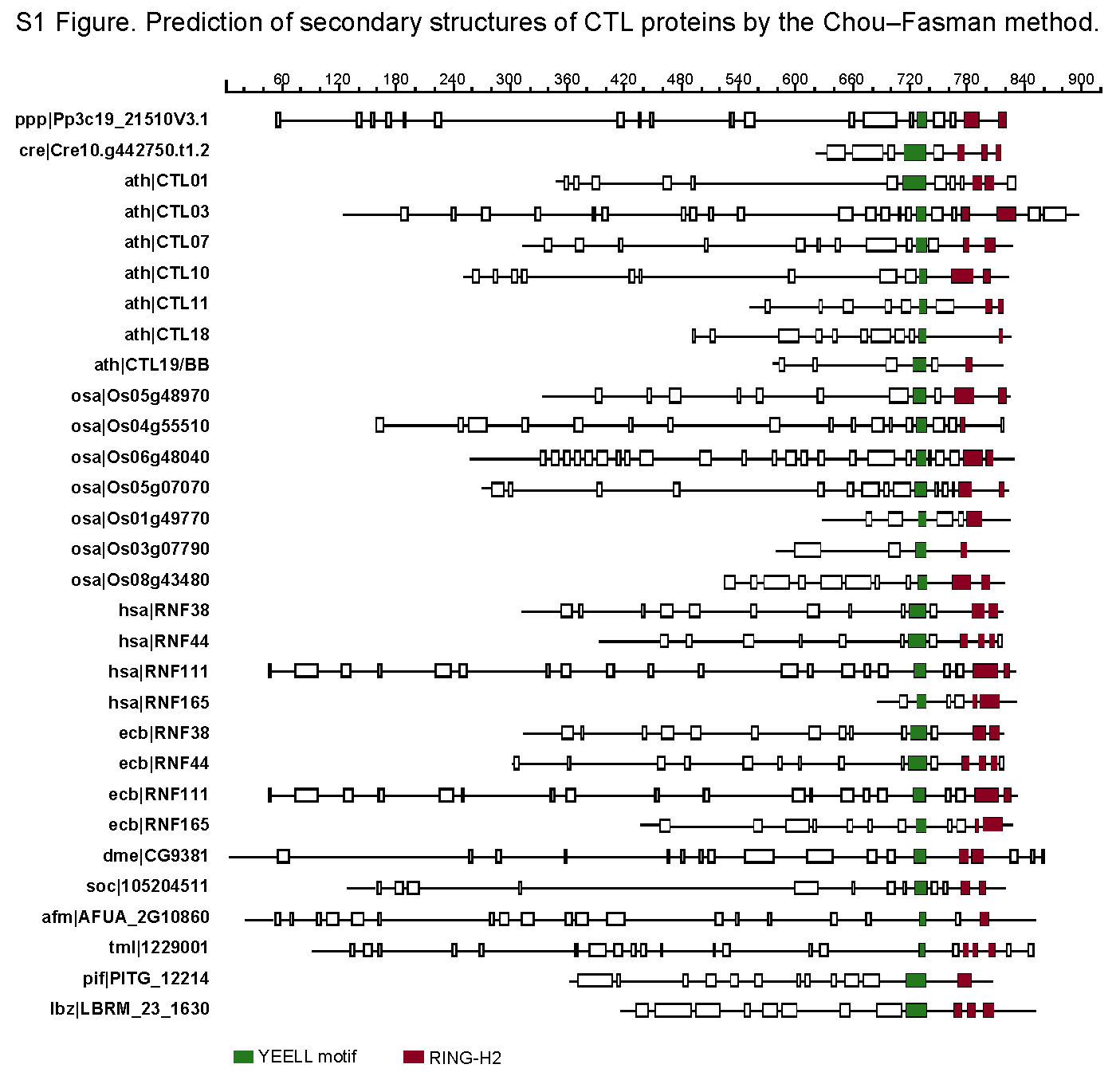

Supplement: S1 Fig — Prediction of α-helix in thirty CTL proteins from twelve evolutionary diverse species was performed by the Chou-Fasman method in the Protean module (protein structure analysis & prediction) of Lasergene (https://www.dnastar.com). α-helix prediction is shown by boxes; green boxes include YEELL sequences and red boxes RING-H2 sequences. (TIFF) [file pone.0190969.s001.tiff]

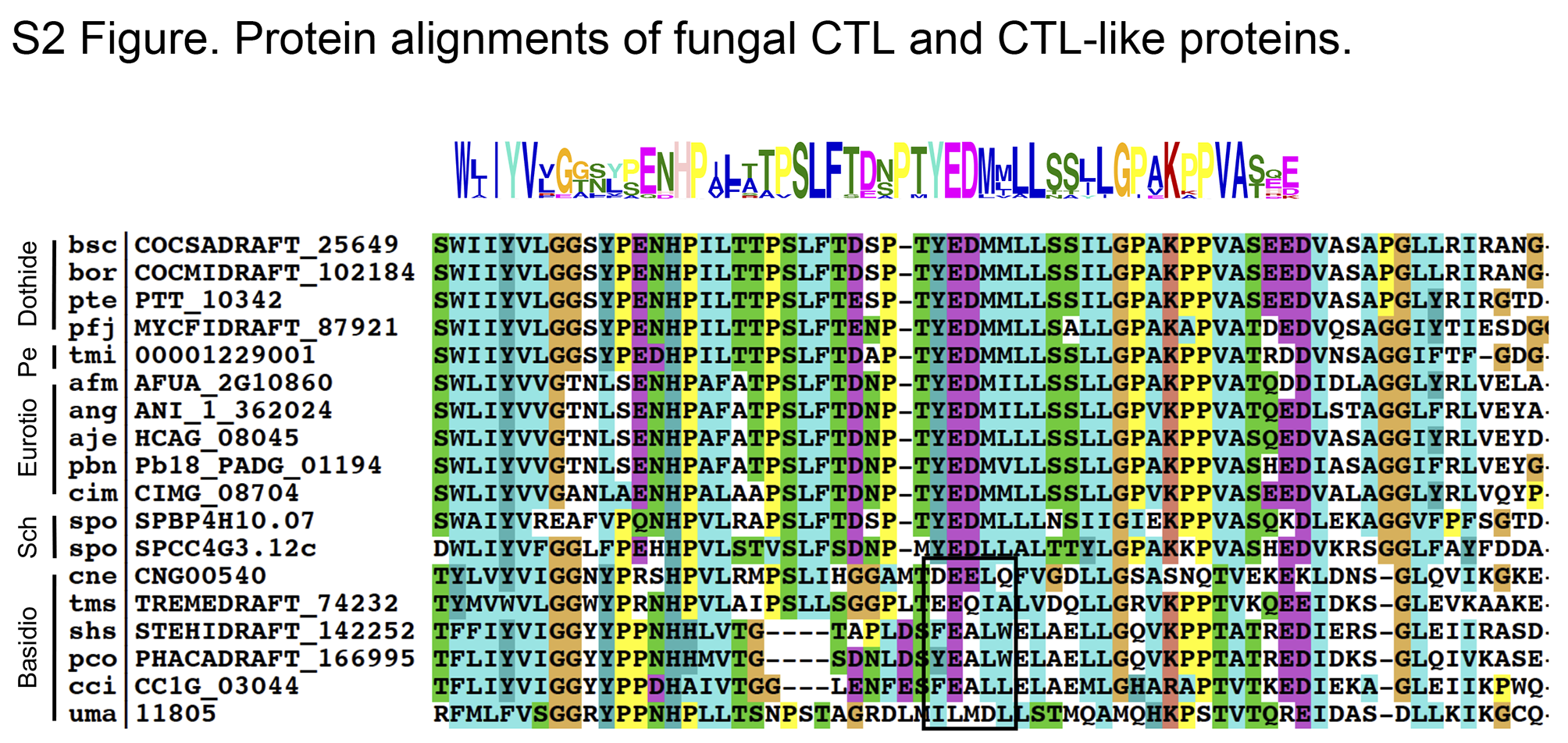

Supplement: S2 Fig — The sequence alignments were performed using ClustalX 2.0.12; default colors were used. A sequence LOGO encompassing the YEELL motif is shown at the top. The region encompassing the YEELL sequence in Basidiomycetes is enclosed in a rectangle. Abbreviations are as follows: Dothide, Dothideomycetes; Pe, Pezizomycotina; Eurotio, Eurotiomycetes; Sch, Schizosaccharomycetes; Basidio, Basidiomycetes. The species abbreviations are listed in S1 Table. (TIF) [file pone.0190969.s002.tif]

# A

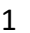

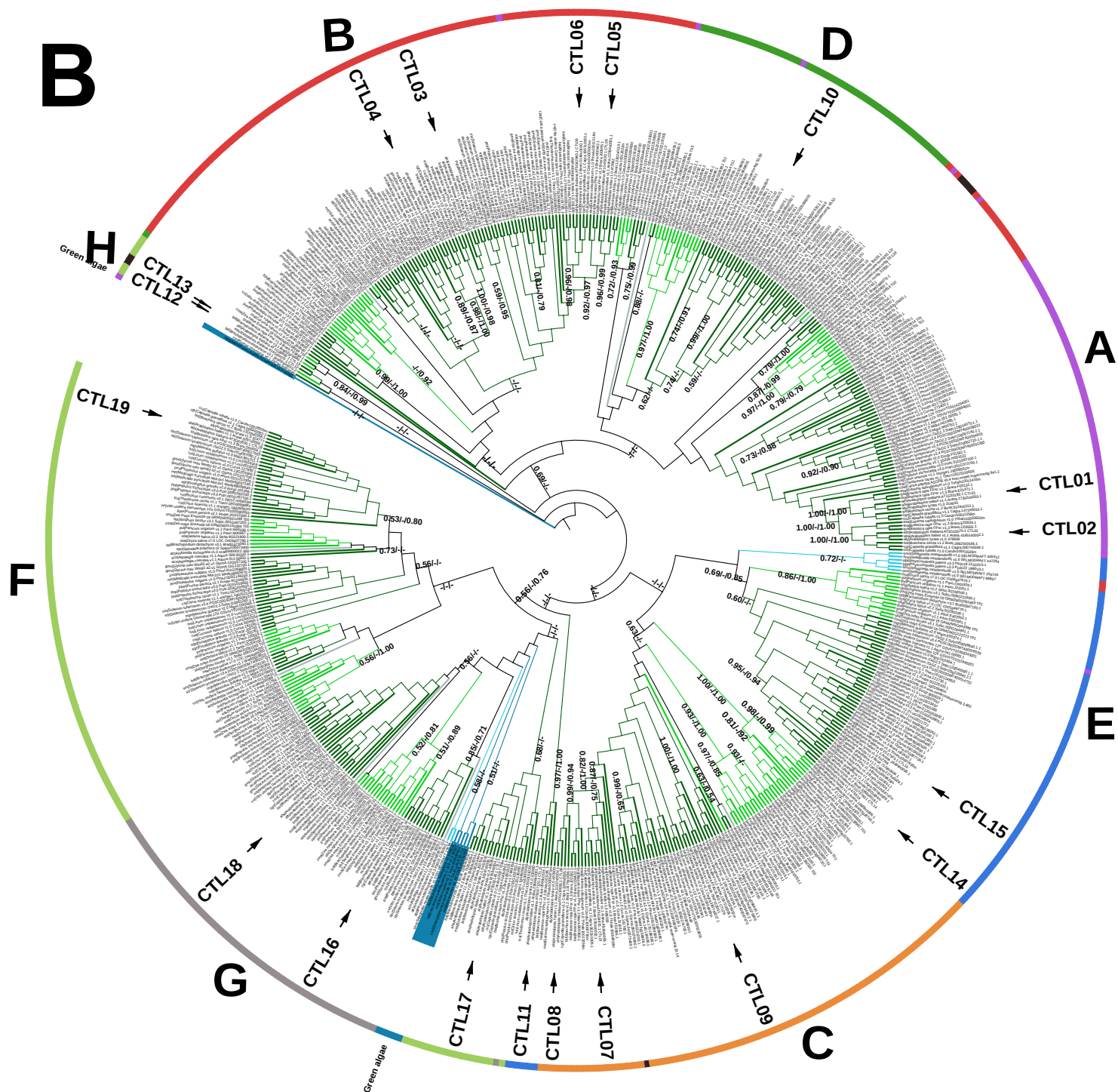

Supplement: S3 Fig — A. Phylogenetic trees of animal, fungi and protist CTL and of plant CTL sequences based on RING-H2 by the ML. B. Plant phylogeny by the ML method based on complete protein sequences. The topology was generated by the ML method; statistical significance above 50% for NJ, and MP, and posterior probability above 0.5 for ML methods is indicated on the nodes (ML/NJ/MP). (PDF) [file pone.0190969.s003.pdf]
