# Supplementary material for: CTLs, a new class of RING-H2 ubiquitin ligases uncovered by YEELL, a motif close to the RING domain that is present across eukaryotes
Source: PLoS One. 2018 Jan 11;13(1):e0190969. doi: 10.1371/journal.pone.0190969 (PMC5764321; doi:10.1371/journal.pone.0190969)
Supplement: S4 Fig — The sequence alignments were performed using ClustalX 2.0.12; default colors were used. Protein sequences with large insertion or deletions were not included in the alignment. Regions encompassing the YEELL motif and the RING-H2 are enclosed in red rectangles; sequence LOGOs are enclosed in black rectangles. In the RNF111 alignment, LOGOs harboring SUMO-interaction motifs in RNF111/ARKADIA (SIM1, SIM2, SIM3) are indicated as well as previously described regions (polyhistidine, ARKMN, ARKMC) [21]. (PDF) [file pone.0190969.s004.pdf]

S4 Figure. Protein alignments of protists, fungi, invertebrates and vertebrates (RNF38, RNF44, RNF165 and RNF111) CTLs.

Protists

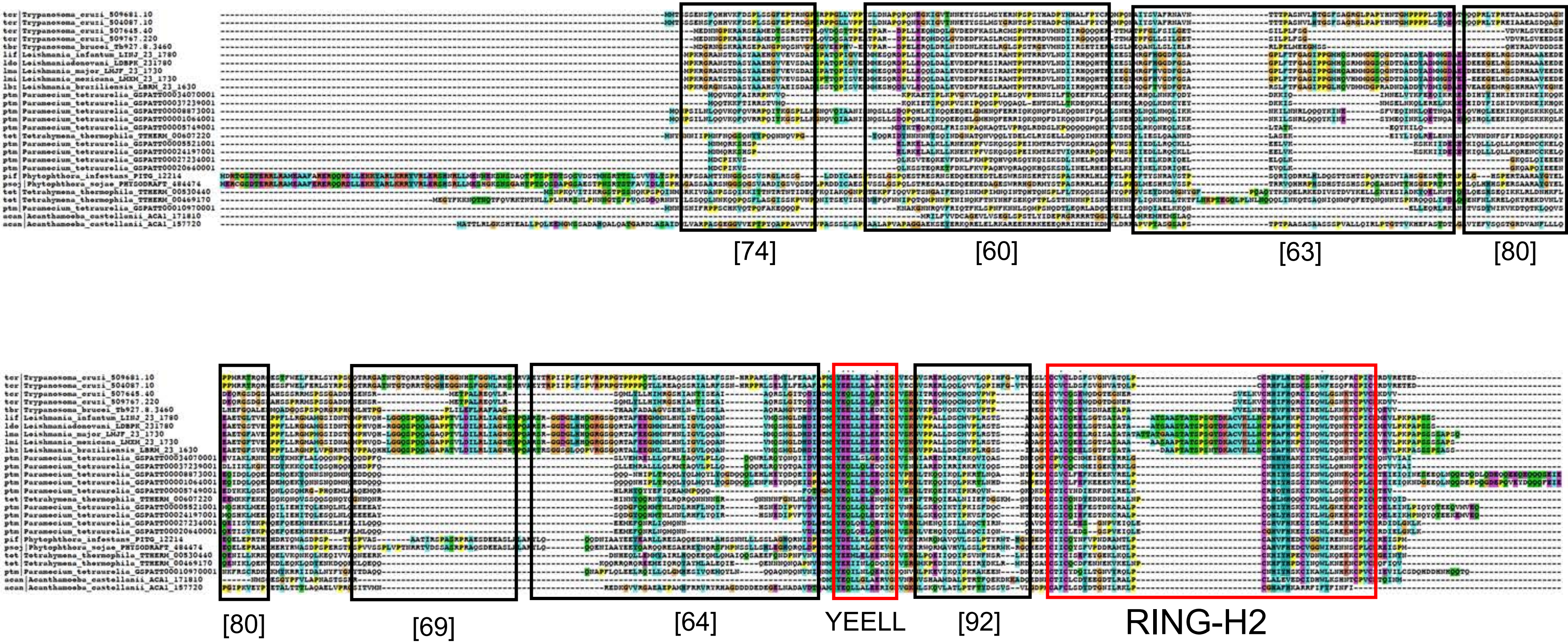

Fungi

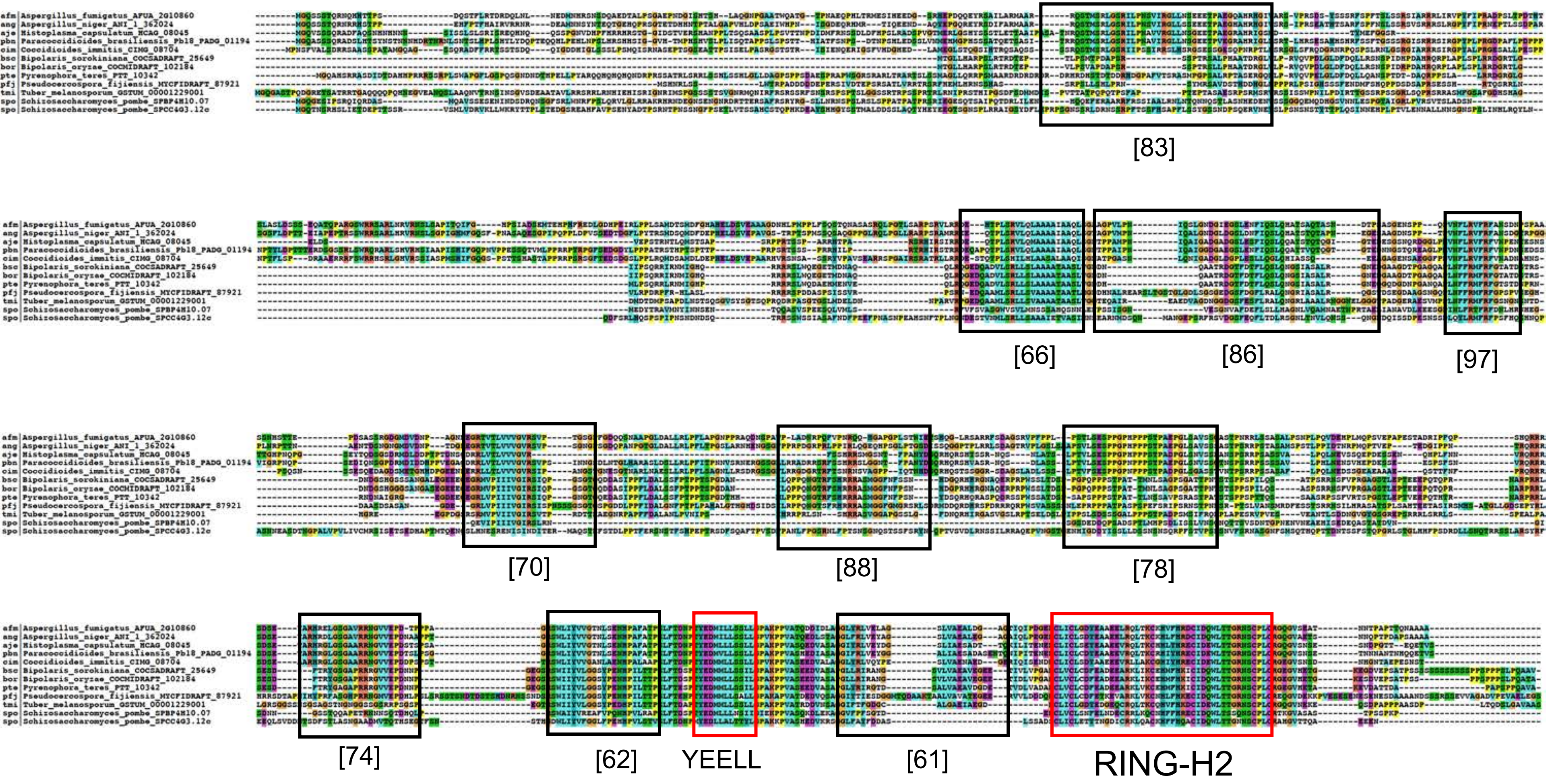
afm Aspergillus_fumigatus_AFUA_2G10860
ang Aspergillus_niger_ANI_1_362024
aje Histoplasma_capsulatum_HCAG_08045
pbn Paracoccidioides_brasiliensis_Pb18_PADG_01194
cim Coccidioides_immitis_CIMG_08704
bsc Bipolaris_sorokiniana_COCSADRAFT_25649
bor Bipolaris_oryzae_COCMIDRAFT_102184
pte Pyrenophora_teres_PTT_10342
pfj Pseudocercospora_fijiensis_MYCFIDRAFT_87921
tmi Tuber_melanosporum_GSTUM_00001229001
spo Schizosaccharomyces_pombe_SPBP4H10.07
spo Schizosaccharomyces_pombe_SPCC4G3.12c
[83]
[66]
[86]
[97]
[70]
[88]
[78]
[74]
[62]
YEELL
[61]
RING-H2

Invertebrates

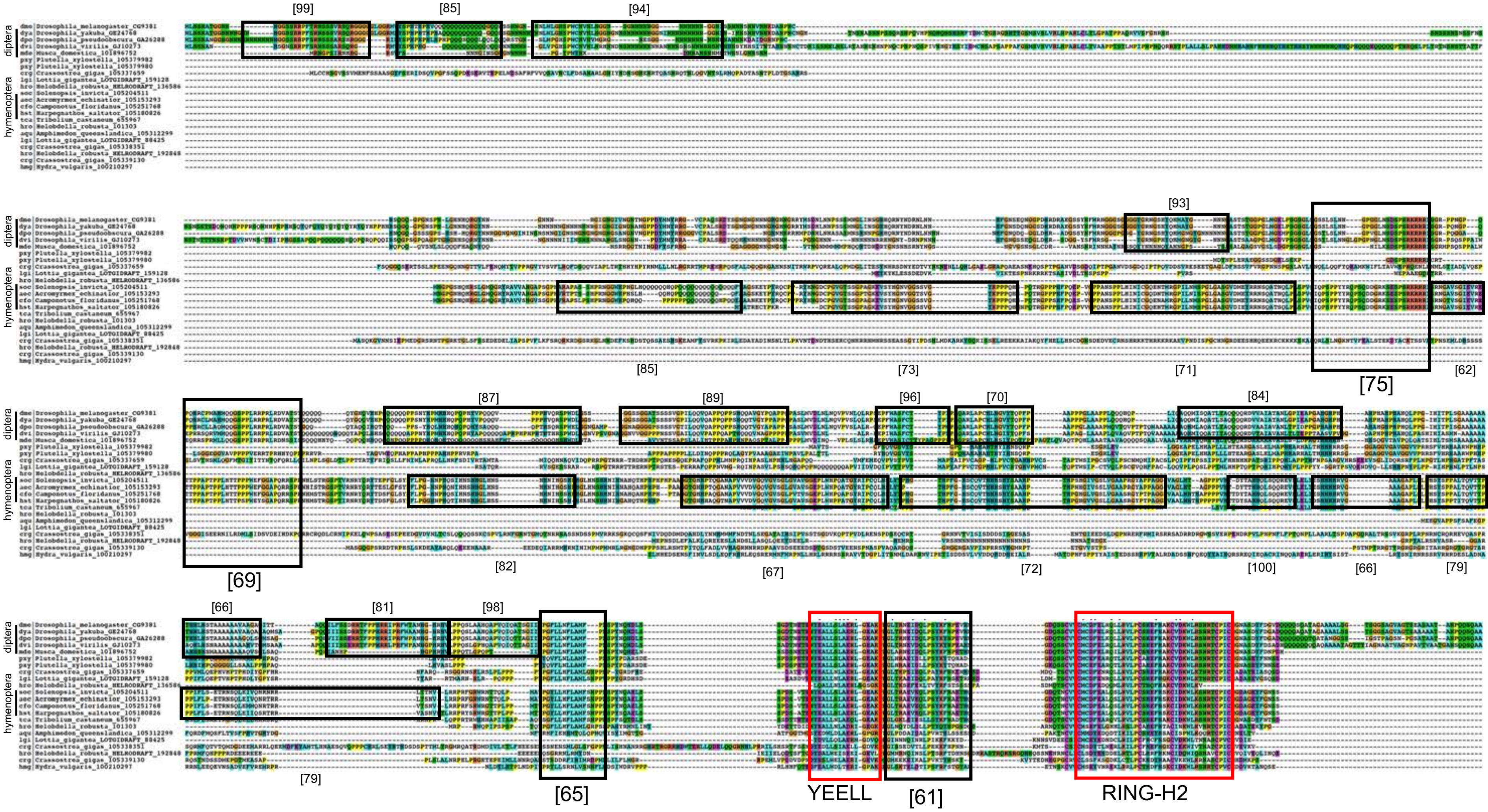

RNF38

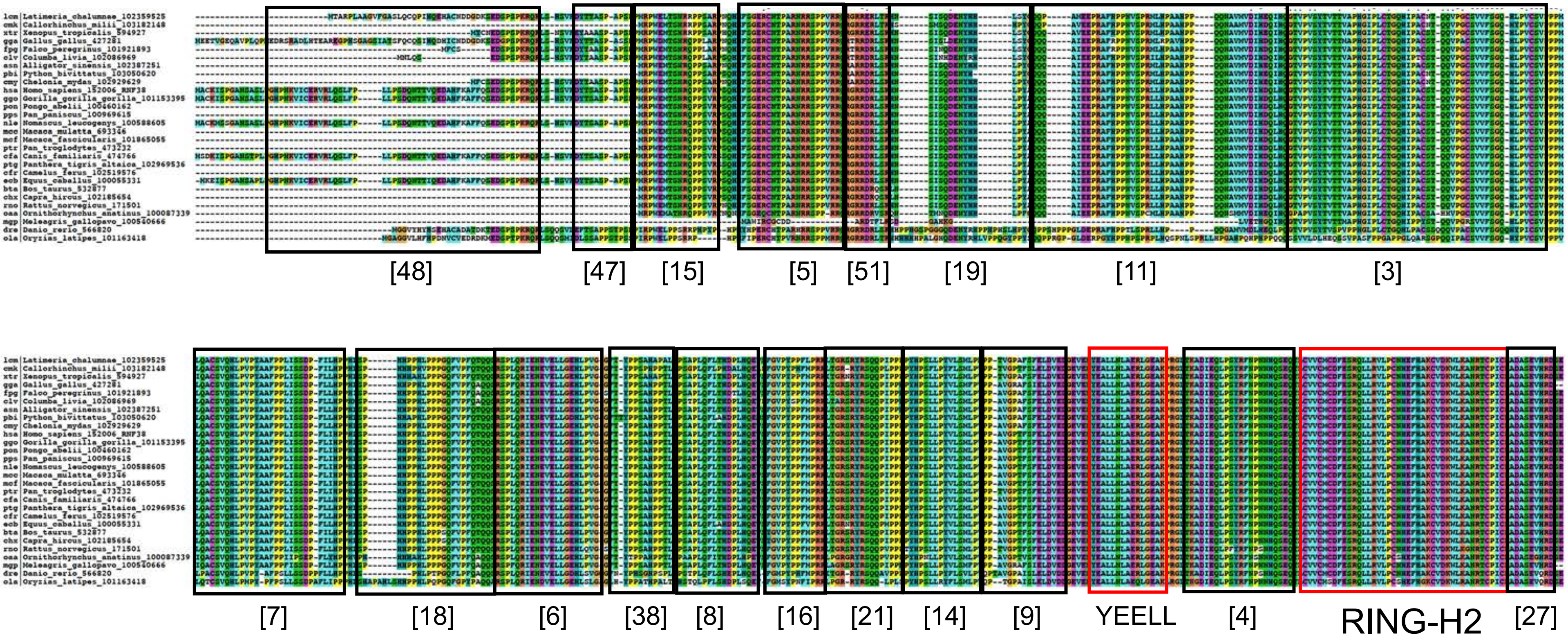

RNF44

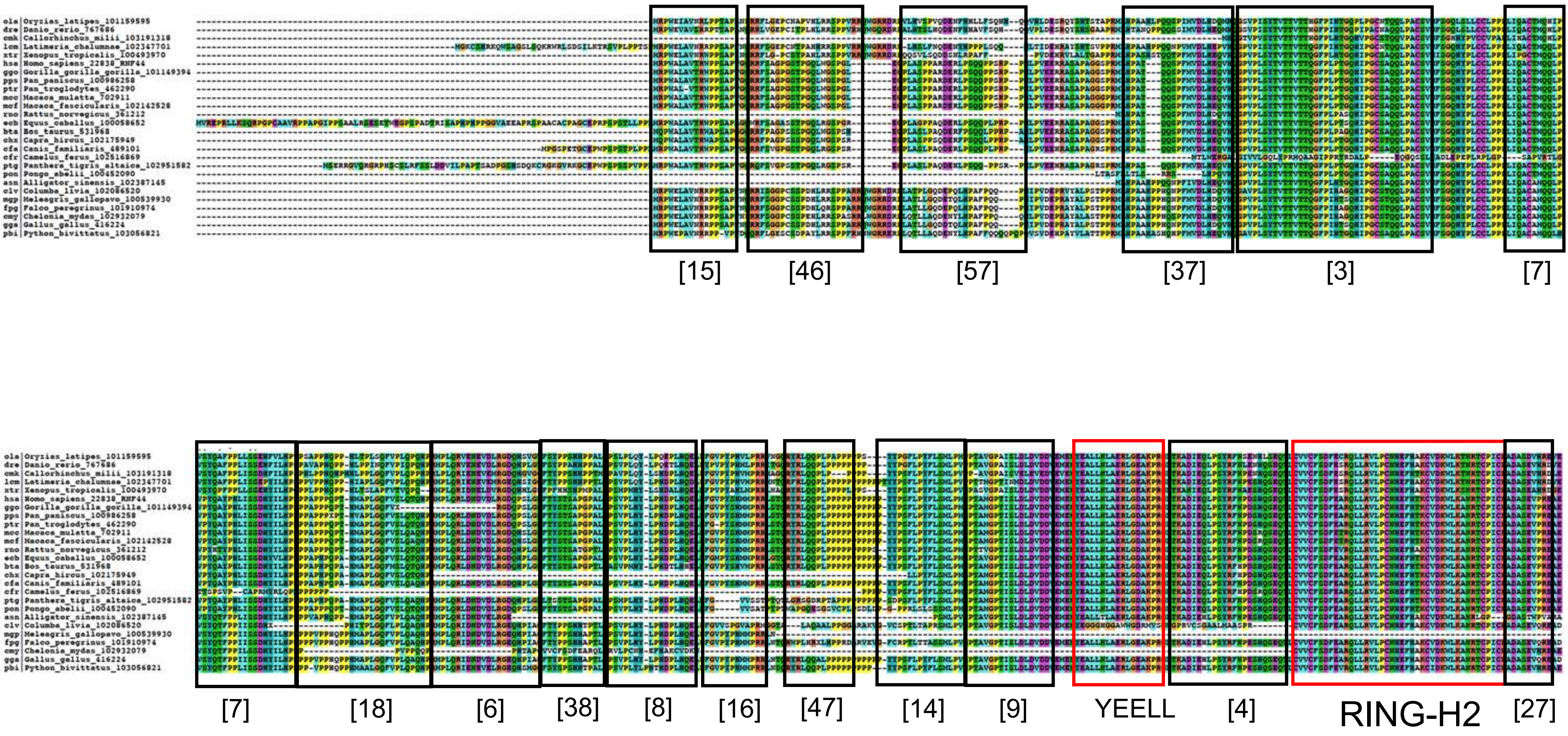

RNF165

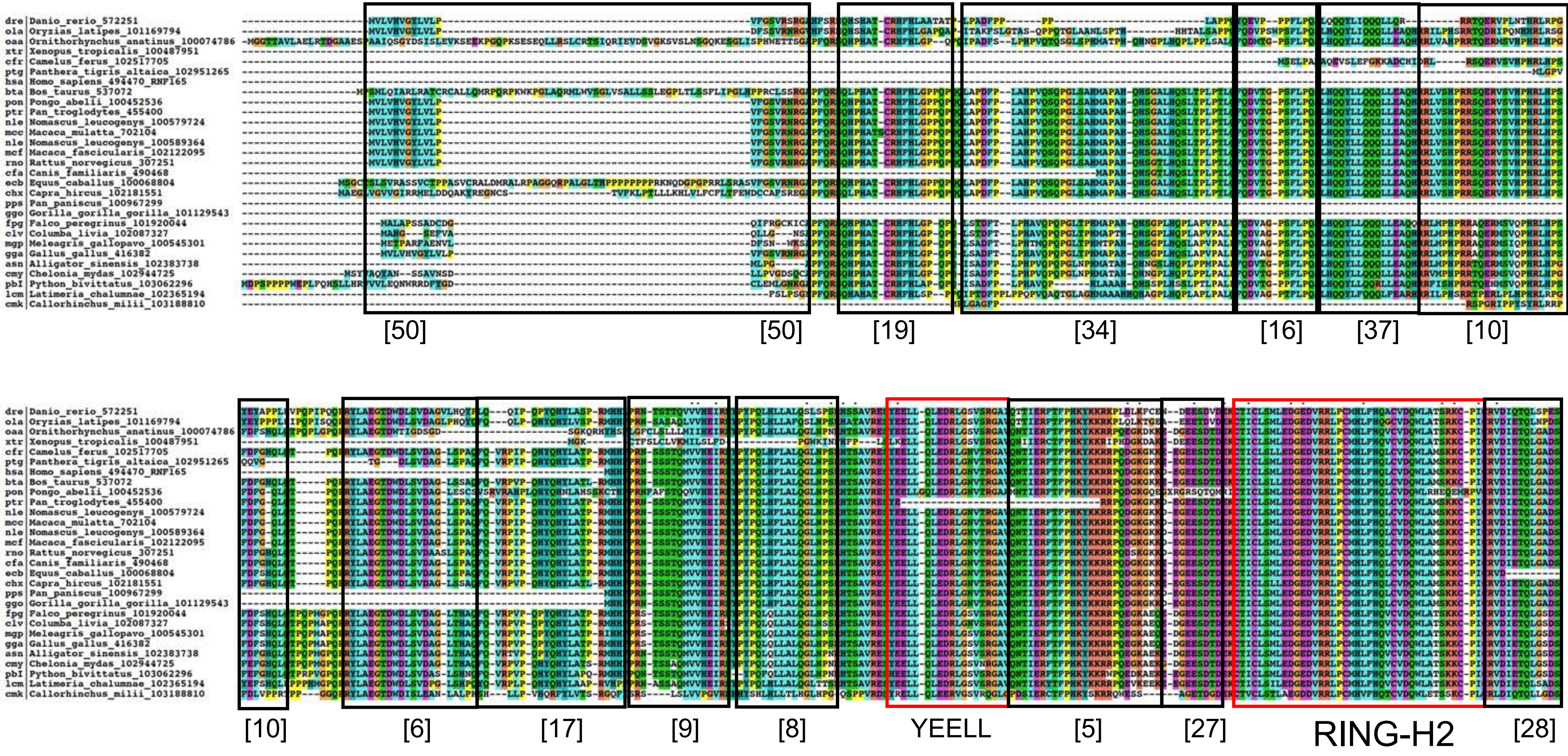

RNF111

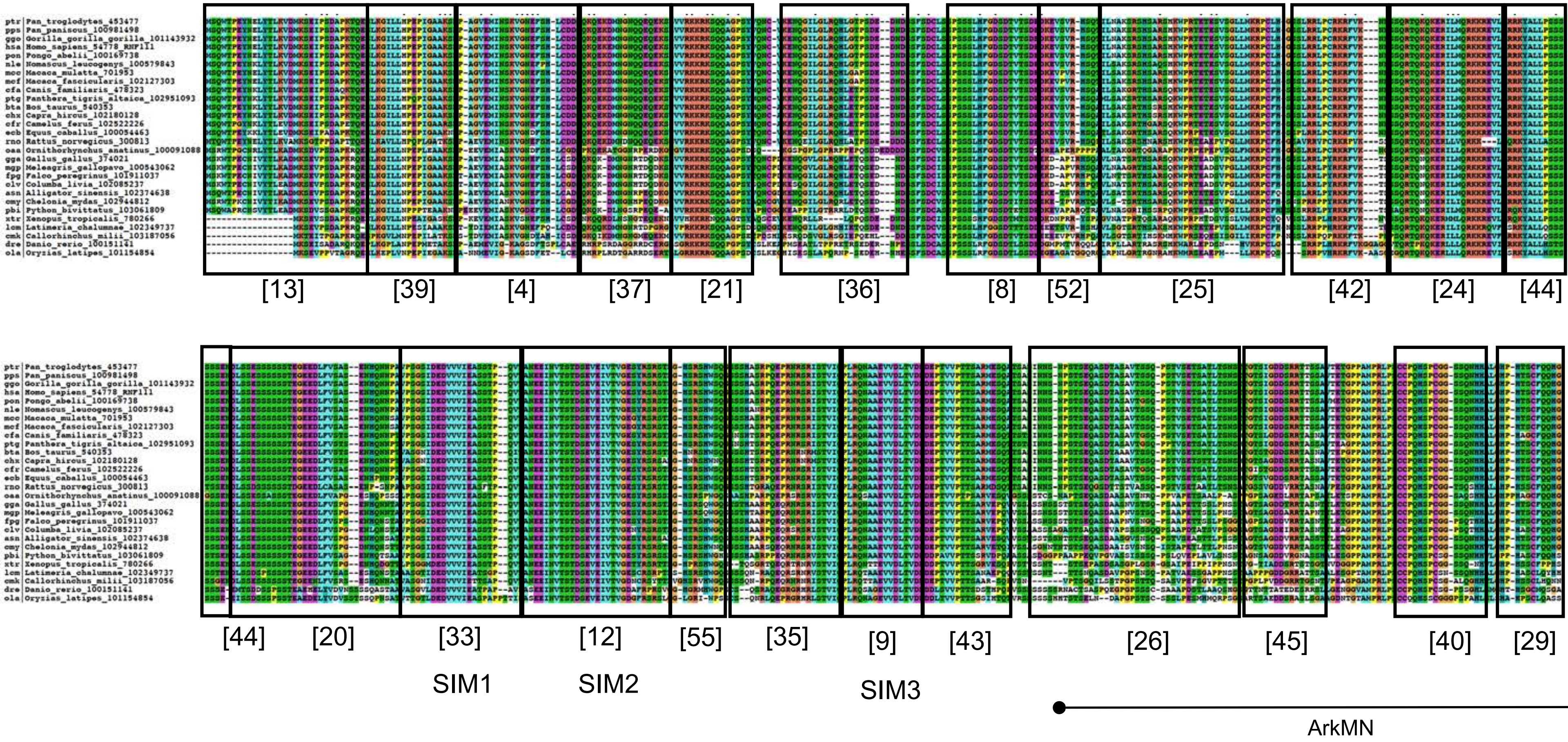
[13]
[39]
[4]
[37]
[21]
[36]
[8]
[52]
[25]
[42]
[24]
[44]
[44]
[20]
[33]
[12]
[55]
[35]
[9]
[43]
[26]
[45]
[40]
[29]
SIM1
SIM2
SIM3
ArkMN

RNF111 (cont.)

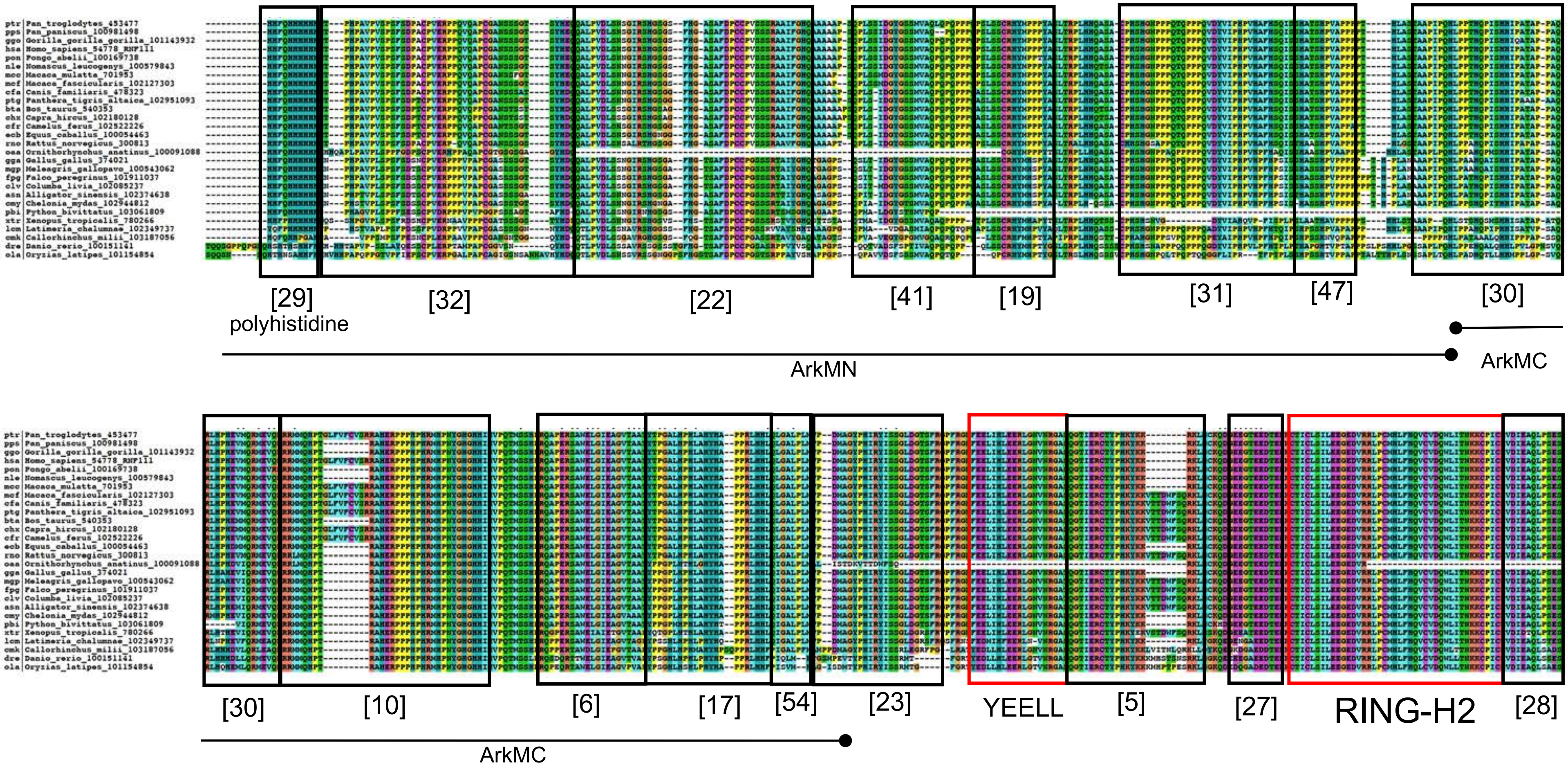
[29]
polyhistidine
[32]
[22]
[41]
[19]
[31]
[47]
[30]
ArkMN
ArkMC
[30]
[10]
[6]
[17]
[54]
[23]
YEELL
[5]
[27]
RING-H2
[28]
ArkMC
