# Supplementary material for: CTLs, a new class of RING-H2 ubiquitin ligases uncovered by YEELL, a motif close to the RING domain that is present across eukaryotes
Source: PLoS One. 2018 Jan 11;13(1):e0190969. doi: 10.1371/journal.pone.0190969 (PMC5764321; doi:10.1371/journal.pone.0190969)

S5 Fig. Protein alignment of plant CTLs.

Group A

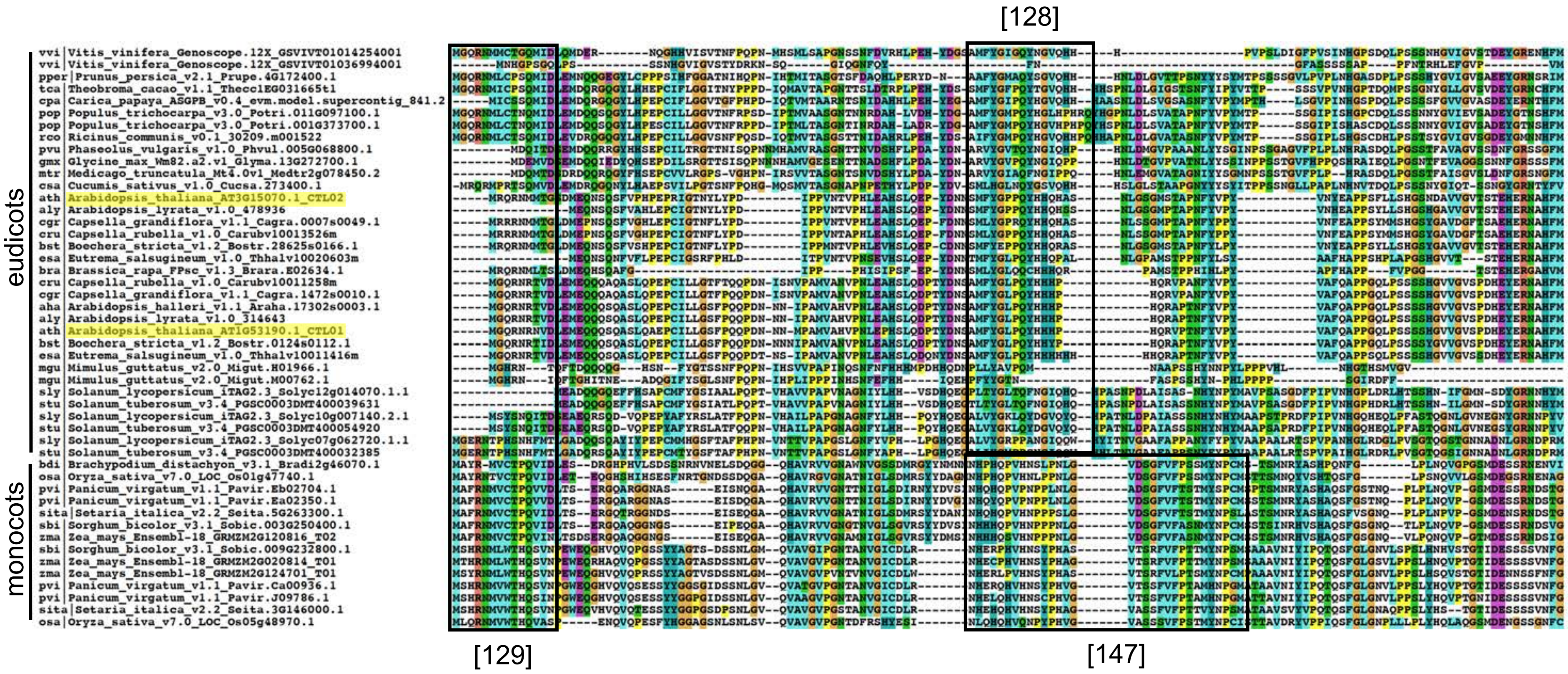

Group A (cont.)

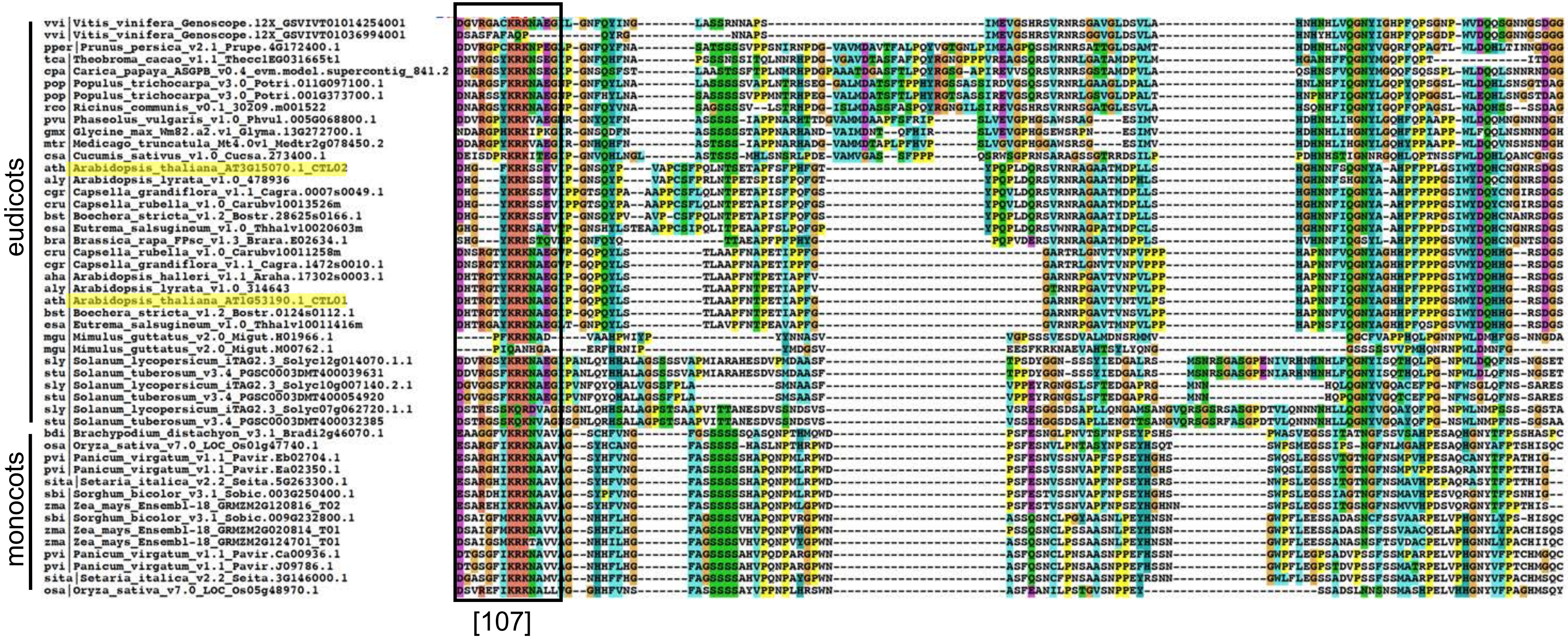

Group A (cont.)

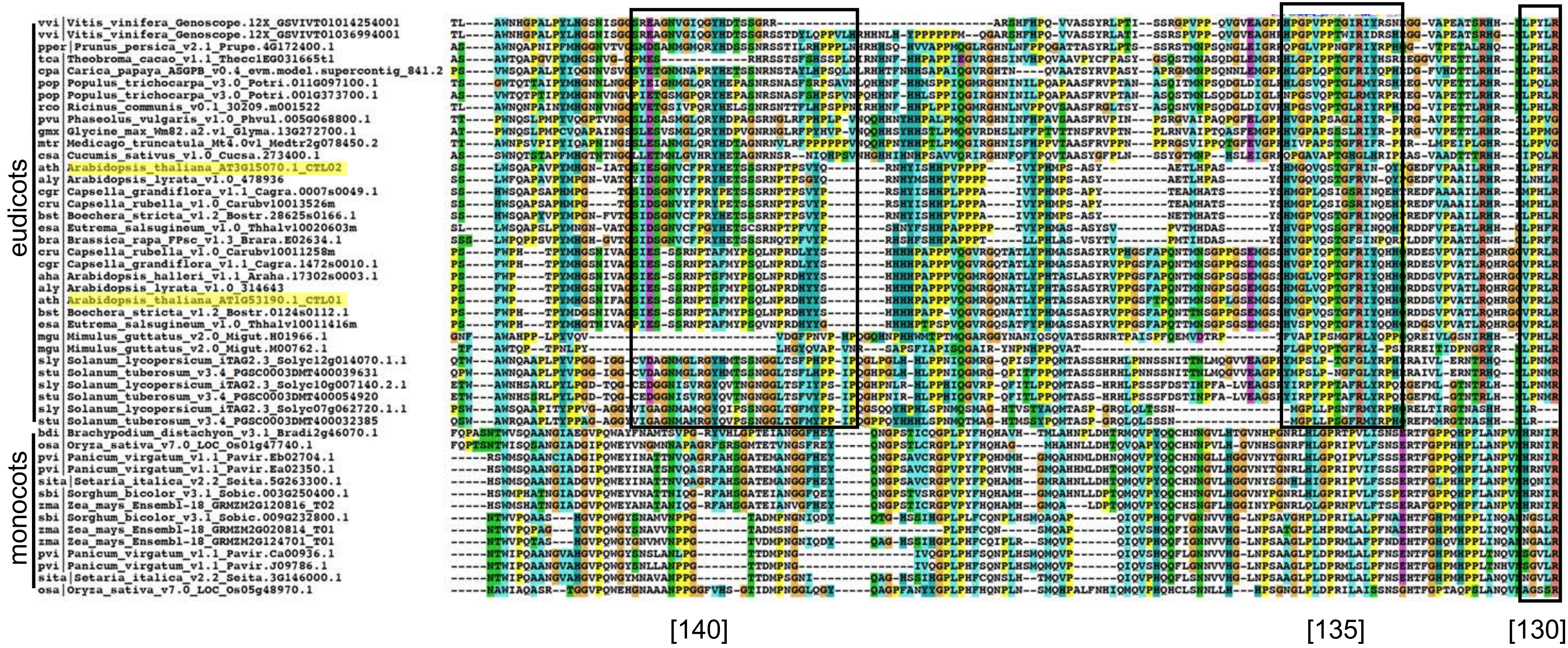

Group A (cont.)

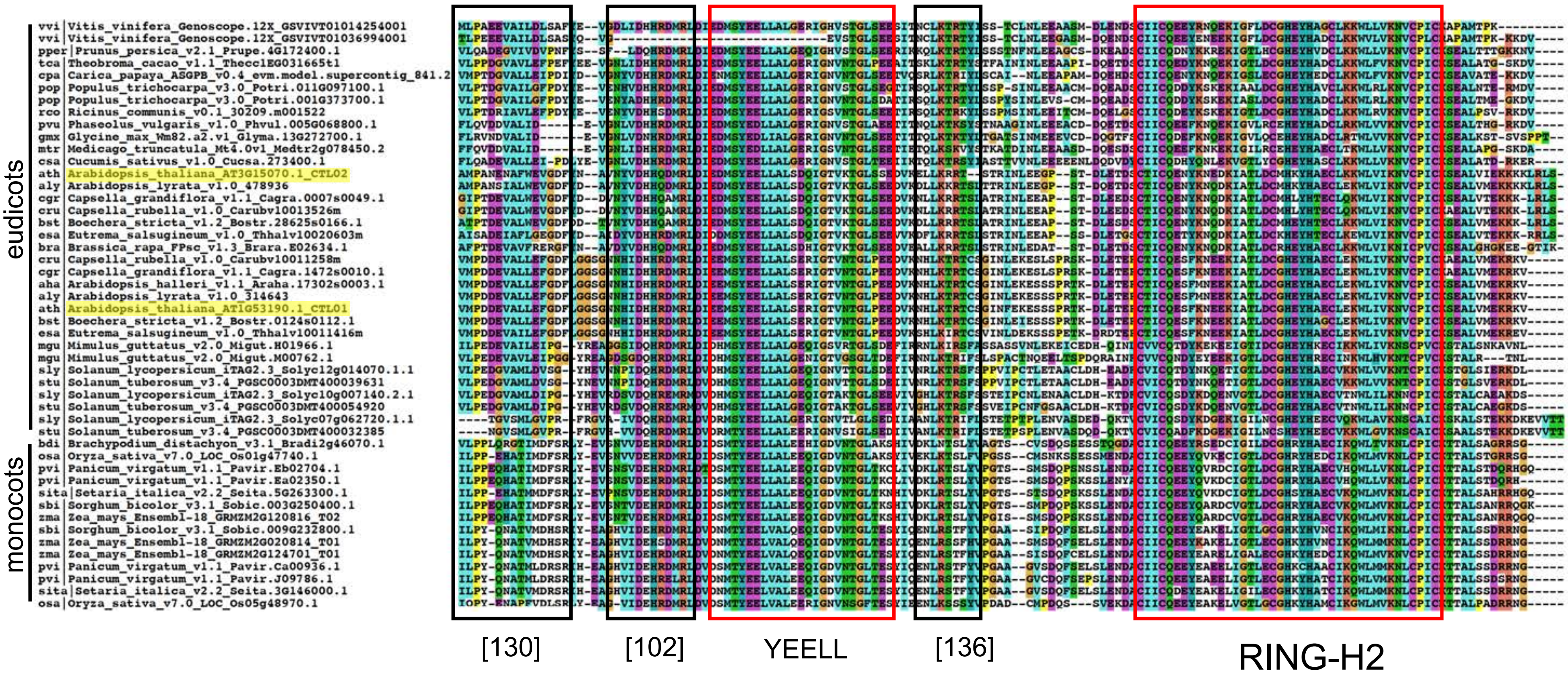

Group B

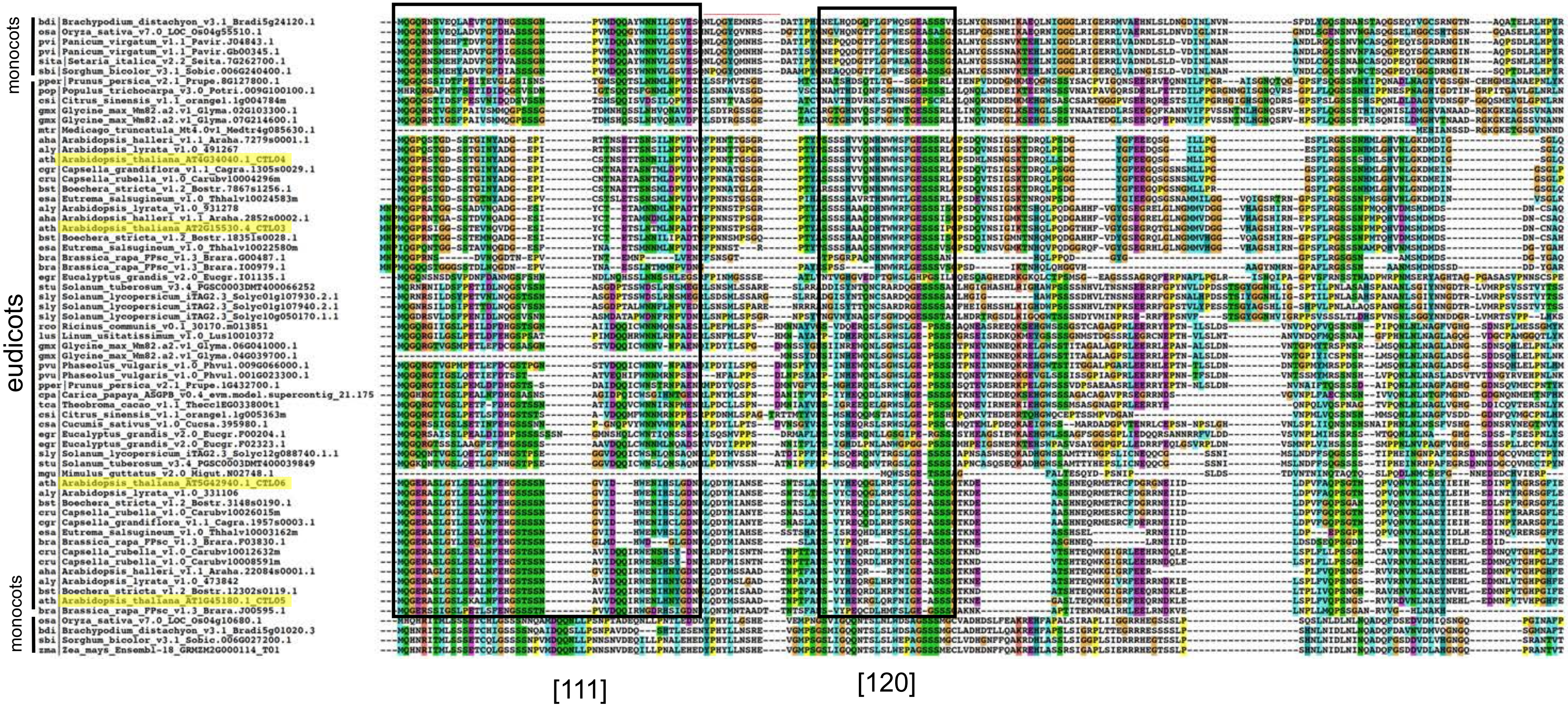

Group B (cont.)

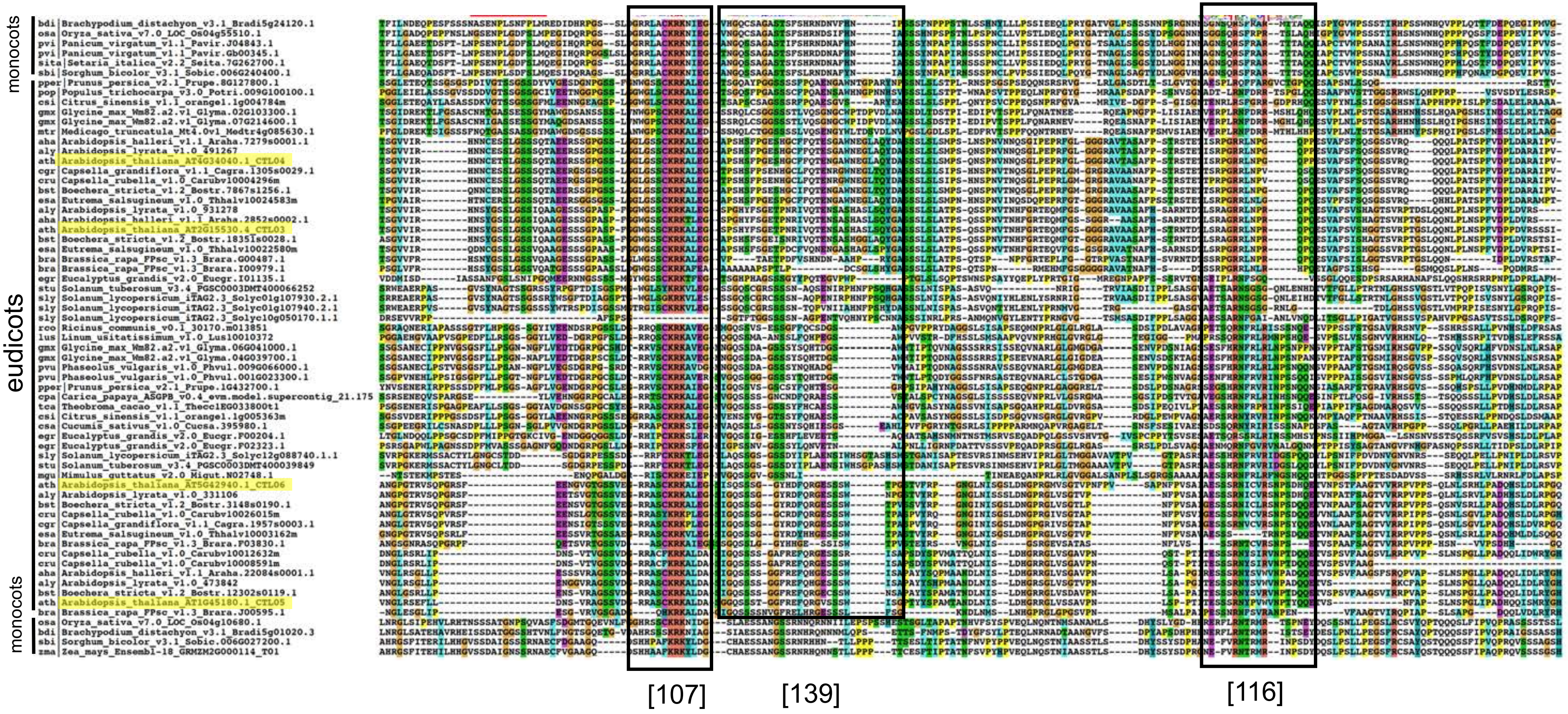

Group B (cont.)

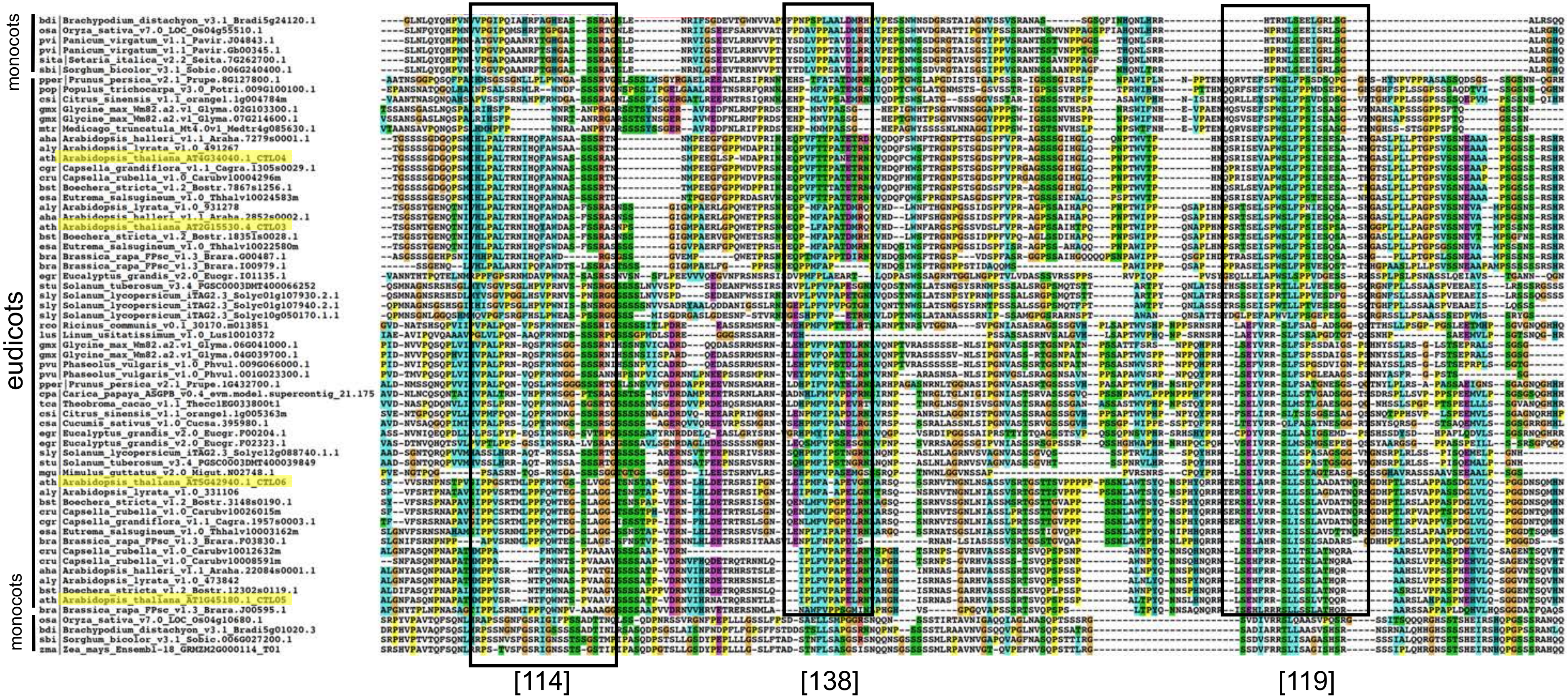

Group B (cont.)

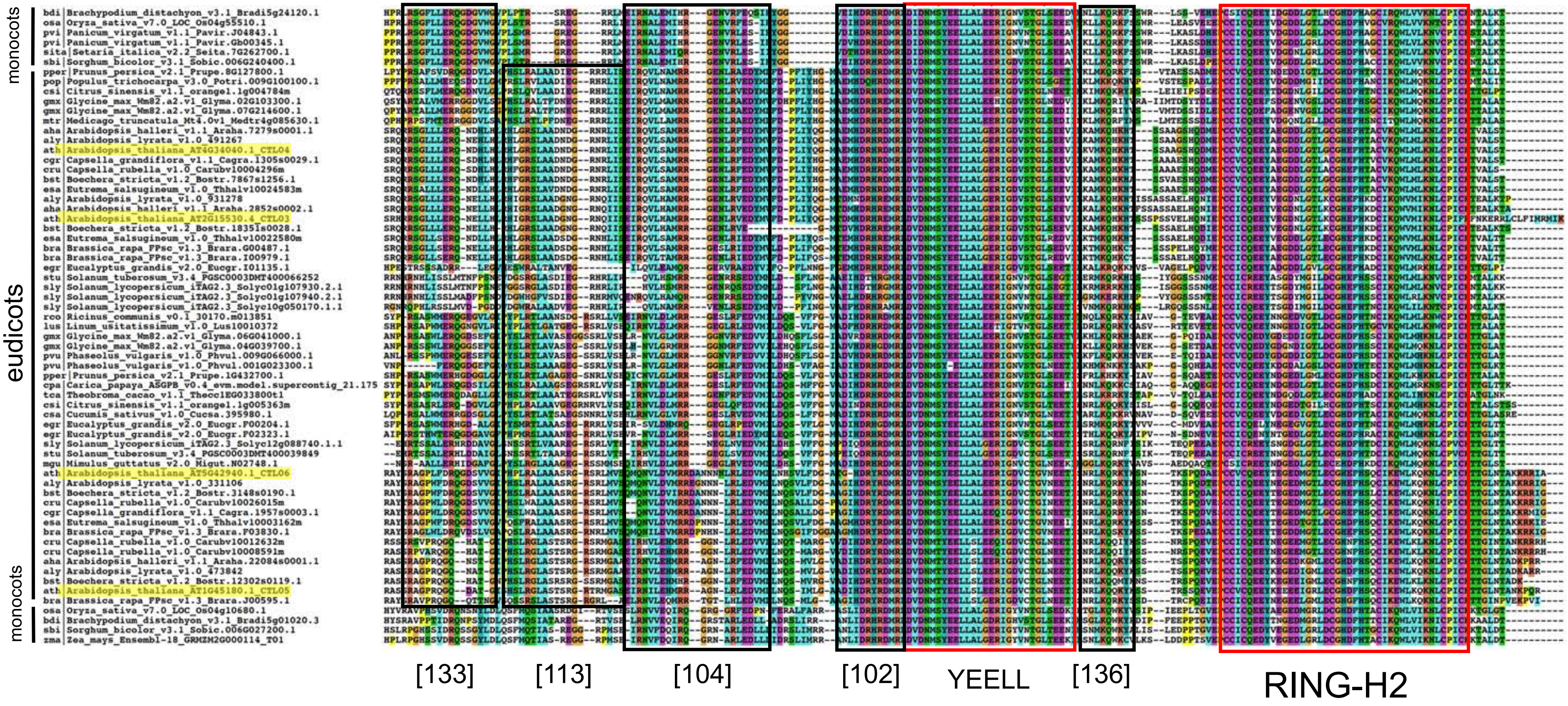
monocots
eudicots
monocots
[133]
[113]
[104]
[102]
YEELL
[136]
RING-H2

Group C

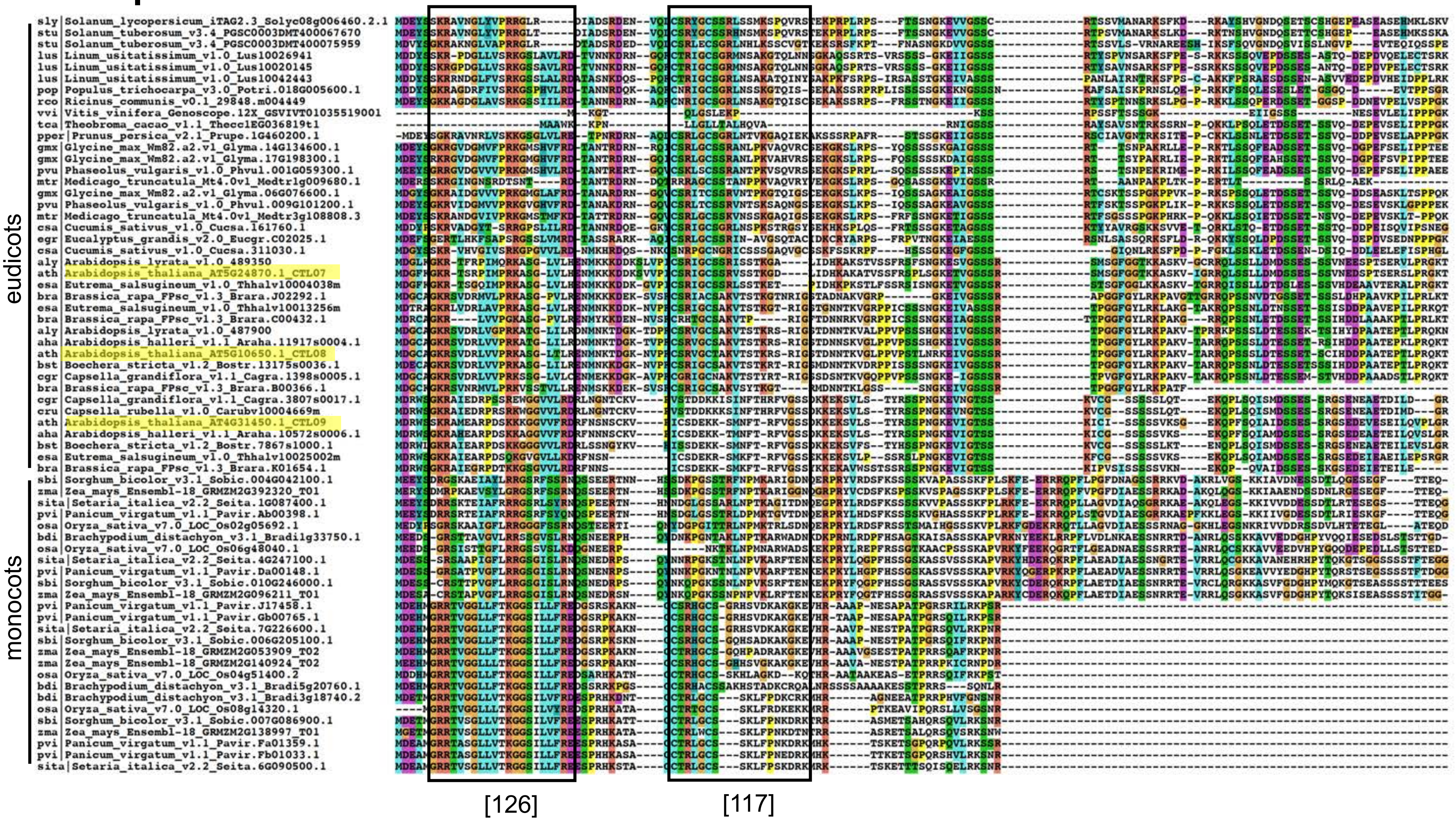

Group C (cont.)

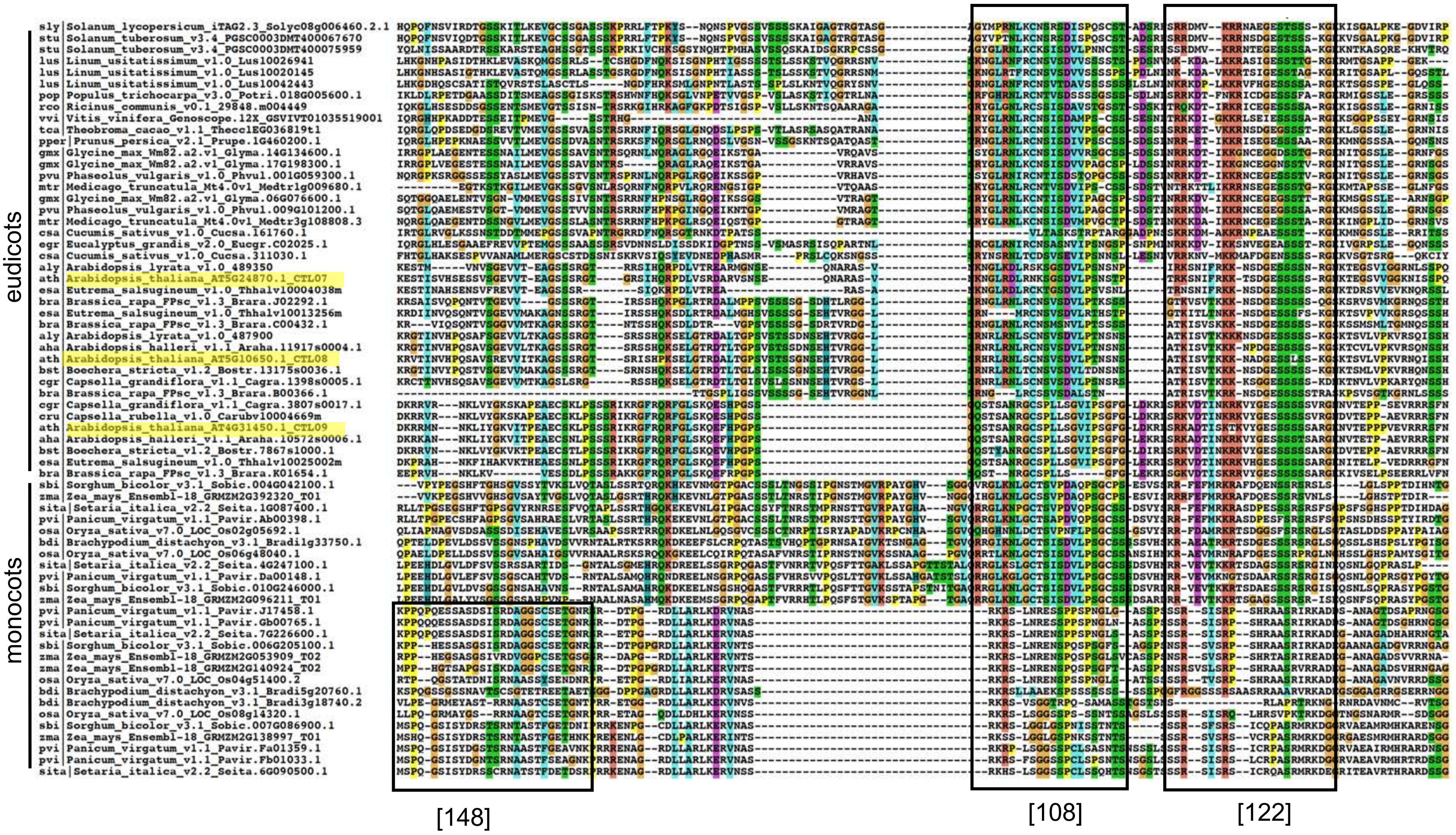

Group C (cont.)

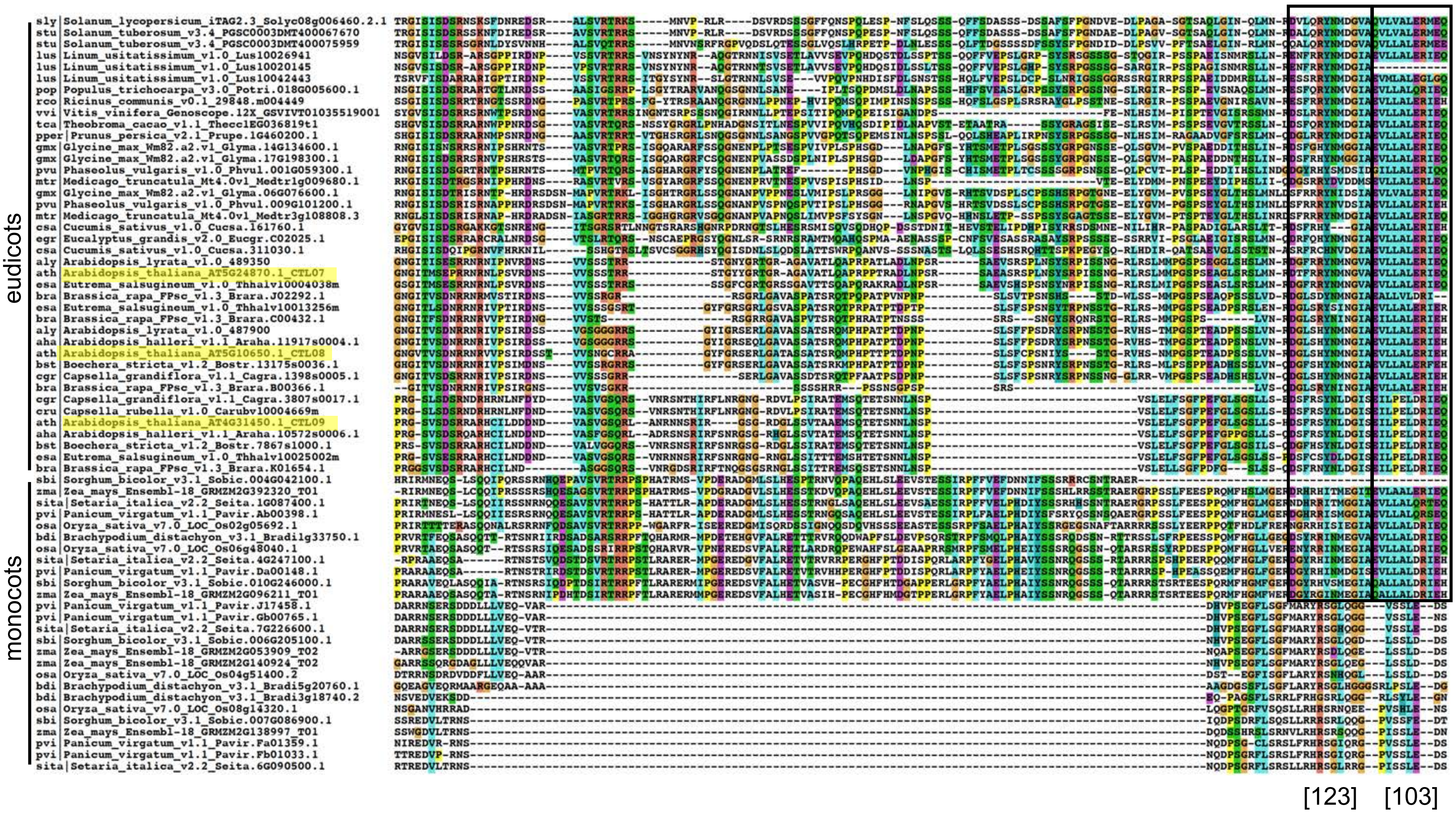
eudicots
monocots
ath|Arabidopsis_thaliana_AT5G24870.1_CTL07
ath|Arabidopsis_thaliana_AT5G10650.1_CTL08
ath|Arabidopsis_thaliana_AT4G31450.1_CTL09
[123]
[103]

Group C (cont.)

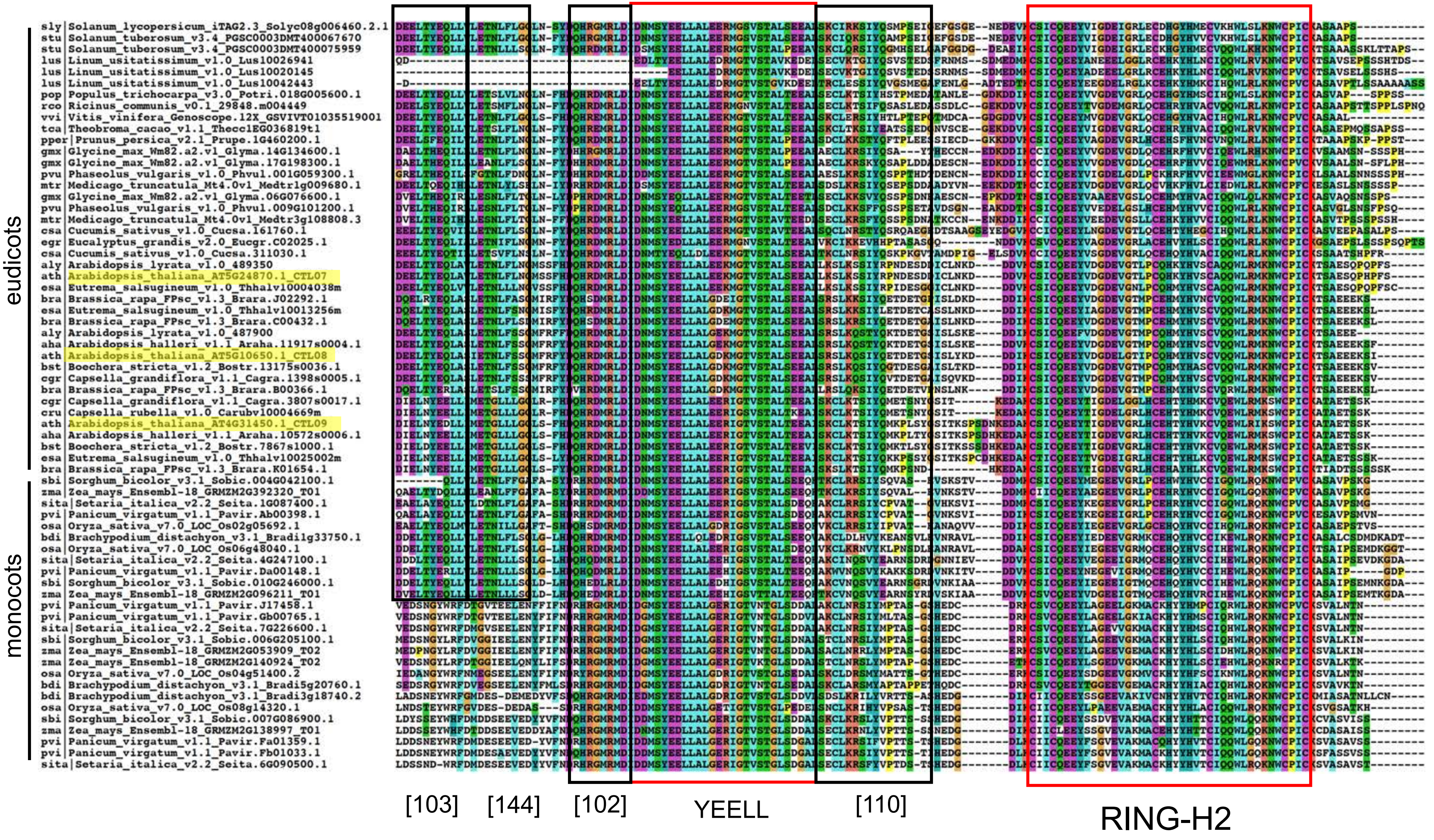

Group D

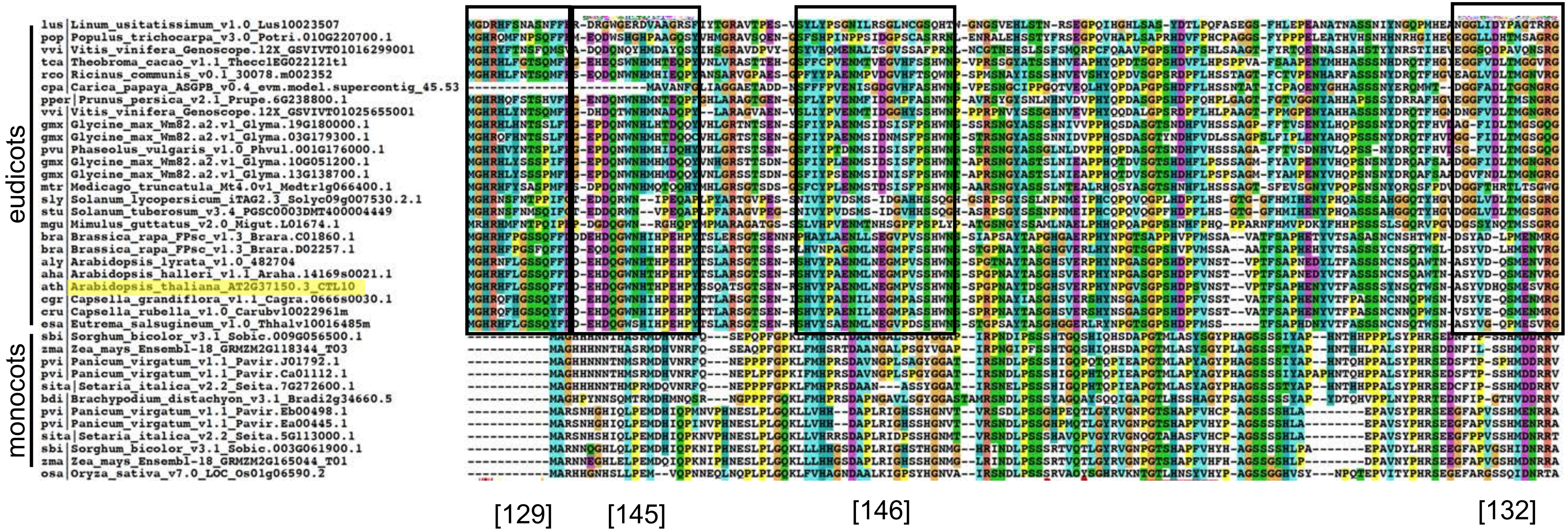

Group D (cont.)

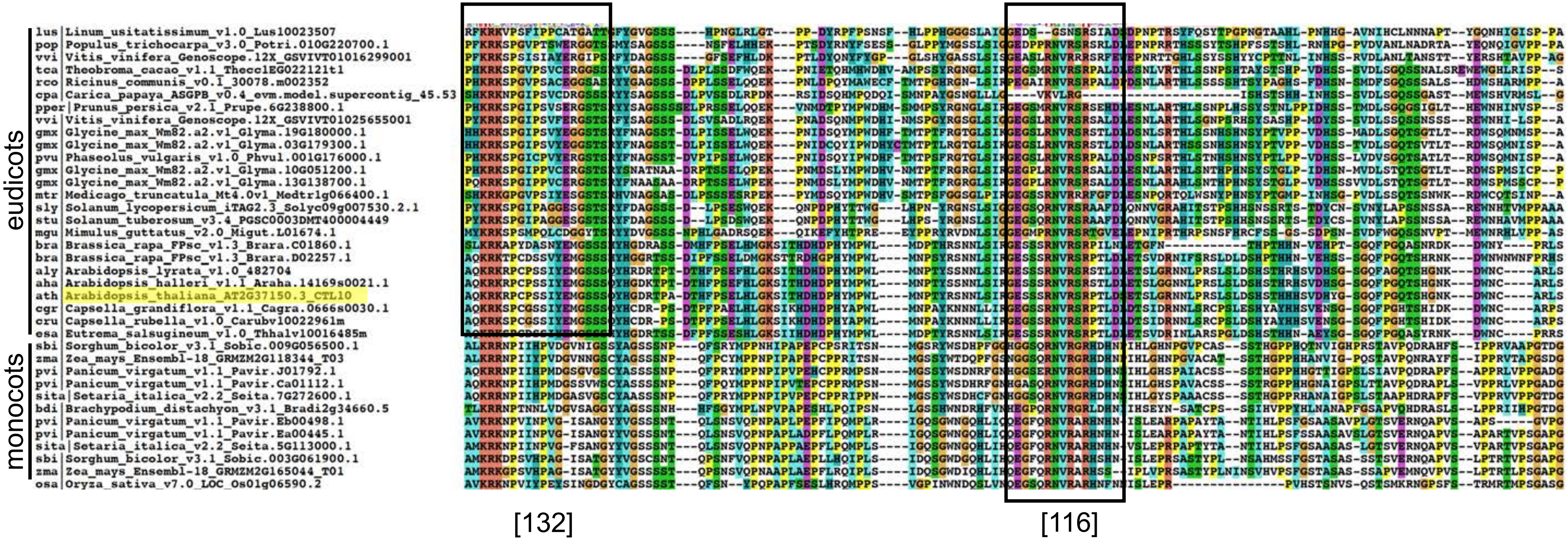

Group D (cont.)

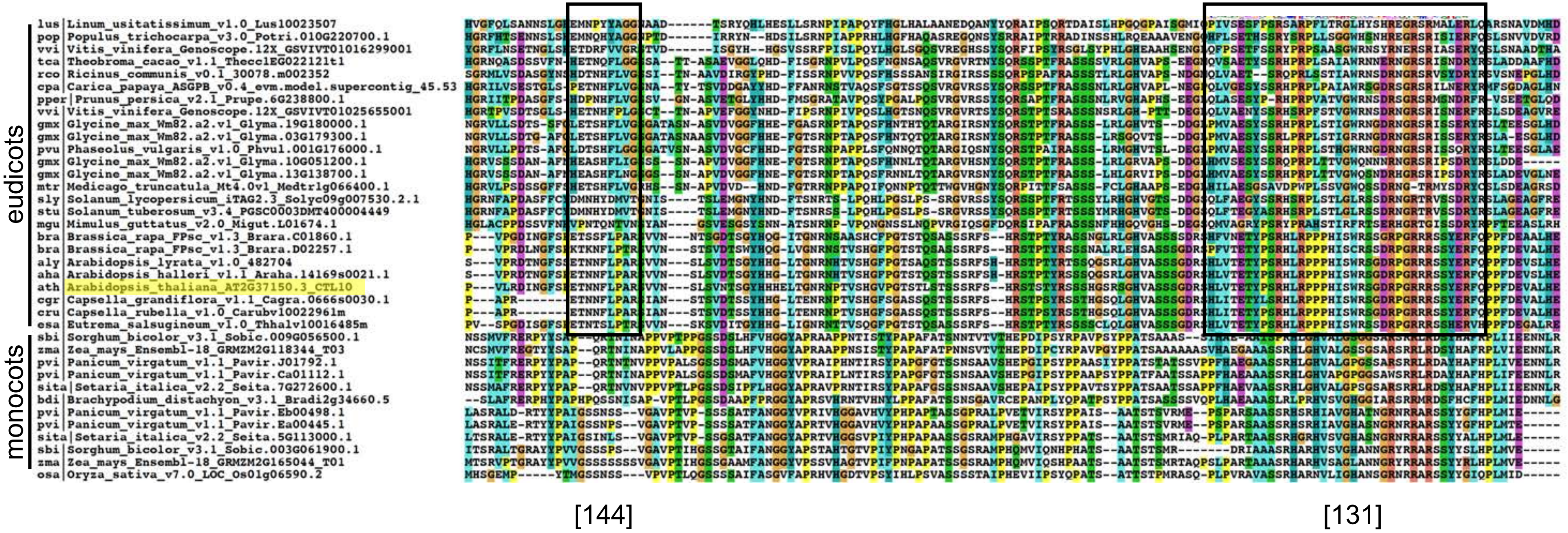

Group D (cont.)

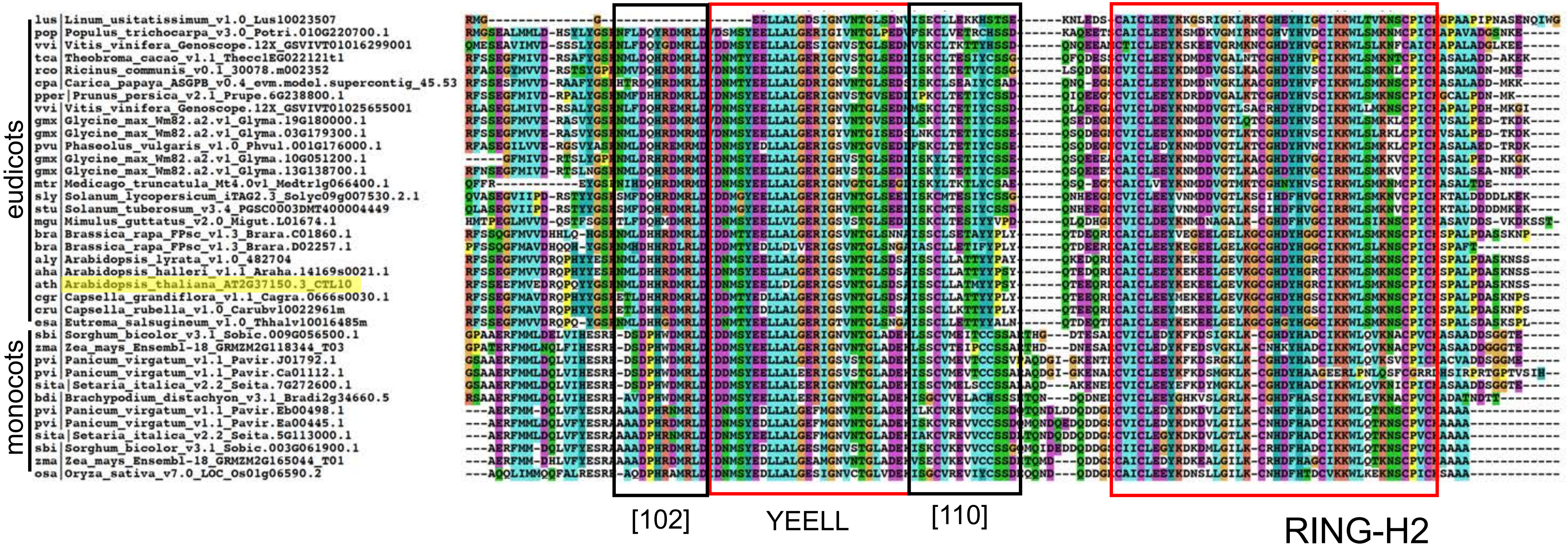

Group E

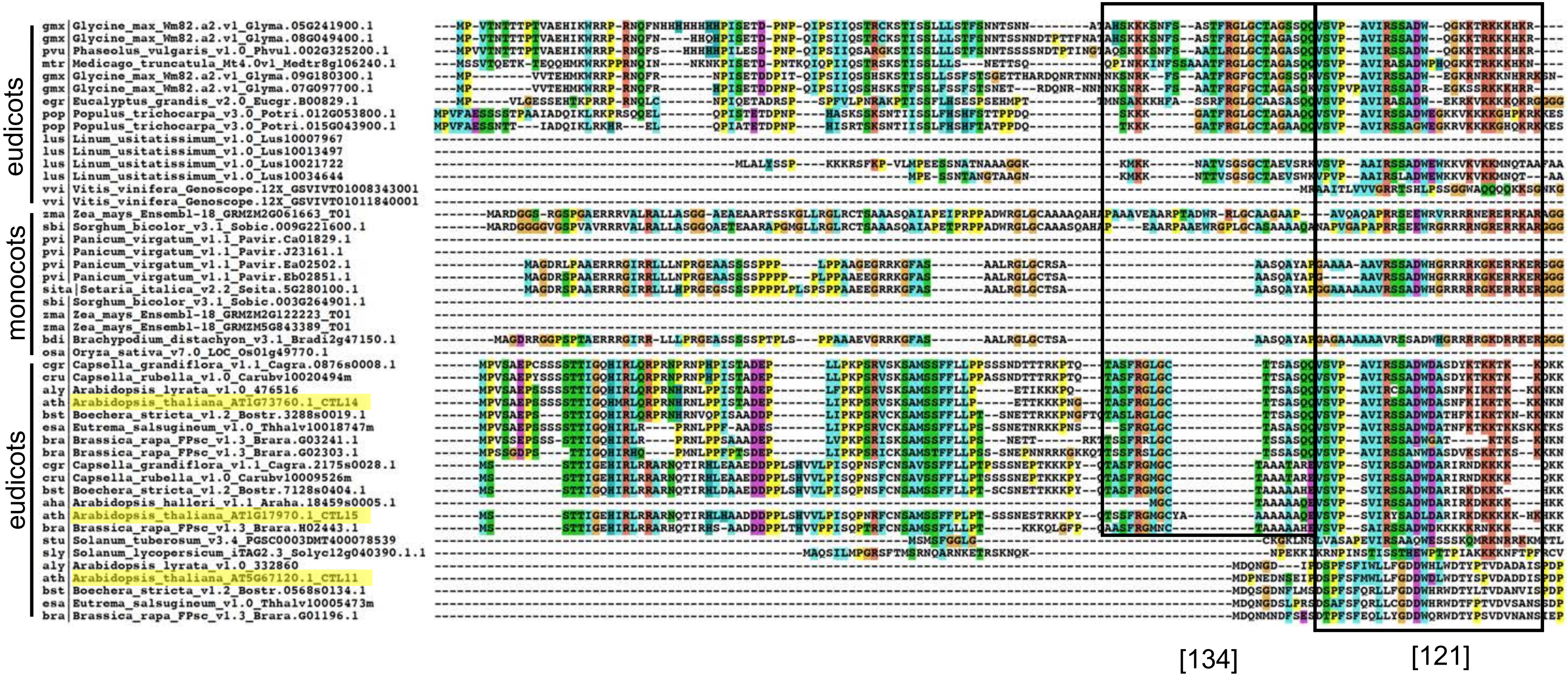

Group E (cont.)

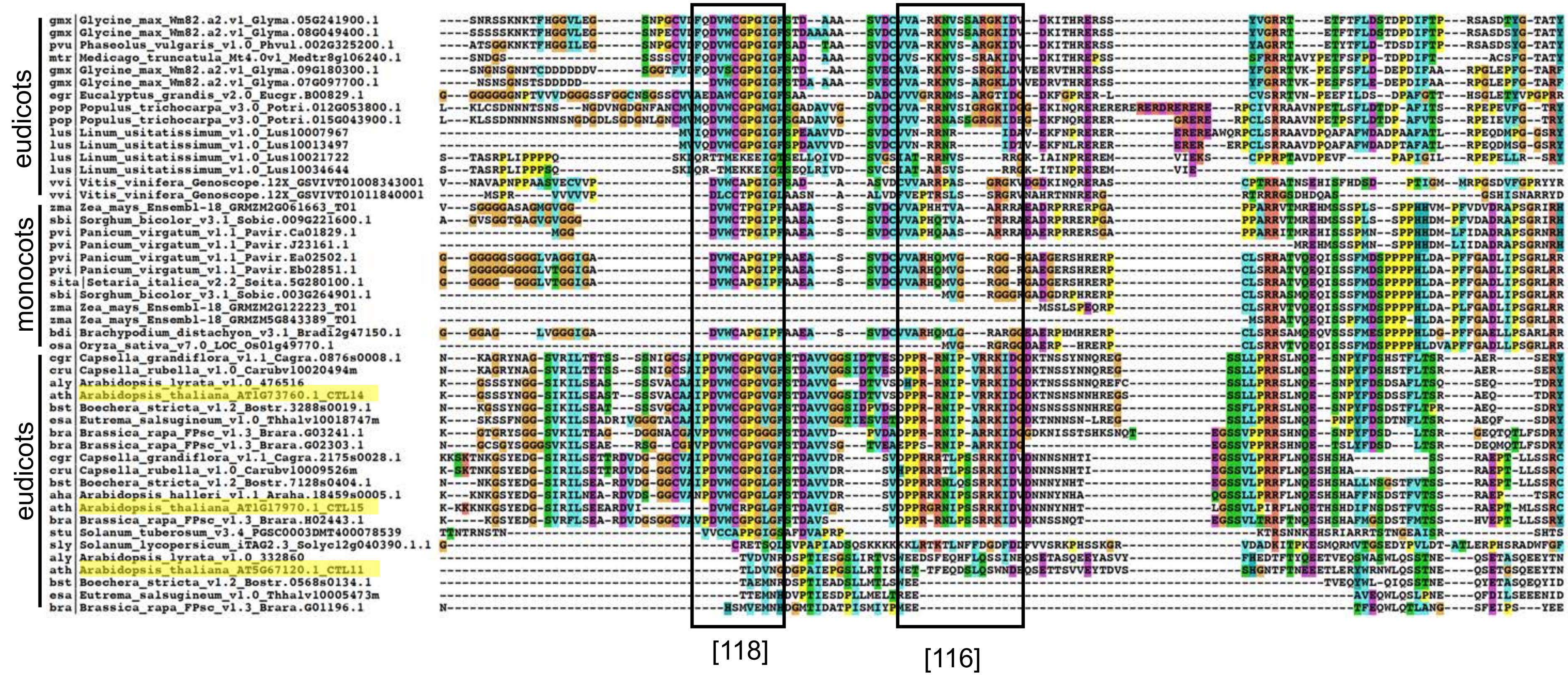

Group E (cont.)

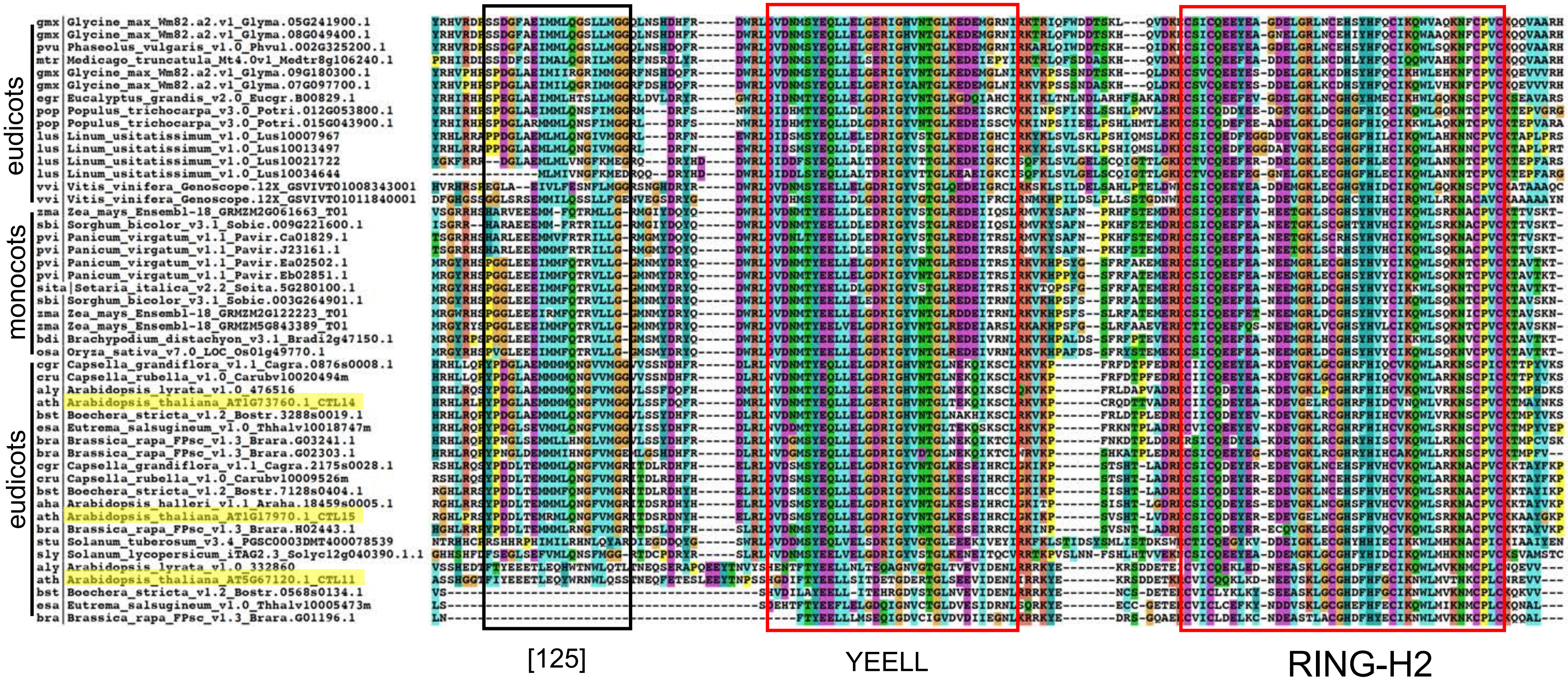

Group F

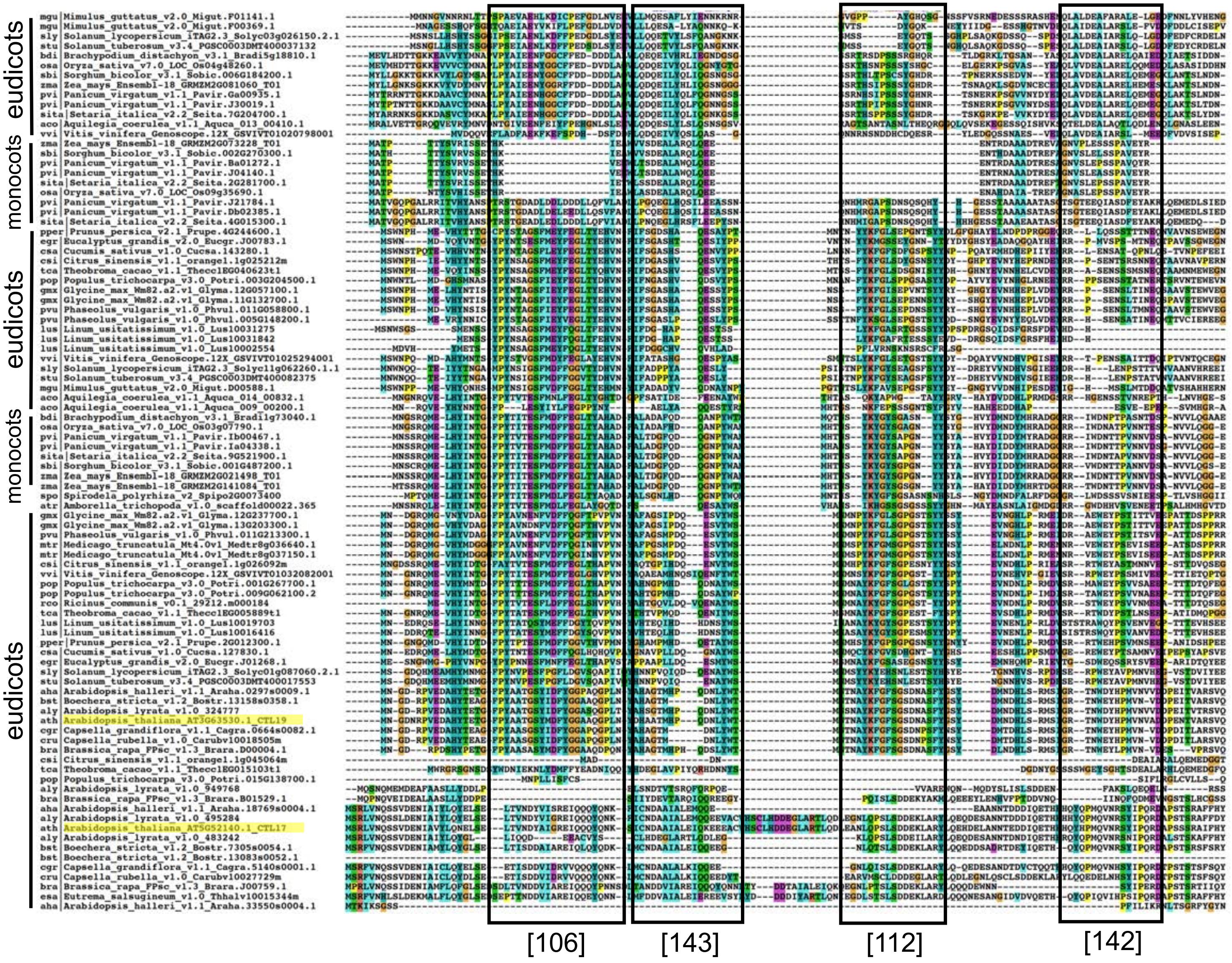
eudicots
monocots
eudicots
monocots
eudicots
[106]
[143]
[112]
[142]

Group F (cont.)

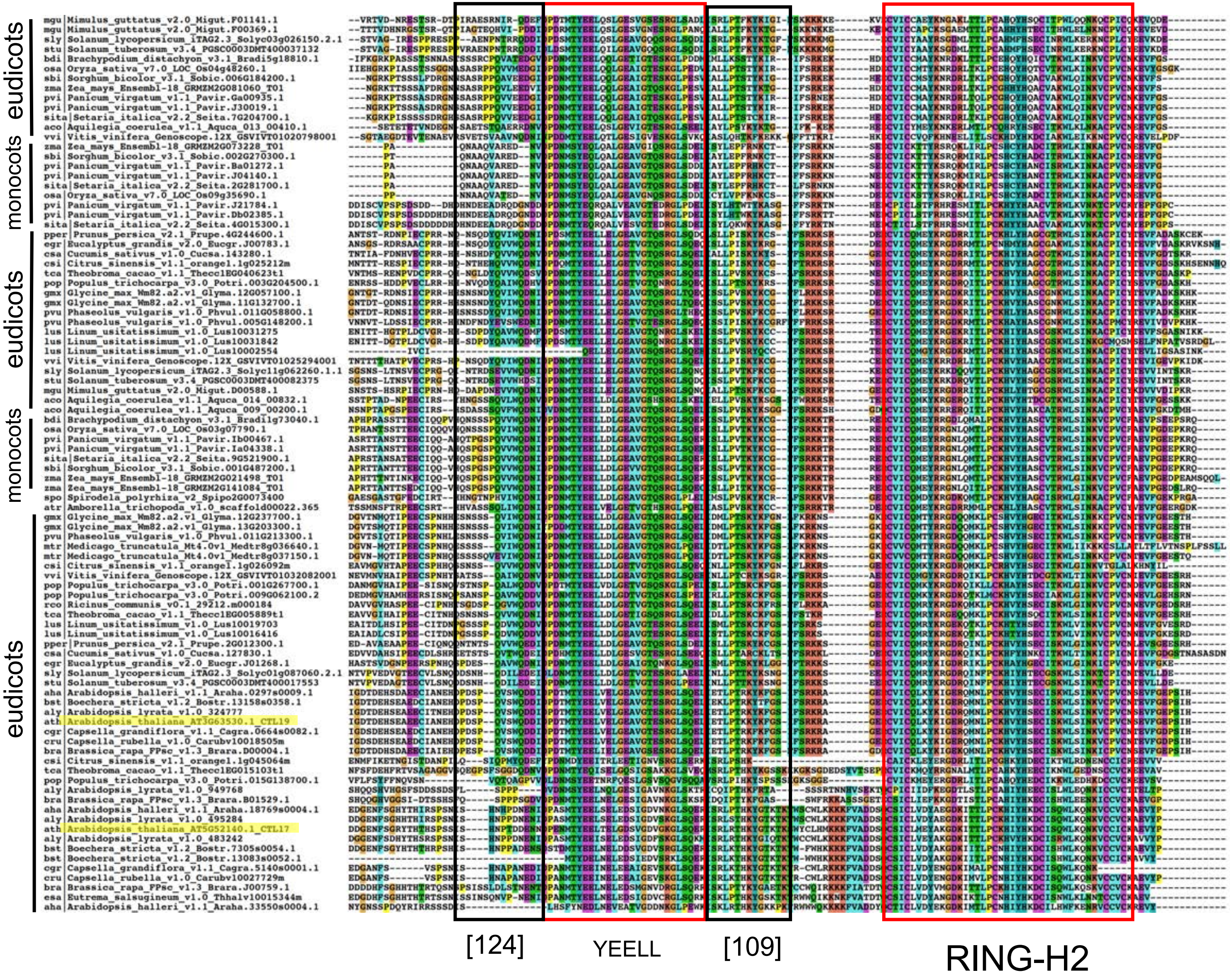
eudicots
monocots
eudicots
monocots
eudicots
[124]
YEELL
[109]
RING-H2

Group G

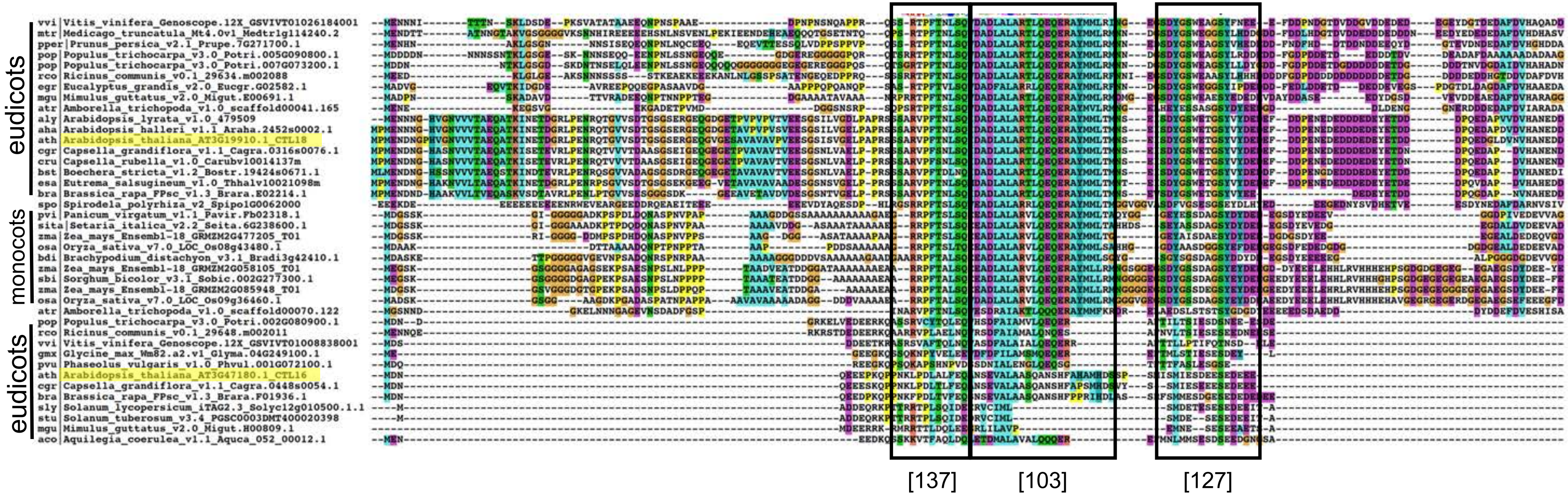

Group G (cont.)

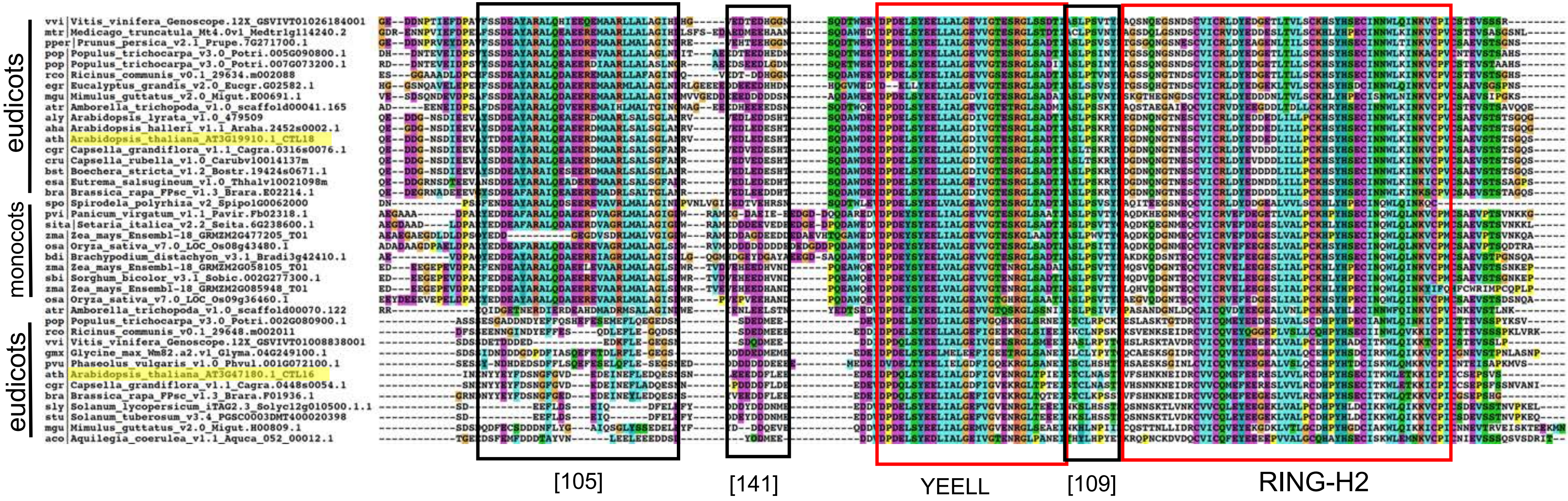

Group H

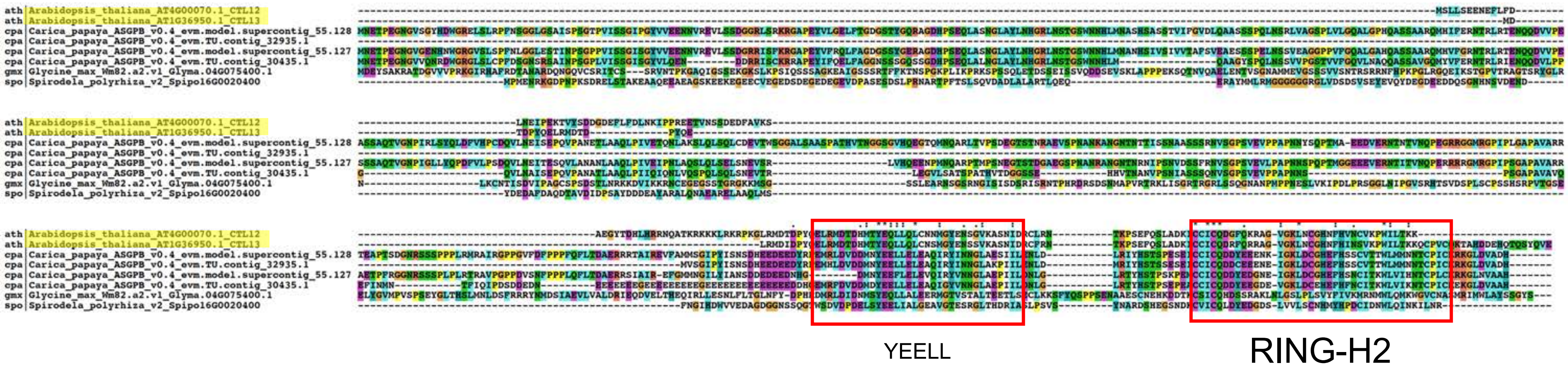

Green algae

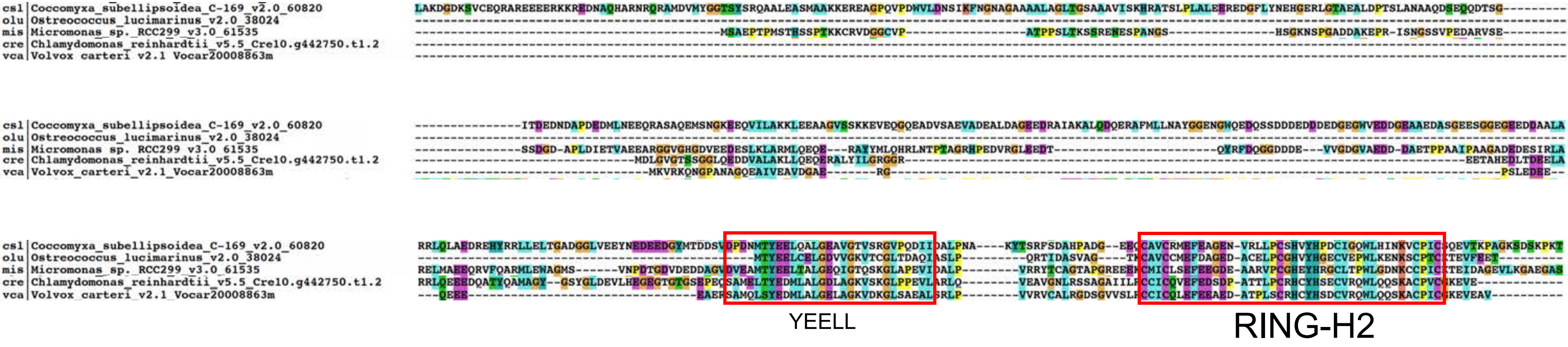

Supplement: S5 Fig — The sequence alignments were performed as described in S4 Fig. (PDF) [file pone.0190969.s005.pdf]
