# Supplementary material for: CTLs, a new class of RING-H2 ubiquitin ligases uncovered by YEELL, a motif close to the RING domain that is present across eukaryotes
Source: PLoS One. 2018 Jan 11;13(1):e0190969. doi: 10.1371/journal.pone.0190969 (PMC5764321; doi:10.1371/journal.pone.0190969)
Supplement: S1 Table — (PDF) [file pone.0190969.s006.pdf]

S1 Table. Lists and number of retrieved genes from animals, fungi, protists and plants.

No.	Category	Abbreviation|Species	Genes
1	Basal embryophytes	**ppp|*Physcomitrella patens v3.3***	Pp3c19_21510V3.1
			Pp3c22_1990V3.1
			Pp3c5_17300V3.1
2		**smo|*Selaginella moellendorffii* v1.0**	SELMODRAFT_104716
			SELMODRAFT_89607
			SELMODRAFT_448412
			SELMODRAFT_447294
			SELMODRAFT_19194
3	Basal angiosperm	**atr|*Amborella trichopoda* v1.0**	scaffold00046.15
			scaffold00021.151
			scaffold00144.13
			scaffold00034.19
			scaffold00019.349
			scaffold00069.77
			scaffold00041.165
			scaffold00022.365
			scaffold00070.122
4	Monocots	**spo|*Spirodela polyrhiza* v2**	Spipo10G0050600
			Spipo31G0002500
			Spipo23G0034800
			Spipo10G0044400
			Spipo10G0049700
			Spipo26G0022400
			Spipo12G0057200
			Spipo2G0073400
			Spipo1G0062000
			Spipo16G0020400
			Spipo0G0151900
5		**bdi|*Brachypodium distachyon* v3.1**	Bradi5g24120.1
			Bradi2g47150.1
			Bradi5g01020.3
			Bradi3g04020.1
			Bradi1g33750.1
			Bradi2g34660.5
			Bradi2g46070.1
			Bradi2g16490.1
			Bradi5g20760.1
			Bradi3g18740.2
			Bradi1g73040.1
			Bradi3g42410.1
			Bradi4g36320.1
			Bradi5g18810.1
			Bradi1g49490.3
			Bradi2g34010.2
			Bradi2g26990.1
6		**osa|*Oryza sativa* v7.0**	LOC_Os01g49770.1
			LOC_Os02g05692.1
			LOC_Os01g47740.1
			LOC_Os05g07070.1
			LOC_Os01g06590.2
			LOC_Os05g48970.1
			LOC_Os04g51400.2

		LOC_Os08g14320.1
		LOC_Os03g07790.1
		LOC_Os09g35690.1
		LOC_Os04g48260.1
		LOC_Os04g55510.1
		LOC_Os06g48040.1
		LOC_Os04g10680.1
		LOC_Os05g47670.1
		LOC_Os08g43480.1
		LOC_Os09g36460.1
		LOC_Os06g03580.2
7	**pvi|*Panicum virgatum* v1.1**	Pavir.Gb00345.1
		Pavir.J04843.1
		Pavir.J03996.1
		Pavir.J06787.1
		Pavir.Ea02502.1
		Pavir.Eb02851.1
		Pavir.Ab00398.1
		Pavir.Ca01829.1
		Pavir.Eb02704.1
		Pavir.Ea02350.1
		Pavir.J23161.1
		Pavir.Ca01112.1
		Pavir.Ca00936.1
		Pavir.Da00148.1
		Pavir.Db00274.1
		Pavir.J01792.1
		Pavir.J09786.1
		Pavir.Ea00445.1
		Pavir.Fa01359.1
		Pavir.Fb01033.1
		Pavir.J17458.1
		Pavir.Gb00765.1
		Pavir.Eb00498.1
		Pavir.Aa03555.1
		Pavir.Ib00467.1
		Pavir.Ia04338.1
		Pavir.J20390.1
		Pavir.Ba01272.1
		Pavir.J04140.1
		Pavir.J13451.1
		Pavir.Ga00935.1
		Pavir.J30019.1
		Pavir.Db02385.1
		Pavir.Fb02318.1
		Pavir.J21784.1
8	**sita|*Setaria italica* v2.2**	Seita.7G262700.1
		Seita.7G032800.1
		Seita.1G087400.1
		Seita.3G158100.1
		Seita.5G263300.1
		Seita.5G280100.1
		Seita.3G146000.1
		Seita.7G272600.1
		Seita.5G113000.1
		Seita.4G247100.1

			Seita.6G090500.1<br>Seita.7G226600.1<br>Seita.9G521900.1<br>Seita.2G281700.1<br>Seita.4G015300.1<br>Seita.7G204700.1<br>Seita.6G238600.1<br>Seita.2G288200.1
9		**sbi|*Sorghum bicolor* v3.1**	Sobic.006G240400.1<br>Sobic.006G027200.1<br>Sobic.003G264901.1<br>Sobic.009G232800.1<br>Sobic.004G042100.1<br>Sobic.003G250400.1<br>Sobic.009G221600.1<br>Sobic.009G056500.1<br>Sobic.003G061900.1<br>Sobic.010G246000.1<br>Sobic.007G086900.1<br>Sobic.006G205100.1<br>Sobic.001G487200.1<br>Sobic.002G270300.1<br>Sobic.007G178100.1<br>Sobic.006G184200.1<br>Sobic.002G277300.1<br>Sobic.007G177801.1<br>Sobic.010G015800.1
10		**zma|*Zea mays* Ensembl-18**	GRMZM2G174926_T01<br>GRMZM2G000114_T01<br>GRMZM2G392320_T01<br>GRMZM5G843389_T01<br>GRMZM2G124701_T01<br>GRMZM2G120816_T02<br>GRMZM2G061663_T01<br>GRMZM2G020814_T01<br>GRMZM2G122223_T01<br>GRMZM2G165044_T01<br>GRMZM2G118344_T03<br>GRMZM2G305901_T01<br>GRMZM2G138997_T01<br>GRMZM2G053909_T02<br>GRMZM2G140924_T02<br>GRMZM2G120136_T01<br>GRMZM2G141084_T01<br>GRMZM2G021498_T01<br>GRMZM2G073228_T01<br>GRMZM2G096211_T01<br>GRMZM2G081060_T01<br>GRMZM2G058105_T01<br>GRMZM2G085948_T01<br>GRMZM2G477205_T01<br>GRMZM2G050973_T01<br>GRMZM2G108084_T01
11	Eudicots	**aco|*Aquilegia coerulea* v1.1**	Aquca_015_00023.1<br>Aquca_007_00704.1<br>Aquca_027_00056.1

		Aquca_016_00083.1<br>Aquca_051_00081.1<br>Aquca_031_00097.1<br>Aquca_024_00101.1<br>Aquca_041_00093.1<br>Aquca_009_00200.1<br>Aquca_014_00832.1<br>Aquca_013_00410.1<br>Aquca_014_00904.1<br>Aquca_052_00012.1
12	**mgu|*Mimulus guttatus* v2.0**	Migut.C00560.1<br>Migut.N02748.1<br>Migut.J00432.1<br>Migut.A00438.1<br>Migut.L01674.1<br>Migut.D01604.1<br>Migut.H01966.1<br>Migut.M00762.1<br>Migut.E00374.1<br>Migut.B01311.1<br>Migut.D00588.1<br>Migut.F01141.1<br>Migut.E00691.1<br>Migut.F00369.1<br>Migut.H00809.1
13	**sly|*Solanum lycopersicum* iTAG2.3**	Solyc12g088740.1.1<br>Solyc04g078680.2.1<br>Solyc10g050170.1.1<br>Solyc01g107940.2.1<br>Solyc01g107930.2.1<br>Solyc12g014070.1.1<br>Solyc08g006460.2.1<br>Solyc12g096420.1.1<br>Solyc07g062720.1.1<br>Solyc09g007530.2.1<br>Solyc03g118840.2.1<br>Solyc10g007140.2.1<br>Solyc12g040390.1.1<br>Solyc05g008840.1.1<br>Solyc01g087060.2.1<br>Solyc03g026150.2.1<br>Solyc12g010500.1.1<br>Solyc11g062260.1.1
14	**stu|*Solanum tuberosum* v3.4**	PGSC0003DMT400020528<br>PGSC0003DMT400039849<br>PGSC0003DMT400066252<br>PGSC0003DMT400039631<br>PGSC0003DMT400079217<br>PGSC0003DMT400067670<br>PGSC0003DMT400032385<br>PGSC0003DMT400004449<br>PGSC0003DMT400014732<br>PGSC0003DMT400054920<br>PGSC0003DMT400075959<br>PGSC0003DMT400078539<br>PGSC0003DMT400017553

		PGSC0003DMT400082375
		PGSC0003DMT400037132
		PGSC0003DMT400020398
15	**vvi|*Vitis vinifera* Genoscope.12X**	GSVIVT01009466001
		GSVIVT01031717001
		GSVIVT01014254001
		GSVIVT01008343001
		GSVIVT01012873001
		GSVIVT01035519001
		GSVIVT01025655001
		GSVIVT01022110001
		GSVIVT01016299001
		GSVIVT01022103001
		GSVIVT01022104001
		GSVIVT01011840001
		GSVIVT01022107001
		GSVIVT01022106001
		GSVIVT01032082001
		GSVIVT01025294001
		GSVIVT01036994001
		GSVIVT01018684001
		GSVIVT01008838001
		GSVIVT01026184001
		GSVIVT01020798001
16	**egr|*Eucalyptus grandis* v2.0**	Eucgr.F02323.1
		Eucgr.F00204.1
		Eucgr.I01135.1
		Eucgr.K03219.1
		Eucgr.F01182.1
		Eucgr.C02025.1
		Eucgr.B00829.1
		Eucgr.J01268.1
		Eucgr.J00783.1
		Eucgr.G02582.1
		Eucgr.J00781.1
17	**pop|*Populus trichocarpa* v3.0**	Potri.002G124300.1
		Potri.001G304900.1
		Potri.009G100100.1
		Potri.018G005600.1
		Potri.010G220700.1
		Potri.011G097100.1
		Potri.006G274700.1
		Potri.001G373700.1
		Potri.005G139900.1
		Potri.007G045500.1
		Potri.012G053800.1
		Potri.015G043900.1
		Potri.012G136400.1
		Potri.003G204500.1
		Potri.015G138700.1
		Potri.005G090800.1
		Potri.001G019600.1
		Potri.007G073200.1
		Potri.002G080900.1
		Potri.001G267700.1
		Potri.009G062100.2

18	**lus|*Linum usitatissimum* v1.0**	Lus10010372<br>Lus10040389<br>Lus10007967<br>Lus10013497<br>Lus10024810<br>Lus10034644<br>Lus10021722<br>Lus10005768<br>Lus10017385<br>Lus10023507<br>Lus10042443<br>Lus10026941<br>Lus10031275<br>Lus10019703<br>Lus10016416<br>Lus10010182<br>Lus10031842<br>Lus10002554<br>Lus10020145
19	**rco|*Ricinus communis* v0.1**	30170.m013851<br>30115.m001237<br>29801.m003161<br>29848.m004449<br>30209.m001522<br>30078.m002352<br>29747.m001086<br>29212.m000184<br>29648.m002011<br>29634.m002088<br>29794.m003495
20	**csi|*Citrus sinensis* v1.1**	orange1.1g005363m<br>orange1.1g004784m<br>orange1.1g002483m<br>orange1.1g009501m<br>orange1.1g016305m<br>orange1.1g008677m<br>orange1.1g016946m<br>orange1.1g047787m<br>orange1.1g026092m<br>orange1.1g045064m<br>orange1.1g019980m<br>orange1.1g025212m
21	**cpa|*Carica papaya* ASGPB v0.4**	evm.model.supercontig_21.175<br>evm.model.supercontig_841.2<br>evm.model.supercontig_23.14<br>evm.model.supercontig_45.53<br>evm.model.supercontig_53.60<br>evm.model.supercontig_3.460<br>evm.model.supercontig_55.127<br>evm.TU.contig_30435.1<br>evm.model.supercontig_55.128<br>evm.TU.contig_32935.1
22	**tca|*Theobroma cacao* v1.1**	Thecc1EG033800t1<br>Thecc1EG007084t1<br>Thecc1EG043109t3<br>Thecc1EG036819t1

		Thecc1EG022121t1<br>Thecc1EG031665t1<br>Thecc1EG000650t1<br>Thecc1EG012485t1<br>Thecc1EG005889t1<br>Thecc1EG007711t1<br>Thecc1EG015103t1<br>Thecc1EG040623t1
23	**aha|*Arabidopsis halleri* v1.1**	Araha.22084s0001.1<br>Araha.7279s0001.1<br>Araha.2852s0002.1<br>Araha.10572s0006.1<br>Araha.8919s0009.1<br>Araha.17302s0003.1<br>Araha.11917s0004.1<br>Araha.14169s0021.1<br>Araha.41851s0002.1<br>Araha.19609s0006.1<br>Araha.18459s0005.1<br>Araha.18769s0004.1<br>Araha.23688s0001.1<br>Araha.35247s0006.1<br>Araha.0297s0009.1<br>Araha.2452s0002.1<br>Araha.33550s0004.1
24	**aly|*Arabidopsis lyrata* v1.0**	331106<br>473842<br>491267<br>931278<br>489350<br>314643<br>487900<br>476516<br>482704<br>478936<br>312759<br>495284<br>332860<br>483242<br>479509<br>324777<br>949768
25	**ath|*Arabidopsis thaliana***	AT1G53190.1_CTL01<br>AT3G15070.1_CTL02<br>AT2G15530.4_CTL03<br>AT4G34040.1_CTL04<br>AT1G45180.1_CTL05<br>AT5G42940.1_CTL06<br>AT5G24870.1_CTL07<br>AT5G10650.1_CTL08<br>AT4G31450.1_CTL09<br>AT2G37150.3_CTL10<br>AT5G67120.1_CTL11<br>AT4G00070.1_CTL12<br>AT1G36950.1_CTL13<br>AT1G73760.1_CTL14

		AT1G17970.1_CTL15<br>AT3G47180.1_CTL16<br>AT5G52140.1_CTL17<br>AT3G19910.1_CTL18<br>AT3G63530.1_CTL19
26	**bst|*Boechera stricta* v1.2**	Bostr.3148s0190.1<br>Bostr.12302s0119.1<br>Bostr.7867s1256.1<br>Bostr.18351s0028.1<br>Bostr.7867s1000.1<br>Bostr.0124s0112.1<br>Bostr.5763s0032.1<br>Bostr.13175s0036.1<br>Bostr.3288s0019.1<br>Bostr.23794s0375.1<br>Bostr.28625s0166.1<br>Bostr.7128s0404.1<br>Bostr.0568s0134.1<br>Bostr.7305s0054.1<br>Bostr.19424s0671.1<br>Bostr.13158s0358.1<br>Bostr.13083s0052.1
27	**bra|*Brassica rapa* FPsc v1.3**	Brara.I01765.1<br>Brara.F03830.1<br>Brara.J00595.1<br>Brara.H00442.1<br>Brara.G00487.1<br>Brara.I00979.1<br>Brara.K01654.1<br>Brara.E01471.1<br>Brara.F00132.1<br>Brara.F02695.1<br>Brara.C01860.1<br>Brara.G03241.1<br>Brara.J02292.1<br>Brara.G02303.1<br>Brara.E02634.1<br>Brara.C00432.1<br>Brara.B00366.1<br>Brara.D02257.1<br>Brara.C03555.1<br>Brara.H02443.1<br>Brara.G01196.1<br>Brara.D00004.1<br>Brara.E02214.1<br>Brara.A02844.1<br>Brara.J00759.1<br>Brara.F01936.1<br>Brara.B01529.1<br>Brara.F03694.1
28	**cgr|*Capsella grandiflora* v1.1**	Cagra.1957s0003.1<br>Cagra.1305s0029.1<br>Cagra.3807s0017.1<br>Cagra.1472s0010.1<br>Cagra.0622s0006.1<br>Cagra.0876s0008.1

		Cagra.1398s0005.1
		Cagra.0007s0049.1
		Cagra.0666s0030.1
		Cagra.2175s0028.1
		Cagra.5140s0001.1
		Cagra.0316s0076.1
		Cagra.0664s0082.1
		Cagra.0448s0054.1
29	**cru|*Capsella rubella* v1.0**	Carubv10026015m
		Carubv10012632m
		Carubv10008591m
		Carubv10004296m
		Carubv10004669m
		Carubv10000716m
		Carubv10011258m
		Carubv10020494m
		Carubv10013526m
		Carubv10022961m
		Carubv10009526m
		Carubv10027729m
		Carubv10014137m
		Carubv10018505m
30	**esa|*Eutrema salsugineum* v1.0**	Thhalv10003162m
		Thhalv10011314m
		Thhalv10024583m
		Thhalv10022580m
		Thhalv10004038m
		Thhalv10025002m
		Thhalv10020603m
		Thhalv10011416m
		Thhalv10013256m
		Thhalv10018747m
		Thhalv10016485m
		Thhalv10007935m
		Thhalv10015344m
		Thhalv10005473m
		Thhalv10021098m
31	**csa|*Cucumis sativus* v1.0**	Cucsa.395980.1
		Cucsa.161760.1
		Cucsa.273400.1
		Cucsa.311030.1
		Cucsa.121730.1
		Cucsa.122810.1
		Cucsa.143280.1
		Cucsa.127830.1
		Cucsa.065490.1
		Cucsa.350640.1
32	**gmx|*Glycine max* Wm82.a2.v1**	Glyma.04G039800.1
		Glyma.04G039700.1
		Glyma.06G041000.1
		Glyma.02G103300.1
		Glyma.07G214600.1
		Glyma.17G240900.1
		Glyma.17G198300.1
		Glyma.06G076600.1
		Glyma.04G075400.1

		Glyma.10G051200.1<br>Glyma.14G134600.1<br>Glyma.13G272700.1<br>Glyma.13G138700.1<br>Glyma.05G241900.1<br>Glyma.06G234300.1<br>Glyma.19G180000.1<br>Glyma.03G179300.1<br>Glyma.08G049400.1<br>Glyma.09G180300.1<br>Glyma.10G284900.1<br>Glyma.11G132700.1<br>Glyma.11G156300.1<br>Glyma.12G057100.1<br>Glyma.12G158500.1<br>Glyma.12G237700.1<br>Glyma.13G203300.1<br>Glyma.04G249100.1<br>Glyma.20G104300.1<br>Glyma.07G097700.1
33	**mtr|*Medicago truncatula* Mt4.0v1**	Medtr1g019200.2<br>Medtr4g085630.1<br>Medtr3g108808.3<br>Medtr1g009680.1<br>Medtr1g066400.1<br>Medtr2g078450.2<br>Medtr1g066430.1<br>Medtr2g078440.1<br>Medtr7g100950.1<br>Medtr2g076240.1<br>Medtr8g106240.1<br>Medtr6g082620.1<br>Medtr2g076290.1<br>Medtr8g036640.1<br>Medtr8g037150.1<br>Medtr4g065670.2<br>Medtr1g114240.2
34	**pvu|*Phaseolus vulgaris* v1.0**	Phvul.001G023300.1<br>Phvul.009G066000.1<br>Phvul.005G068800.1<br>Phvul.009G101200.1<br>Phvul.001G059300.1<br>Phvul.002G325200.1<br>Phvul.007G221700.1<br>Phvul.001G176000.1<br>Phvul.004G153500.1<br>Phvul.011G149000.1<br>Phvul.011G153200.1<br>Phvul.010G083200.1<br>Phvul.006G035700.1<br>Phvul.006G043800.1<br>Phvul.010G052600.1<br>Phvul.011G118000.1<br>Phvul.011G213300.1<br>Phvul.005G148200.1<br>Phvul.007G015800.1

			Phvul.011G058800.1<br>Phvul.010G083100.1<br>Phvul.001G072100.1<br>Phvul.003G242800.1
35		**pper|*Prunus persica* v2.1**	Prupe.1G432700.1<br>Prupe.8G127800.1<br>Prupe.1G460200.1<br>Prupe.6G238800.1<br>Prupe.4G172400.1<br>Prupe.5G225100.1<br>Prupe.7G256200.1<br>Prupe.2G012300.1<br>Prupe.7G271700.1<br>Prupe.1G493000.1<br>Prupe.4G244600.1
36	Green algae	**cre|*Chlamydomonas reinhardtii* v5.5**	Cre10.g442750.t1.2
37		**vca|*Volvox carteri* v2.1**	Vocar20008863m
38		**csl|*Coccomyxa subellipsoidea* C-169 v2.0**	60820
39		**mpp|*Micromonas pusilla* CCMP1545 v3.0**	50363
40		**mis|*Micromonas sp.* RCC299 v3.0**	61535
41		**olu|*Ostreococcus lucimarinus* v2.0**	38024
42	Mammals	**has|*Homo sapiens***	494470_RNF165<br>22838_RNF44<br>152006_RNF38<br>54778_RNF111
43		**ptr|*Pan troglodytes***	462290<br>453477<br>473232<br>455400
44		**pps|*Pan paniscus***	100967299<br>100986258<br>100981498<br>100969615
45		**ggo|*Gorilla gorilla gorilla***	101129543<br>101149394<br>101143932<br>101153395
46		**pon|*Pongo abelii***	100169738<br>100452090<br>100460162<br>100452536
47		**nle|*Nomascus leucogenys***	100579724<br>100589364<br>100579843<br>100588605
48		**mcc|*Macaca mulatta***	702911<br>701953<br>693346<br>702104
49		**mcf|*Macaca fascicularis***	102122095<br>102142528<br>102127303<br>101865055
50		**rno|*Rattus norvegicus***	307251<br>361212

			300813<br>171501
51		**cfa|*Canis familiaris***	490468<br>489101<br>478323<br>474766
52		**ptg|*Panthera tigris altaica***	102951265<br>102951582<br>102951093<br>102969536
53		**bta|*Bos taurus***	537072<br>531968<br>540353<br>532877
54		**chx|*Capra hircus***	102181551<br>102175949<br>102180128<br>102185654
55		**cfr|*Camelus ferus***	102517705<br>102522226<br>102516869<br>102519576
56		**ecb|*Equus caballus***	100068804<br>100058652<br>100054463<br>100055331
57		**oaa|*Ornithorhynchus anatinus***	100074786<br>100087339<br>100091088
58	Birds	**gga|*Gallus gallus***	416382<br>416224<br>427281<br>374021
59		**mgp|*Meleagris gallopavo***	100545301<br>100543062<br>100540666<br>100539930
60		**fpg|*Falco peregrinus***	101920044<br>101910974<br>101911037<br>101921893
61		**clv|*Columba livia***	102087327<br>102086969<br>102085237<br>102086520
62	Reptiles	**asn|*Alligator sinensis***	102383738<br>102387145<br>102374638<br>102387251
63		**cmy|*Chelonia mydas***	102944725<br>102944812<br>102929629<br>102932079
64		**pbi|*Python bivittatus***	103062296<br>103061809

			103056821<br>103050620
65	Amphibians	**xtr|*Xenopus tropicalis***	100493970<br>100487951<br>780266<br>594927
66	Fishes	**dre|*Danio rerio***	572251<br>566820<br>767686<br>100151141
67		**ola|*Oryzias latipes***	101169794<br>101154854<br>101163418<br>101159595
68		**lcm|*Latimeria chalumnae***	102365194<br>102347701<br>102359525<br>102349737
69		**cmk|*Callorhinchus milii***	103188810<br>103182148<br>103187056<br>103191318
70	Basal vertebrates	**cin|*Ciona intestinalis***	104265696
71		**spu|*Strongylocentrotus purpuratus***	585279
72	Diptera	**dme|*Drosophila melanogaster***	CG9381
73		**dpo|*Drosophila pseudoobscura pseudoobscura***	GA26288
74		**dya|*Drosophila yakuba***	GE24768
75		**dvi|*Drosophila virilis***	GJ10273
76		**mde|*Musca domestica***	101896752
77	Hymenoptera	**soc|*Solenopsis invicta***	105204511
78		**aec|*Acromyrmex echinatior***	105153293
79		**hst|*Harpegnathos saltator***	105180826
80		**cfo|*Camponotus floridanus***	105251768
81	Other insects	**tca|*Tribolium castaneum***	655967
82		**pxy|*Plutella xylostella***	105379982<br>105379980
83	Annelids	**hro|*Helobdella robusta***	101303<br>136586<br>192848
84	Mollusks	**lgi|*Lottia gigantea***	LOTGIDRAFT_88425<br>LOTGIDRAFT_159128
85		**crg|*Crassostrea gigas***	105339130<br>105337659<br>105338351
86	Basal animals	**hmg|*Hydra vulgaris***	100210297
87		**aqu|*Amphimedon queenslandica***	105312299
88	Fungi	**afm|*Aspergillus fumigatus***	AFUA_2G10860
89		**ang|*Aspergillus niger***	ANI_1_362024
90		**cim|*Coccidioides immitis***	CIMG_08704
91		**pbn|*Paracoccidioides brasiliensis* Pb18**	Pb18_PADG_01194
92		**aje|*Histoplasma capsulatum***	HCAG_08045
93		**pte|*Pyrenophora teres***	PTT_10342
94		**bsc|*Bipolaris sorokiniana***	COCSADRAFT_25649
95		**bor|*Bipolaris oryzae***	102184

96		**pfj|*Pseudocercospora fijiensis***	MYCFIDRAFT_87921
97		**tml|*Tuber melanosporum***	1229001
98		**spo|*Schizosaccharomyces pombe***	SPBP4H10.07<br>SPCC4G3.12c
99	Protists	**acan|*Acanthamoeba castellanii***	ACA1_157720<br>ACA1_171810
100		**tet|*Tetrahymena thermophila***	TTHERM_00530440<br>TTHERM_00469170<br>TTHERM_00607220
101		**ptm|*Paramecium tetraurelia***	GSPATT00034070001<br>GSPATT00037239001<br>GSPATT00008873001<br>GSPATT00001064001<br>GSPATT00010970001<br>GSPATT00027234001<br>GSPATT00005521001<br>GSPATT00024197001<br>GSPATT00020640001<br>GSPATT00005749001
102		**pif|*Phytophthora infestans***	PITG_12214
103		**psoj|*Phytophthora sojae***	PHYSODRAFT_484474
104		**tbr|*Trypanosoma brucei***	Tb927.8.3460
105		**tcr|*Trypanosoma cruzi***	509681.1<br>504087.1<br>507645.4<br>509767.22
106		**lma|*Leishmania major***	LMJF_23_1730
107		**lif|*Leishmania infantum***	LINJ_23_1780
108		**ldo|*Leishmania donovani***	LDBPK_231780
109		**lmi|*Leishmania mexicana***	LMXM_23_1730
110		**lbz|*Leishmania braziliensis***	LBRM_23_1630
