# Supplementary material for: CTLs, a new class of RING-H2 ubiquitin ligases uncovered by YEELL, a motif close to the RING domain that is present across eukaryotes
Source: PLoS One. 2018 Jan 11;13(1):e0190969. doi: 10.1371/journal.pone.0190969 (PMC5764321; doi:10.1371/journal.pone.0190969)
Supplement: S2 Table — (PDF) [file pone.0190969.s007.pdf]

# S2 Table. Distribution of CTLs retrieved from 33 angiosperm species in 8 groups.

| Species | A | B | C | D | E | F | G | H | Green algae |
|---|---|---|---|---|---|---|---|---|---|
| ppp | | | | | Pp3c19_21510V3.1 | | Pp3c5_17300V3.1 | | |
| | | | | | Pp3c22_1990V3.1 | | | | |
| smo | | SELMODRAFT_104716 | | | SELMODRAFT_448412 | | SELMODRAFT_19194 | | |
| | | SELMODRAFT_89607 | | | SELMODRAFT_447294 | | | | |
| atr | | scaffold00144.13 | scaffold00019.349 | | | scaffold00069.77 | scaffold00070.122 | | |
| | | scaffold00021.151 | | | | scaffold00022.365 | scaffold00041.165 | | |
| | | scaffold00034.19 | | | | | | | |
| spo | Spipo23G0034800 | Spipo31G0002500 | Spipo12G0057200 | | | Spipo2G0073400 | Spipo1G0062000 | | |
| | Spipo26G0022400 | Spipo0G0151900 | | | | | | | |
| | | Spipo10G0050600 | | | | | | | |
| | | Spipo10G0044400 | | | | | | | |
| | | Spipo10G0049700 | | | | | | | |
| bdi | Bradi2g46070.1 | Bradi2g16490.1 | Bradi3g18740.2 | Bradi2g34660.5 | Bradi2g47150.1 | Bradi5g18810.1 | Bradi3g42410.1 | | |
| | | Bradi5g01020.3 | Bradi5g20760.1 | | | Bradi2g34010.2 | Bradi4g36320.1 | | |
| | | | Bradi3g04020.1 | | | Bradi2g26690.1 | | | |
| | | | Bradi1g33750.1 | | | | | | |
| osa | | LOC_Os01g47740.13 | LOC_Os04g51400.2 | LOC_Os05g07070.1 | LOC_Os05g47670.1 | LOC_Os04g48260.1 | LOC_Os08g43480.1 | | |
| | | LOC_Os05g48970.1 | LOC_Os08g14320.1 | LOC_Os01g06590.2 | LOC_Os01g49770.1 | LOC_Os09g35690.1 | LOC_Os09g36460.1 | | |
| | | LOC_Os04g55510.1 | LOC_Os02g05692.1 | | | LOC_Os06g03580.2 | | | |
| | | LOC_Os04g10680.1 | LOC_Os06g48040.1 | | | LOC_Os03g07790.1 | | | |
| pvi | | Pavir.Eb02704.1 | Pavir.Fa01359.1 | Pavir.J01792.1 | Pavir.Ca01829.1 | Pavir.Ga00935.1 | Pavir.Fb02318.1 | | |
| | | Pavir.Ea02350.1 | Pavir.Fb01033.1 | Pavir.Ca01112.1 | Pavir.J23161.1 | Pavir.J30019.1 | Pavir.J13451.1 | | |
| | | Pavir.Ca00936.1 | Pavir.J17458.1 | Pavir.Eb00498.1 | Pavir.Ea02502.1 | Pavir.Ba01272.1 | Pavir.J20390.1 | | |
| | | Pavir.J09786.1 | Pavir.Gb00765.1 | Pavir.Ea00445.1 | Pavir.Eb02851.1 | Pavir.J04140.1 | | | |
| | | Pavir.J04843.1 | Pavir.Ab00398.1 | | | Pavir.J21784.1 | | | |
| | | Pavir.Gb00345.1 | Pavir.Aa03555.1 | | | Pavir.Db02385.1 | | | |
| | | Pavir.J03996.1 | Pavir.Da00148.1 | | | Pavir.Ib00467.1 | | | |
| | | Pavir.J06787.1 | Pavir.Db00274.1 | | | Pavir.Ia04338.1 | | | |
| sita | | Seita.5G263300.1 | Seita.6G090500.1 | Seita.7G272600.1 | Seita.3G158100.1 | Seita.7G204700.1 | Seita.6G238600.1 | | |
| | | Seita.3G146000.1 | Seita.7G226600.1 | Seita.5G113000.1 | Seita.5G280100.1 | Seita.2G281700.1 | Seita.2G288200.1 | | |
| | | Seita.7G262700.1 | Seita.1G087400.1 | | | Seita.4G015300.1 | | | |
| | | Seita.7G032800.1 | Seita.4G247100.1 | | | Seita.9G521900.1 | | | |
| sbi | | Sobic.003G250400.1 | Sobic.007G086900.1 | Sobic.009G056500.1 | Sobic.009G221600.1 | Sobic.006G184200.1 | Sobic.007G177801.1 | | |
| | | Sobic.009G232800.1 | Sobic.006G205100.1 | Sobic.003G061900.1 | Sobic.003G264901.1 | Sobic.002G270300.1 | Sobic.007G178100.1 | | |
| | | Sobic.006G240400.1 | Sobic.004G042100.1 | | | Sobic.010G015800.1 | Sobic.002G277300.1 | | |
| | | Sobic.006G027200.1 | Sobic.010G246000.1 | | | Sobic.001G487200.1 | | | |
| zma | | GRMZM2G124701_T01 | GRMZM2G138997_T01 | GRMZM2G118344_T03 | GRMZM2G061663_T01 | GRMZM2G081060_T01 | GRMZM2G477205_T01 | | |
| | | GRMZM2G120816_T02 | GRMZM2G140924_T02 | GRMZM2G305901_T01 | GRMZM2G122223_T01 | GRMZM2G073228_T01 | GRMZM2G058105_T01 | | |
| | | GRMZM2G020814_T01 | GRMZM2G053909_T02 | GRMZM2G165044_T01 | GRMZM5G843389_T01 | GRMZM2G108084_T01 | GRMZM2G085948_T01 | | |
| | | GRMZM2G174926_T01 | GRMZM2G392320_T01 | | | GRMZM2G021498_T01 | | | |
| | | GRMZM2G050973_T01 | GRMZM2G096211_T01 | | | GRMZM2G141084_T01 | | | |
| | | GRMZM2G000114_T01 | | | | | | | |
| aco | Aquca_051_00081.1 | Aquca_007_00704.1 | Aquca_031_00097.1 | Aquca_027_00056.1 | Aquca_041_00093.1 | Aquca_013_00410.1 | Aquca_014_00904.1 | | |
| | | Aquca_016_00083.1 | Aquca_024_00101.1 | | | Aquca_014_00832.1 | Aquca_052_00012.1 | | |
| | | Aquca_015_00023.1 | | | | Aquca_009_00200.1 | | | |
| mgu | Migut.E00374.1 | Migut.C00560.1 | Migut.A00438.1 | Migut.J00432.1 | Migut.D01604.1 | Migut.F01141.1 | Migut.H00809.1 | | |
| | Migut.H01966.1 | Migut.N02748.1 | | Migut.L01674.1 | | Migut.F00369.1 | | | |
| | Migut.M00762.1 | | | | | Migut.B01311.1 | | | |
| | | | | | | Migut.D00588.1 | | | |
| sly | Solyc12g014070.1.1 | Solyc12g088740.1.1 | Solyc12g096420.1.1 | Solyc09g007530.2.1 | Solyc03g118840.2.1 | Solyc03g026150.2.1 | Solyc12g010500.1.1 | | |
| | Solyc10g007140.2.1 | Solyc04g078680.2.1 | Solyc08g006460.2.1 | | Solyc12g040390.1.1 | Solyc01g087060.2.1 | | | |
| | Solyc07g062720.1.1 | Solyc10g050170.1.1 | | | | Solyc11g062260.1.1 | | | |
| | | Solyc01g107940.2.1 | | | | | | | |
| | | Solyc01g107930.2.1 | | | | | | | |
| stu | PGSC0003DMT400039631 | PGSC0003DMT400039849 | PGSC0003DMT400075959 | PGSC0003DMT400004449 | PGSC0003DMT400014732 | PGSC0003DMT400037132 | PGSC0003DMT400020398 | | |
| | PGSC0003DMT400054920 | PGSC0003DMT400020528 | PGSC0003DMT400067670 | | PGSC0003DMT400078539 | PGSC0003DMT400017553 | | | |
| | PGSC0003DMT400032385 | PGSC0003DMT400066252 | | | | PGSC0003DMT400082375 | | | |
| | | PGSC0003DMT400079217 | | | | | | | |
| vvi | GSVIVT01014254001 | GSVIVT01012873001 | GSVIVT01035519001 | GSVIVT01016299001 | GSVIVT01008343001 | GSVIVT01020798001 | GSVIVT01026184001 | | |
| | GSVIVT01036994001 | GSVIVT01009466001 | | GSVIVT01018684001 | GSVIVT01011840001 | GSVIVT01032082001 | GSVIVT01008838001 | | |
| | | GSVIVT01031717001 | | GSVIVT01025655001 | | GSVIVT01025294001 | | | |
| | | GSVIVT01022104001 | | | | | | | |
| | | GSVIVT01022103001 | | | | | | | |
| | | GSVIVT01022107001 | | | | | | | |
| | | GSVIVT01022106001 | | | | | | | |
| | | GSVIVT01022110001 | | | | | | | |
| egr | Eucgr.F01182.1 | Eucgr.F02323.1 | Eucgr.C02025.1 | Eucgr.K03219.1 | Eucgr.B00829.1 | Eucgr.J01268.1 | Eucgr.G02582.1 | | |
| | | Eucgr.F00204.1 | | | | Eucgr.J00783.1 | | | |
| | | Eucgr.I01135.1 | | | | Eucgr.J00781.1 | | | |
| pop | Potri.011G097100.1 | Potri.002G124300.1 | Potri.006G274700.1 | Potri.010G220700.1 | Potri.012G053800.1 | Potri.009G062100.2 | Potri.005G090800.1 | | |
| | Potri.001G373700.1 | Potri.001G304900.1 | Potri.018G005600.1 | | Potri.015G043900.1 | Potri.001G267700.1 | Potri.007G073200.1 | | |
| | | Potri.009G100100.1 | | | | Potri.003G204500.1 | Potri.002G080900.1 | | |
| | | Potri.005G139900.1 | | | | Potri.001G019600.1 | | | |
| | | Potri.007G045500.1 | | | | Potri.012G136400.1 | | | |
| | | | | | | Potri.015G138700.1 | | | |
| lus | | Lus10024810 | Lus10042443 | Lus10040389 | Lus10007967 | Lus10019703 | Lus10017385 | | |
| | | Lus10010372 | Lus10026941 | Lus10023507 | Lus10013497 | Lus10016416 | Lus10010182 | | |
| | | | Lus10020145 | | Lus10021722 | Lus10031275 | Lus10005768 | | |
| | | | | | Lus10034644 | Lus10031842 | | | |
| | | | | | | Lus10002554 | | | |
| rco | 30209.m001522 | 30170.m013851 | 29848.m004449 | 30078.m002352 | 29747.m001086 | 29794.m003495 | 29634.m002088 | | |
| | | 29801.m003161 | | | | 29212.m000184 | 29648.m002011 | | |

| | | | | | | | | |
|---|---|---|---|---|---|---|---|---|
| | | 30115.m001237 | | | | | | |
| csi | orange1.1g009501m | orange1.1g005363m<br>orange1.1g004784m<br>orange1.1g002483m<br>orange1.1g047787m | orange1.1g008677m | orange1.1g016305m | orange1.1g016946m | orange1.1g026092m<br>orange1.1g025212m<br>orange1.1g045064m | orange1.1g019980m | |
| cpa | supercontig_53.60<br>supercontig_841.2 | supercontig_21.175 | supercontig_23.14 | supercontig_45.53 | supercontig_3.460 | | | supercontig_55.128<br>evm.TU.contig_32935.1<br>supercontig_55.127<br>evm.TU.contig_30435.1 |
| tca | Thecc1EG031665t1 | Thecc1EG033800t1<br>Thecc1EG007084t1<br>Thecc1EG000650t1 | Thecc1EG036819t1 | Thecc1EG043109t3<br>Thecc1EG022121t1 | Thecc1EG012485t1 | Thecc1EG005889t1<br>Thecc1EG040623t1<br>Thecc1EG015103t1 | Thecc1EG007711t1 | |
| aha | Araha.41851s0002.1<br>Araha.17302s0003.1 | Araha.7279s0001.1<br>Araha.2852s0002.1<br>Araha.22084s0001.1 | Araha.10572s0006.1<br>Araha.8919s0009.1<br>Araha.11917s0004.1 | Araha.14169s0021.1 | Araha.23688s0001.1<br>Araha.19609s0006.1<br>Araha.18459s0005.1 | Araha.35247s0006.1<br>Araha.18769s0004.1<br>Araha.33550s0004.1<br>Araha.0297s0009.1 | Araha.2452s0002.1 | |
| aly | 478936<br>314643 | 491267<br>931278<br>331106<br>473842 | 489350<br>487900 | 482704 | 332860<br>476516<br>312759 | 949768<br>483242<br>495284<br>324777 | 479509 | |
| ath | AT1G53190.1_CTL01<br>AT3G15070.1_CTL02 | AT2G15530.4_CTL03<br>AT4G34040.1_CTL04<br>AT1G45180.1_CTL05<br>AT5G42940.1_CTL06 | AT5G24870.1_CTL07<br>AT5G10650.1_CTL08<br>AT4G31450.1_CTL09 | AT2G37150.3_CTL10 | AT5G67120.1_CTL11<br>AT1G73760.1_CTL14<br>AT1G17970.1_CTL15 | AT5G52140.1_CTL17<br>AT3G63530.1_CTL19 | AT3G47180.1_CTL16<br>AT3G19910.1_CTL18 | AT4G00070.1_CTL12<br>AT1G36950.1_CTL13 |
| bst | Bostr.28625s0166.1<br>Bostr.0124s0112.1 | Bostr.7867s1256.1<br>Bostr.18351s0028.1<br>Bostr.3148s0190.1<br>Bostr.12302s0119.1 | Bostr.7867s1000.1<br>Bostr.5763s0032.1<br>Bostr.13175s0036.1 | Bostr.23794s0375.1 | Bostr.0568s0134.1<br>Bostr.3288s0019.1<br>Bostr.7128s0404.1 | Bostr.7305s0054.1<br>Bostr.13083s0052.1<br>Bostr.13158s0358.1 | Bostr.19424s0671.1 | |
| bra | Brara.E02634.1<br>Brara.C03555.1<br>Brara.F00132.1<br>Brara.E01471.1 | Bradi5g24120.1<br>Brara.G00487.1<br>Brara.I00979.1<br>Brara.I01765.1<br>Brara.F03830.1<br>Brara.J00595.1<br>Brara.H00442.1 | Brara.K01654.1<br>Brara.F02695.1<br>Brara.B00366.1<br>Brara.C00432.1<br>Brara.J02292.1 | Brara.C01860.1<br>Brara.D02257.1 | Brara.G01196.1<br>Brara.G03241.1<br>Brara.G02303.1<br>Brara.H02443.1 | Bradi1g49490.3<br>Brara.B01529.1<br>Brara.F03694.1<br>Brara.J00759.1<br>Brara.D00004.1<br>Bradi1g73040.1 | Brara.A02844.1<br>Brara.E02214.1<br>Brara.F01936.1 | |
| cgr | Cagra.0007s0049.1<br>Cagra.1472s0010.1 | Cagra.1305s0029.1<br>Cagra.1957s0003.1 | Cagra.3807s0017.1<br>Cagra.0622s0006.1<br>Cagra.1398s0005.1 | Cagra.0666s0030.1 | Cagra.0876s0008.1<br>Cagra.2175s0028.1 | Cagra.5140s0001.1<br>Cagra.0664s0082.1 | Cagra.0316s0076.1<br>Cagra.0448s0054.1 | |
| cru | Carubv10013526m<br>Carubv10011258m | Carubv10004296m<br>Carubv10026015m<br>Carubv10012632m<br>Carubv10008591m | Carubv10004669m<br>Carubv10000716m | Carubv10022961m | Carubv10020494m<br>Carubv10009526m | Carubv10027729m<br>Carubv10018505m | Carubv10014137m | |
| esa | Thhalv10020603m<br>Thhalv10011416m | Thhalv10022580m<br>Thhalv10003162m<br>Thhalv10011314m<br>Thhalv10024583m | Thhalv10025002m<br>Thhalv10004038m<br>Thhalv10013256m | Thhalv10016485m | Thhalv10005473m<br>Thhalv10018747m<br>Thhalv10007935m | Thhalv10015344m | Thhalv10021098m | |
| csa | Cucsa.122810.1<br>Cucsa.273400.1 | Cucsa.395980.1 | Cucsa.161760.1<br>Cucsa.311030.1 | | Cucsa.121730.1 | Cucsa.350640.1<br>Cucsa.127830.1<br>Cucsa.143280.1<br>Cucsa.065490.1 | | |
| gmx | Glyma.11G156300.1<br>Glyma.06G234300.1<br>Glyma.12G158500.1<br>Glyma.13G272700.1 | Glyma.06G041000.1<br>Glyma.04G039800.1<br>Glyma.04G039700.1<br>Glyma.02G103300.1<br>Glyma.07G214600.1<br>Glyma.17G240900.1 | Glyma.14G134600.1<br>Glyma.17G198300.1<br>Glyma.06G076600.1 | Glyma.19G180000.1<br>Glyma.03G179300.1<br>Glyma.10G051200.1<br>Glyma.13G138700.1 | Glyma.05G241900.1<br>Glyma.08G049400.1<br>Glyma.09G180300.1<br>Glyma.07G097700.1 | Glyma.12G237700.1<br>Glyma.13G203300.1<br>Glyma.11G132700.1<br>Glyma.12G057100.1 | Glyma.10G284900.1<br>Glyma.20G104300.1<br>Glyma.04G249100.1 | Glyma.04G075400.1 |
| mtr | Medtr1g019200.2<br>Medtr2g078440.1<br>Medtr2g076290.1<br>Medtr2g076240.1<br>Medtr2g078450.2 | Medtr4g085630.1 | Medtr1g009680.1<br>Medtr3g108808.3 | Medtr7g100950.1<br>Medtr1g066400.1<br>Medtr1g066430.1 | Medtr8g106240.1<br>Medtr6g082620.1 | Medtr8g036640.1<br>Medtr8g037150.1<br>Medtr4g065670.2 | Medtr1g114240.2<br>Migut.E00691.1 | |
| pvu | Phvul.011G118000.1<br>Phvul.011G149000.1<br>Phvul.011G153200.1<br>Phvul.010G052600.1<br>Phvul.006G043800.1<br>Phvul.006G035700.1<br>Phvul.010G083100.1<br>Phvul.010G083200.1<br>Phvul.005G068800.1 | Phvul.009G066000.1<br>Phvul.001G023300.1 | Phvul.001G059300.1<br>Phvul.009G101200.1 | Phvul.003G242800.1<br>Phvul.007G221700.1<br>Phvul.001G176000.1 | Phvul.002G325200.1<br>Phvul.004G153500.1 | Phvul.011G213300.1<br>Phvul.011G058800.1<br>Phvul.005G148200.1 | Phvul.007G015800.1<br>Phvul.001G072100.1 | |
| pper | Prupe.4G172400.1 | Prupe.7G256200.1<br>Prupe.8G127800.1<br>Prupe.1G432700.1 | Prupe.1G460200.1 | Prupe.6G238800.1 | Prupe.5G225100.1 | Prupe.2G012300.1<br>Prupe.4G244600.1 | Prupe.7G271700.1<br>Prupe.1G493000.1 | |
| cre | | | | | | | | Cre10.g442750.t1.2 |
| vca | | | | | | | | Vocar20008863m |
| csl | | | | | | | | 60820 |
| mpp | 50363 | | | | | | | |
| mis | | | | | | | | 61535 |
| olu | | | | | | | | 38024 |
