# Supplementary material for: CTLs, a new class of RING-H2 ubiquitin ligases uncovered by YEELL, a motif close to the RING domain that is present across eukaryotes
Source: PLoS One. 2018 Jan 11;13(1):e0190969. doi: 10.1371/journal.pone.0190969 (PMC5764321; doi:10.1371/journal.pone.0190969)

S3 Table. Catalog of sequence LOGOs generated from vertebrates, invertebrates, fungi, protists and plants.

LOGO Number	Sequence
[3]	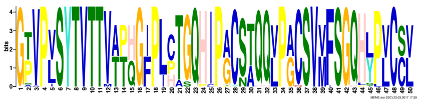
[4]	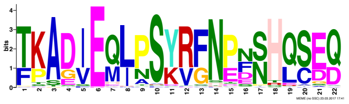
[5]	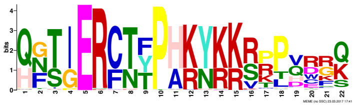
[6]	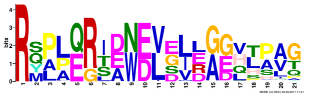
[7]	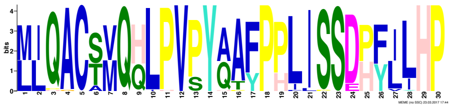
[8]	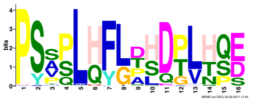
[9]	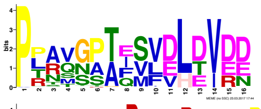
[10]	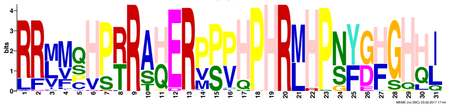
[11]	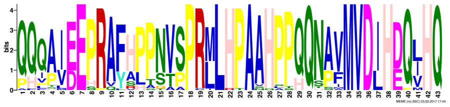
[12]	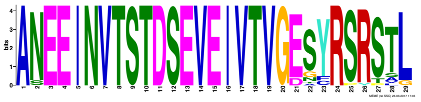
[13]	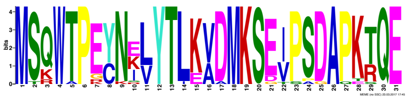
[14]	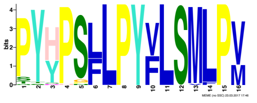
[15]	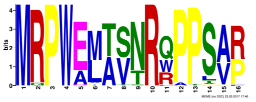
[16]	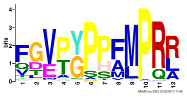

[17]

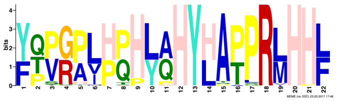

[18]

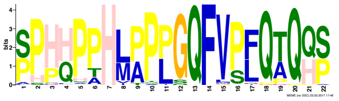

[19]

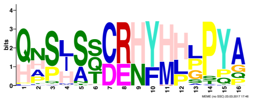

[20]

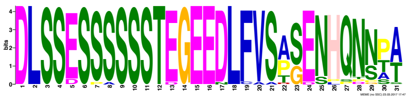

[21]

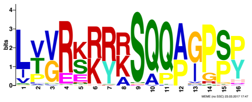

[22]

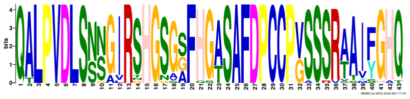

[23]

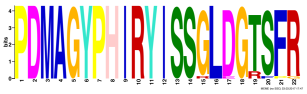

[24]

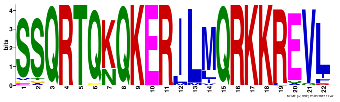

[25]

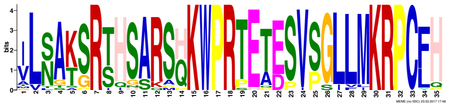

[26]

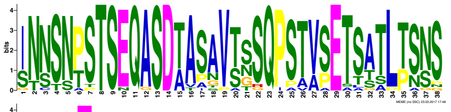

[27]

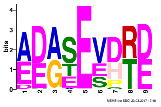

[28]

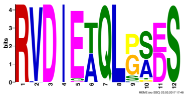

[29]

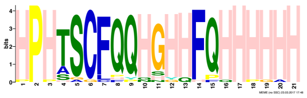

[30]

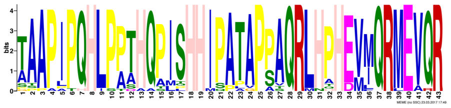

[31]

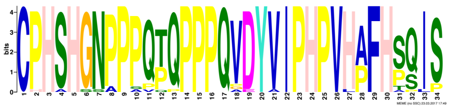

[32]

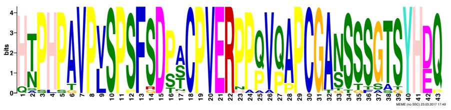

[33]

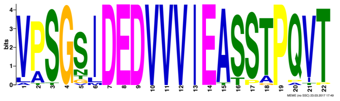

[34]

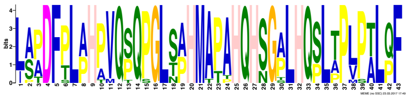

[35]

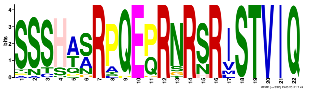

[36]

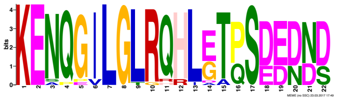

[37]

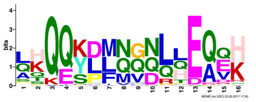

[38]

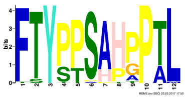

[39]

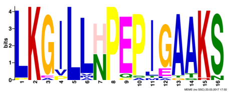

[40]

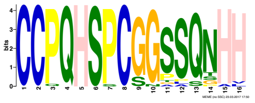

[41]

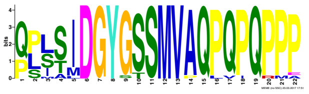

[42]

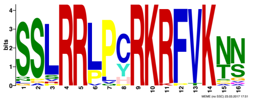

[43]

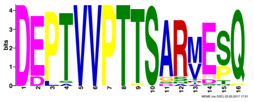

[44]

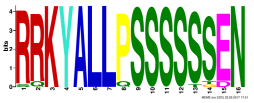

[45]

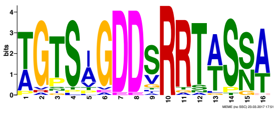

[46]

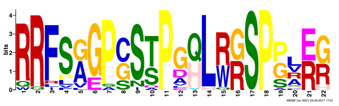

[47]

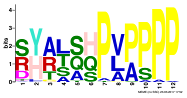

[48]

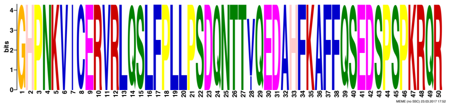

[50] 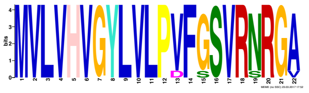

[51] 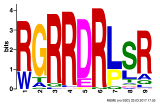

[52] 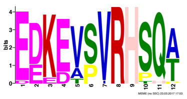

[54] 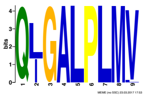

[55] 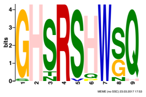

[57] 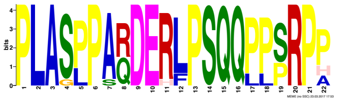

[58] 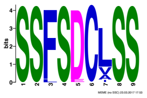

[59] 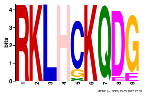

[60] 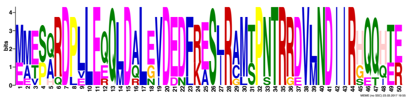

[61] 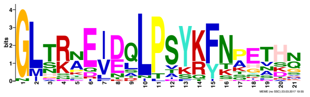

[62] 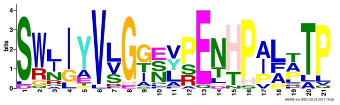

[63] 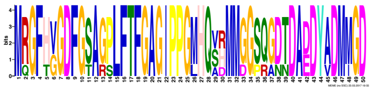

[64] 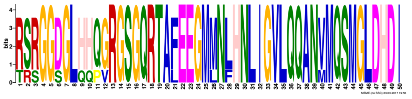

[65] 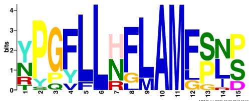

[66] 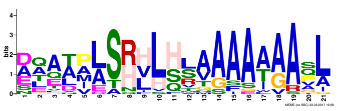

[67] 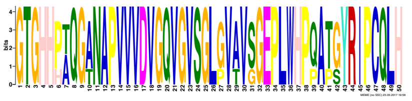

[69]

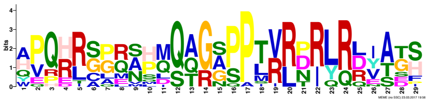

[70]

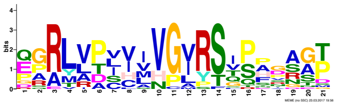

[71]

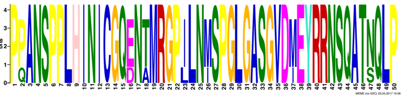

[72]

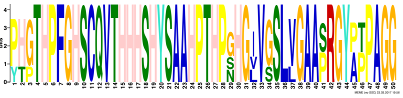

[73]

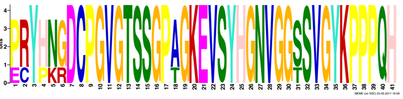

[74]

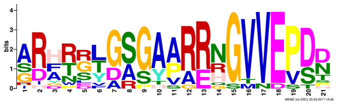

[75]

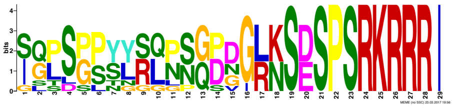

[77]

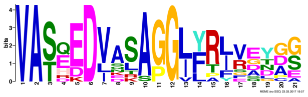

[78]

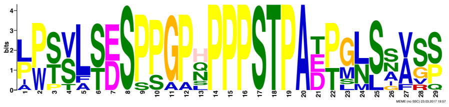

[79]

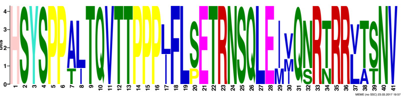

[80]

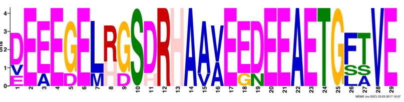

[81]

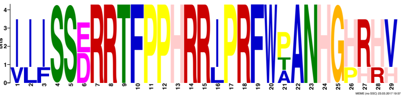

[82]

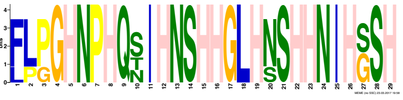

[83]

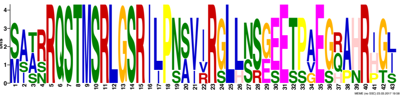

[84]

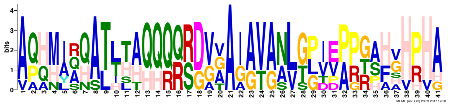

[85]

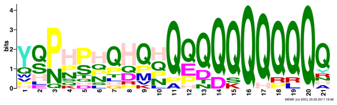

[86]

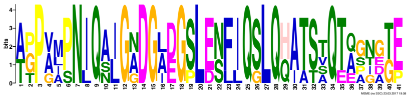

[87]

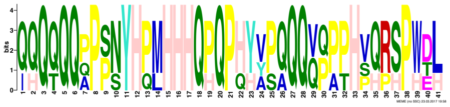

[88]

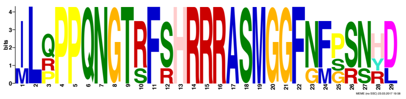

[89]

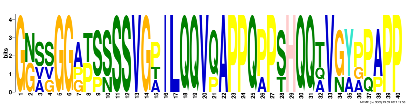

[90]

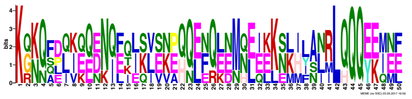

[92]

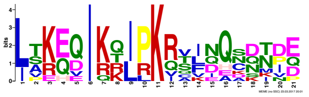

[93]

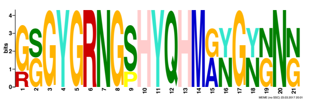

[94]

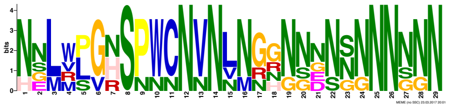

[95]

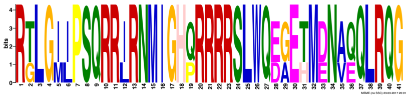

[96]

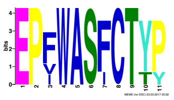

[97]

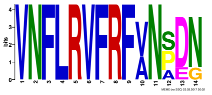

[98]

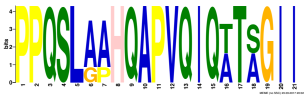

[99]

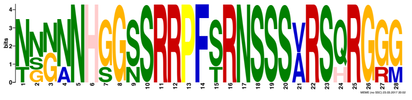

[100]

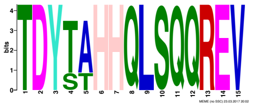

[102]

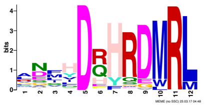

[103]

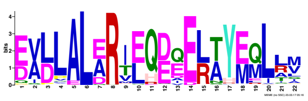

[104]

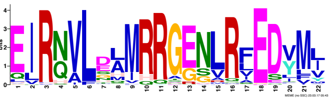

[105]

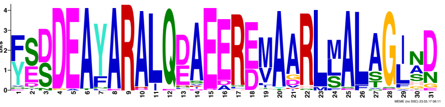

[106]

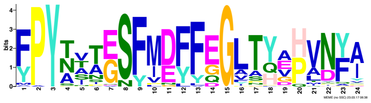

[107]

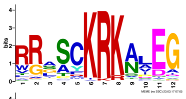

[108]

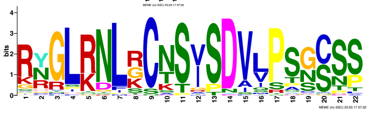

[109]

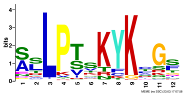

[110]

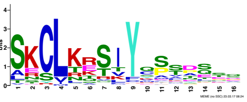

[111]

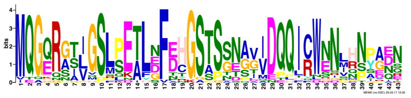

[112]

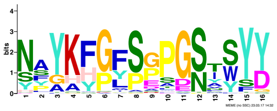

[113]

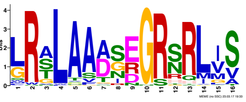

[114]

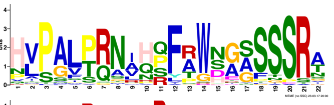

[116]

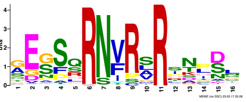

[117]

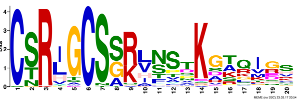

[118]

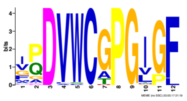

[119]

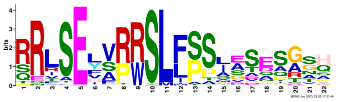

[120]

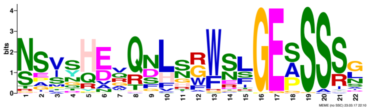

[121]

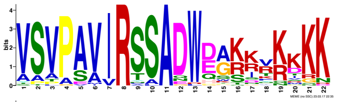

[122]

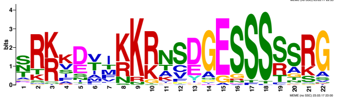

[123]

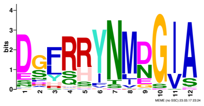

[124]

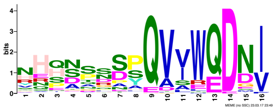

[126]

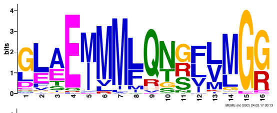

[127]

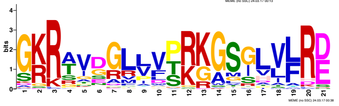

[128]

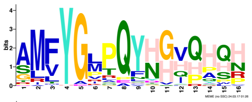

[129]

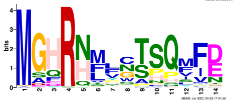

[130]

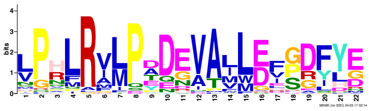

[131]

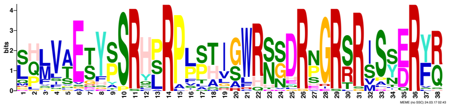

[132]

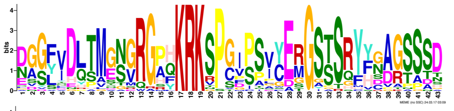

[133]

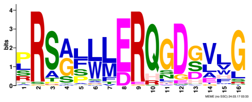

[134]

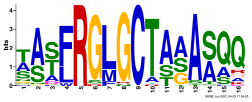

[135]

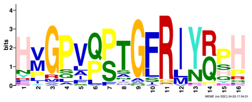

[136]

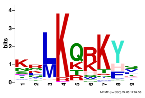

[137]

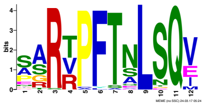

[138]

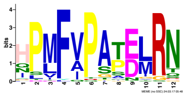

[139]

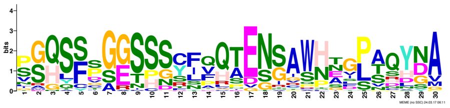

[140]

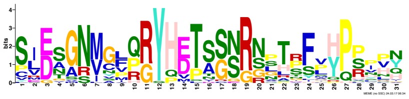

[141]

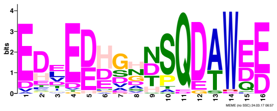

[142]

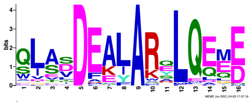

[143]

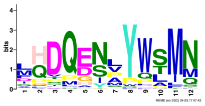

[144]

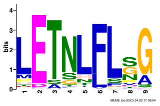

[145]

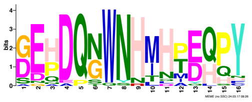

[146]

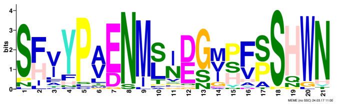

[147]

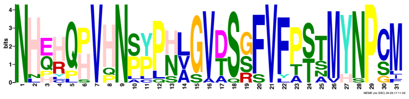

[148]

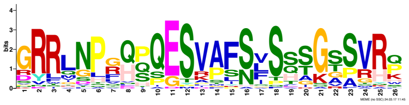

Supplement: S3 Table — (PDF) [file pone.0190969.s008.pdf]
