# Supplementary material for: CTLs, a new class of RING-H2 ubiquitin ligases uncovered by YEELL, a motif close to the RING domain that is present across eukaryotes
Source: PLoS One. 2018 Jan 11;13(1):e0190969. doi: 10.1371/journal.pone.0190969 (PMC5764321; doi:10.1371/journal.pone.0190969)
Supplement: S4 Table — (PDF) [file pone.0190969.s009.pdf]

S4 Table. Oligonucleotides used for cloning YEELL containing fragments into pGBK-T7

Gene	Restriction site	Oliogonucleotide sequences (5' to 3')
AthCTL 2	*Eco*RI	Forward: CGGAATTCTCATACAGTCATATGGGACAGGTTC
	*Bam*HI	Reverse: CGGGATCCACCTTCTTCCAGGTTAATTCTGGT
AthCTL6	*Eco*RI	Forward: CGGAATTCGGATAGTGTGGTTGGTATTCCTCA
	*Bam*HI	Reverse: CGGGATCCTTTTCGTTGCTTTAACCGGTTTG
AthCTL8	*Eco*RI	Forward: CGGAATTCTACTGGACGCGTACATAGC
	*Bam*HI	Reverse: CGGGATCCAACCGGTTTCATCTGTCTC
AthCTL10	*Nde*I	Forward: CCATATGGGTCTCATTTGGTCACTGAG
	*Sal*I	Reverse: CCCTCGAGCTAACAAGCAACTGGAGATTGC
AthCTL14	*Eco*RI	Forward: CGGAATTCCGATGGAGATAAAACCAAT
	*Bam*HI	Reverse CGGGATCCCTTGCCGGCATGGTTTGAC
AthCTL18	*Eco*RI	Forward: CGGAATTCAGAAGTTGCTTATACAGAT
	*Sal*I	Reverse: CCCTCGAGTCTGATTGTCTCCTTCTTTATA
AthCTL19	*Eco*RI	Forward: CGGAATTCTGATGATCCTGAAAACACAGTTGC
	*Bam*HI	Reverse: CGGGATCCAGAAGATGCTCCCAAACTTA
